# Liu-Shen-Wan inhibits PI3K/Akt and TRPV1 signaling alleviating bone cancer pain in rats

**DOI:** 10.1080/15384047.2024.2432098

**Published:** 2024-11-25

**Authors:** Hui Zhang, Jingwen Jiang, Xuewu Chen, Fengting Zhu, Fangfang Fu, Aiying Chen, Lei Fu, Dan Mao

**Affiliations:** a Department of Oncology, Guangdong Provincial Hospital of Traditional Chinese Medicine, Hainan Hospital, Haikou, Hainan, China; b Department of Oncology, Affiliated Hainan Traditional Chinese Medicine Hospital, Guangzhou University of Chinese Medicine, Haikou, Hainan, China; c Department of Dermatology, Guangdong Provincial Hospital of Traditional Chinese Medicine, Hainan Hospital, Haikou, Hainan, China; d Department of Integrated Traditional Chinese and Western Medicine, the Second Xiangya Hospital, Central South University, Changsha, Hunan, China

**Keywords:** Bone cancer pain, Bufalin, Liu-Shen-Wan, PI3K/Akt, TRPV1

## Abstract

Patients with advanced-stage cancers often suffer from severe pain caused by bone metastasis and destruction, for which effective treatment options are limited. Liu-Shen-Wan (LSW) is a widely recognized herbal formula utilized for pain relief. This study aims to elucidate the effects of LSW on bone cancer pain (BCP). In this study, the pharmacology of LSW on BCP was screened by network pharmacology. A BCP model was conducted using Walker 256 cells. Paw withdrawal threshold and paw withdrawal latency were employed as measures to assess the pain threshold in rats. The pathways and cell types of LSW against BCP were explored. Next, the impact of LSW on Walker 256 cells was evaluated, and UPLC-MS was utilized to identify the active ingredients of LSW. Furthermore, the effects of the key active ingredient, Bufalin, on the BCP rats were evaluated. There were 275 shared targets between LSW and BCP, which were enriched in neural tissue ligand-receptor interaction pathway. LSW increased pain threshold and decreased inflammatory cytokines levels in BCP rats by inhibiting PI3K/Akt and transient receptor potential vanilloid 1 (TRPV1) signaling through astrocytes and microglia. LY294002 further alleviated BCP in rats, while the effects were reversed after treatment with insulin-like growth factor 1 (IGF-1). Both LSW and its active ingredient Bufalin were shown to inhibit the viability and migration of Walker 256 cells and induce apoptosis. Bufalin appears to be the key active ingredient of LSW and exerts its pain-relieving effects by suppressing PI3K/Akt and TRPV1 signaling in BCP.

## 1. Introduction

Bone cancer pain (BCP) is a common complication resulting primarily from tumor metastasis to the bone.^1^ Factors contributing to the development of BCP include damage to sensory nerve fibers, infiltration of nerve roots and cancer cells, and the presence of pain-causing biological agents, which lead to neurochemical reorganization and persistent cancer-related pain.^2^ Prolonged activation generates ongoing neural signals that induce changes in the spinal cord, contributing to the progression of BCP.^3^ Consequently, addressing neuropathic pain may be a crucial initial step in managing BCP. Currently, several treatment options are available for the treatment of BCP, such as nonsteroidal anti-inflammatory drugs, opioid analgesia, external beam radiation therapy, and anesthesia techniques.^1,4^ However, due to the limitations of these existing treatments, many BCP patients experience inadequate pain relief and adverse side effects. Therefore, there is an urgent need to explore innovative strategies for the treatment of BCP.

Traditional Chinese medicine (TCM) has a long-standing history of managing cancer pain through the use of natural pharmacological agents. A Meta-analysis from Yan *et al*. revealed that randomized controlled trials (RCTs) involving 534 patients demonstrated significant benefits of applying external TCM treatments, such as *Camphol* and *Angelica*, in alleviating pain in individuals with BCP.^5^ Furthermore, when compared to conventional treatments like radiotherapy or bisphosphonates, seven RCTs involving 521 patients demonstrated the effectiveness of kushen injection in improving pain relief among patients with BCP.^6^ In addition, the utilization of TCM Xiao-Ai-Tong has been shown to effectively mitigate pain and minimize negative side effects associated with morphine treatment in patients with BCP.^7^ Moreover, there is potential value in using Liu-Shen-Wan (LSW) for BCP. LSW has the effects of relieving pain, clearing away heat detoxifying, and reducing swelling.^8^ Studies have demonstrated that LSW exhibits analgesic and anti-inflammatory properties.^9^ Ma *et al*. have reported that LSW inhibits influenza a virus and excessive virus-induced inflammatory response via suppression of TLR4/NF-κB signaling pathway.^10^ Moreover, LSW has been found to inhibit influenza virus-induced secondary Staphylococcus aureus infection.^11^ However, the specific role of LSW in managing BCP remains uncertain and deserves further investigation.

The phosphoinositide 3-kinase (PI3K)/protein kinase B (Akt) signaling pathway is recognized for its significant role in the progression of BCP resulting from bone metastasis.^12^ Activation of the PI3K/Akt pathway within the spinal cord has been identified as a contributing factor to the development of hyperalgesia in various neuropathic conditions, including spinal nerve ligation,^13^ peripheral nerve injury,^14^ and specific inflammatory states.^15,16^ Moreover, transient receptor potential vanilloid 1 (TRPV1), a nonselective cation channel, has been found to interact with various intracellular proteins *in vivo*, such as PI3K/Akt.^17^ In addition, TRPV1 activation has been associated with neuroprotective effects.^18^ Nevertheless, the precise involvement of the PI3K/Akt pathway in BCP and its potential relationship with TRPV1 remains to be fully elucidated.

In the current study, we explored the effects of LSW on BCP both *in vitro* and *in vivo*, alongside an analysis of the underlying mechanism. Moreover, the therapeutic properties of Bufalin, the active ingredient of LSW, were investigated. This study provided innovative insights into the management of BCP.

## 2. Materials and methods

### 2.1. Network pharmacology

#### 2.1.1. Components target finding

The components of LSW, including *toad venom*, *bezoar*, *musk*, *borneol*, *pearl powder*, and *realgar*, were queried in the Batman-TCM database (http://bionet.ncpsb.org.cn/batman-tcm/) to identify their respective component targets. Targets with a probability score exceeding 40 were selected for further analysis. Subsequently, all identified targets were compared with the UniProt database (https://www.uniprot.org/) to eliminate targets that are not of human origin.

#### 2.1.2. Disease target finding

To identify the human genes associated with BCP, an extensive investigation was carried out across three databases: GeneCards (https://www.genecards.org/.), NCBI (https://www.ncbi.nlm.nih.gov/.), and OMIM (https://www.omim.org/). The keyword “bone cancer pain” was used as the search criterion, and the genes obtained from these databases were consolidated, resulting in a total of 5120 genes associated with the disease.

#### 2.1.3. Venn diagram and protein-protein interaction (PPI) network construction

The 275 targets identified from the combined component and disease target searches were represented visually through the utilization of Venny 2.1, a software application for generating Venn diagrams. The shared targets between the components and disease were subsequently analyzed using the String database (https://string-db.org/cgi/input.pl) to establish a PPI network, with a confidence threshold exceeding 0.9. The biological species selected for this analysis was “Homo sapiens”.

#### 2.1.4. Kyoto encyclopedia of genes and genomes (KEGG) enrichment analysis

The shared targets associated with both components and disease were analyzed for KEGG enrichment using the String database. A screening threshold of a *P*-adjust value below 0.05 was applied to identify significant KEGG pathways. The results were then graphically represented as histograms using the clusterProfiler package in R version 4.0.3.

#### 2.1.5. Component-disease-pathway-target network construction

To enhance understanding of the complex interactions between various components, disease, their respective targets, and related pathways, a network diagram was created. This diagram depicted the connections between ingredients, therapeutic disease, pathways, and targets using Cytoscape 3.8.0 software. Following this, a topology analysis was performed to organize the ingredients according to their degree, with the importance of an ingredient being directly related to its degree level.

### 2.2. Animals care

Male specific-pathogen-free Sprague Dawley rats, weighing approximately 200 g, were ordered from Hunan SJA Laboratory Animal Co., Ltd (Changsha, China). The rats were kept in a pathogen-free environment with a 12-h light/dark cycle, maintained at a temperature of 22 ± 1°C and a humidity level of approximately 50%. They had ad libitum access to food and water and were allowed to acclimate for one week. The Institutional Animal Care and Use Committee of Hainan Hospital of Traditional Chinese Medicine approved the animal experiments (No. IACUC-HPHCM-2210001).

### 2.3. BCP model

The rats were placed in a supine position and securely attached to the operating table, with the skin around the left knee joint sterilized. A longitudinal incision of 0.5 cm was made below the knee joint using scissors, exposing the muscle tissue. Following fixation of the knee joint, a 7-gauge needle was inserted longitudinally from the humeral distal end toward the tibia on the humeral surface, reaching a depth of approximately 1.5 cm. Next, 10 μL of Walker 256 cells (1 × 10^7^/mL) were injected into the rat tibia bone marrow cavity with a microinjection needle.^19^ The needle was removed, and the opening was promptly sealed with bone wax. The surgical incision was closed using sutures following irrigation with a normal saline solution. Paw withdrawal threshold (PWT) and paw withdrawal latency (PWL) were measured on the 21st day, and peripheral blood samples were collected for further assays. Subsequently, rats were injected intravenously with barbiturates (150 mg/kg) and then sacrificed. The brain tissues and spinal dorsal horn tissues were then harvested for subsequent detection.

### 2.4. Animal experiments

LSW (Batch No. Z32020481) was obtained from Leiyunshang Pharmaceutical Co., Ltd (Suzhou, China). To investigate the role of PI3K involvement in the therapeutic effects of LSW exerted on BCP, we utilized LY294002 (154447-36-6) and insulin-like growth factor 1 (IGF-1, P1016), which was purchased from APExBIO (Houston, Texas, USA). Notably, LY294002 is a PI3K inhibitor and IGF-1 is a PI3K agonist.^20^ The rats were randomly divided into five groups (*n* = 6 per group): Sham group, Model group, LSW group, LSW+LY294002 group, and LSW+IGF-1 group. Rats in the LSW, LSW+LY294002, and LSW+IGF-1 groups were orally administered LSW at a dosage of 0.02 g/kg/d once daily for three weeks. The rats were anesthetized by intraperitoneal injection of 1% pentobarbital (50 mg/kg). On the 17th, 19th, and 21st days following the implantation of Walker 256 cells, rats in the LSW+LY294002 group were subjected to intrathecal injections of 20 μL LY294002 (0.25 μg/μL).^21^ Concurrently, rats in the LSW+IGF-1 group received intrathecal injections of 20 μL IGF-1 (0.36 μg/μL).^21^ The rats in the Sham, Model, and LSW groups were administered an intrathecal injection of 20 μL saline as a control. After three weeks, rats were euthanized by intraperitoneal injection of 1% pentobarbital (150 mg/kg).

To further explore the effects of Bufalin on BCP rats, the rats were randomly divided into five groups (*n* = 6 per group): Sham group, Model group, Bufalin group, Bufalin+LY294002 group, and Bufalin+IGF-1 group. Bufalin was purchased from Chengdu Alfa Biotechnology Co., Ltd., (China, CAS 465-21-4). Rats in the Bufalin, Bufalin+LY294002, and Bufalin+IGF-1 groups were administered Bufalin intraperitoneally at a dose of 0.5 mg/kg once daily for three weeks.^22^ The administration of other treatments remained consistent with the aforementioned protocol.

### 2.5. Western blotting

Walker 256 cells and spinal dorsal horn tissues were harvested and lysed using RIPA lysate (AWB0136, Abiowell, Changsha, China). Following centrifugation at 12,000 rpm for 15 min, the resulting supernatant was transferred to a 1.5 mL centrifuge tube. Proteins were separated through sodium dodecyl sulfate (SDS) gel electrophoresis and then transferred to nitrocellulose membranes. These membranes were blocked using a 5% bovine serum albumin (BSA, Saibao Biotechnology Co., Ltd. Yancheng, China) and subsequently incubated with primary antibodies overnight at 4°C. The primary antibodies used included PI3K (ab191606, Abcam, Cambridge, MA, USA), p-PI3K (ab278545, Abcam), Akt (10176–2-AP, Proteintech, Rosemont, IL, USA), p-Akt (Ser473) (28731–1-AP, Proteintech), glial fibrillary acidic protein (GFAP, 16825–1-AP, Proteintech), ionized calcium binding adaptor molecule 1 (Iba1, ab178847, Abcam), extracellular signal-regulated kinase (ERK, 16443–1-AP, Proteintech), p-ERK (Thr202/Tyr204) (28733–1-AP, Proteintech), and TRPV1 (ab203103, Abcam). Next, the membranes were exposed to the secondary antibodies HRP goat anti-mouse IgG (AWS0001, Abiowell) and HRP goat anti-rabbit IgG (AWS0002, Abiowell) for 90 min at room temperature. Following this, the membranes were treated with ECL chemiluminescence solution (AWB0005, Abiowell) for 1 min and visualized using a chemiluminescence imaging system (ChemiScope6100, CLiNX, Shanghai, China). The internal reference protein utilized in this study was β-actin (66009–1-Ig, Proteintech). ImageJ software was employed for the quantification and comparison of western blotting band intensities.

### 2.6. Quantitative reverse-transcription polymerase chain reaction (qRT-PCR)

Total RNA was isolated from the tissue samples using Trizol reagent (15596026, Thermo Fisher Scientific, Waltham, MA, USA). Subsequently, cDNA was synthesized employing an mRNA reverse transcription kit (CW2569, CWBIO, Beijing, China). The expression levels of the target genes were detected utilizing the UltraSYBR Mixture kit (CW2601, CWBIO) and a fluorescence quantitative PCR instrument (PIKOREAL96, Thermo Fisher Scientific). Primers were designed utilizing Primer 5 software (PREMIER Biosoft, San Francisco, CA, USA) (Table 1). β-actin was used as an internal control gene. The quantification of target gene expression was determined through the 2^−ΔΔCt^ method.

**Table 1.** Primer sequences.

Gene Name	5’-3’	Product length (bp)
β-actin	**F**: ACATCCGTAAAGACCTCTATGCC	223
	**R**: TACTCCTGCTTGCTGATCCAC	
PI3K	**F**: AGCCACAGATCCACTTAACCC	128
	**R**: CTTGCTGTCCCCACTTTACTGA	
Akt	**F**: GTCACCTCTGAGACCGACACC	115
	**R**: GCCTCCGTTCACTGTCCAC	
ERK	**F**: GCTGCTGTGTCTTTATCTATCCC	103
	**R**: CTCCACCCCTCTGTAGCAC	
TRPV1	**F**: GCCAGAGGATGCTGAGGTTT	105
	**R**: GTTCCCTAAGCAGACCACCC	

### 2.7. Behavioral test

In the PWT assay, rats were acclimated to a quiet setting for 15–30 min. Before the test, the rats exhibited behaviors such as refraining from seeking, defecating, urinating, or perching on their hind legs. A vertical stimulation was applied to the sole of the left hind foot of the rat using a probe, with the intensity of the stimulation gradually increasing. This elicited a response from the rat such as foot withdrawal, the raising of the foot, or rapid licking. The maximum pressure required to induce the paw withdrawal reflex was recorded. The stimulation was repeated until three consistent test values were recorded.

In the PWL assay, rats were placed on a preheated hot plate. The time taken for the hind paw withdrawal or paw licking to occur was considered as the pain threshold of rats. Each rat underwent three measurements with a minimum interval of 5 min between each measurement. The average of these three measurements was then calculated as the pain threshold for thermal stimulation in the animal.^23^

### 2.8. Cell culture and treatment

Walker 256 cells (iCell-r038, iCell Bioscience Inc, Shanghai, China), a rat breast carcinoma cell line, were cultured in DMEM-high glucose medium (D5796, Sigma-Aldrich, St. Louis, MO, USA) supplemented with 10% fetal bovine serum (FBS; 10099141, Gibco, Grand Island, NY, USA). The cell cultures were maintained in a CO_2_ incubator at 37°C. Upon reaching 80%–90% confluence, the cells were divided into two experimental groups: (1) Control group, receiving normal treatment, and (2) LSW group, where cells were exposed to LSW (5 µL/mL, with a concentration of 20.09 µg/mL^8^) for 24 h.

### 2.9. Enzyme-linked immunosorbent assay (ELISA)

ELISA was performed to quantify the concentrations of IL-1β, IL-6, and TNF-α in both rat serum and spinal cord tissue. This analysis utilized specific ELISA kits in accordance with the guidelines provided by the respective manufacturers. The ELISA kits employed were as follows: TNF-α (KE20001, Proteintech), IL-6 (BMS625, Invitrogen, Carlsbad, CA, USA), and IL-1β (BMS630, Invitrogen).

### 2.10. Immunocytochemistry (IHC)

IHC was performed to assess the expression of astrocyte marker GFAP and microglia marker Iba1 in the CA1 region of the hippocampus within brain tissue. The process involved immersing dehydrated sections in xylene for 1 h, followed by dehydration using a gradient of alcohol. Antigen retrieval was achieved through treatment with citrate buffer (0.01 M, pH = 6.0), and endogenous enzymes were inactivated using periodic acid (1%). The primary antibodies (GFAP, 16825–1-AP, Proteintech; Iba1, 10904–1-AP, Proteintech) and secondary antibody were subsequently applied in sequence. Following treatment with diaminobenzidine (DAB) and hematoxylin, the sections underwent another round of dehydration. Finally, the sections were observed after immersion in xylene and blocking with neutral gum.

### 2.11. Immunofluorescence (IF)

The dehydrated sections were treated with xylene for 1 h, followed by a gradient of alcohol for further dehydration. Subsequently, the primary antibodies (GFAP, AWA10155, Abiowell; Iba1, AWA12684, Abiowell) and secondary antibody were applied sequentially. Nuclei were stained using a DAPI working solution. Buffered glycerol was used to seal the tissue slices. The slices were then examined under a fluorescence microscope (BA410T, Motic, Xiamen, China).

### 2.12. Cell counting kit-8 (CCK-8)

Cells were distributed into the 96-well plates at a density of 1 × 10^4^ cells per well. The experiment was conducted using the CCK-8 kit (NU679, Dojindo, Kumamoto, Japan). The absorbance at 450 nm, denoted as optical density (OD), was measured with the Bio-Tek microplate reader (MB-530, Heales, Shenzhen, China).

### 2.13. Transwell assay

Transwell chambers (3428, Corning Incorporated, Corning, NY, USA) were utilized in the study. The lower compartment of the chambers was filled with 500 μL of 10% FBS complete medium to facilitate cell migration. A cell suspension containing 2 × 10^6^ cells/mL (100 μL) was added to each well and incubated at 37°C for 48 h. Subsequently, the upper chamber underwent a washing procedure, where cells were gently removed using cotton balls and fixed with a 4% paraformaldehyde solution. The cells were then stained with crystal violet and observed under a microscope (BA410T, Motic, Xiamen, China). The OD value was determined using a Bio-Tek microplate reader (MB-530, Heales).

### 2.14. Cell apoptosis assay

To evaluate apoptosis in Walker 256 cells, the Annexin V-propidium iodide (PI) apoptosis detection kit (KGA1030, KeyGEN BioTech, Nanjing, China) was used. The cells were resuspended in a binding buffer and labeled with Annexin V and PI for 10 min at room temperature under light-protected conditions. Subsequently, the samples were analyzed using a FACSCanto Flow Cytometer (A00-1-1102, Beckman Coulter, Brea, CA, SA).

### 2.15. Preparation of LSW-contained serum

Rats were administered an oral dose of LSW solution (8.4 mg/kg) once daily. Following a 3-day treatment period, the rats were euthanized, and blood samples were obtained for further analysis. Serum samples were isolated through centrifugation, subsequently filtered using a 0.22 μm filter unit, and finally stored at −80°C for preservation.

### 2.16. Ultra-performance liquid chromatography-mass spectrometry (UPLC-MS)

The rat serum sample (200 μL) was combined with a pre-chilled overnight mixture of methanol and ethylene glycol (1:1) in a ratio of 600 μL. Following vortexing, the supernatant was dried by vacuum centrifuge. Subsequently, an acetonitrile (80)/methanol (20) -water (1:1) mixture was added to the dried sample, which was then dissolved, vortexed, and centrifuged at 14,000 rpm at 4°C for 15 min. A 4 μL portion of the resulting supernatant was collected for further analysis. Additionally, 500 μL of LSW concentrate was mixed with 500 μL of ethanol and centrifuged at 18,000×g for 5 min. Another concentrate (500 μL) was treated with ultrapure water (500 μL) and centrifuged at 18,000×g for 5 min. The combined supernatants were filtered using a 0.22 μm filter for subsequent analysis. The analysis was conducted using the TurboIonSpray ion source (AB SCIEX, Boston, MA, USA), Quadrupole time-of-flight mass spectrometer (X-500 R QTOF, AB SCIEX), and ACQUITY I-Class Plus UPLC ultra-performance liquid chromatography system (Waters, Milford, MA, USA). The chromatography column utilized was the Acquity UPLC BEH C18 (150 × 2.1 mm^2^, 1.7 μm), with solution A consisting of 0.1% formic acid in acetonitrile and solution B being a 0.1% aqueous formic acid solution. The flow rate was set at 0.3 mL/min with a 2 μL injection volume. The sample tray and column were maintained at temperatures of 8°C and 40°C, respectively. TurboIonSpray ion sources were applied for time-of-flight MS with ESI positive and negative ion scan modes. MS-DIAL 4.10 software was used to analyze the data.

### 2.17. Molecular docking

The molecular structure of Bufalin was obtained from PubChem Compound (https://www.ncbi.nlm.nih.gov/pccompound.), while the crystal structures of PI3K were sourced from the RCSB Protein Data Bank (https://www.rcsb.org/). Both Bufalin and PI3K were transformed into pdbqt formats using AutoDockTools 1.5.6 (CCSB, La Jolla, California, USA). Subsequently, molecular docking analysis was carried out employing Autodock Vina 1.1.2 (CCSB, La Jolla), with the docking process executed through the Genetic Algorithm. The docking outcomes with the highest scores were then visualized using Discovery Studio (BIOVIA, Vélizy-Villacoublay, France).

### 2.18. Statistical analysis

The statistical analysis in this study was conducted using Graphpad Prism 9.0. The data is expressed as the mean ± standard deviation. Prior to conducting comparisons, tests were performed to assess normality and homogeneity of variance. An unpaired *t*-test was utilized for group comparisons, while a one-way analysis of variance (ANOVA) was employed for comparisons involving multiple groups. A significance level of *p* < .05 was considered indicative of statistical significance.

## 3. Results

### 3.1. Network pharmacological analysis was conducted to explore the potential relationship between LSW and BCP

**Figure 1.**
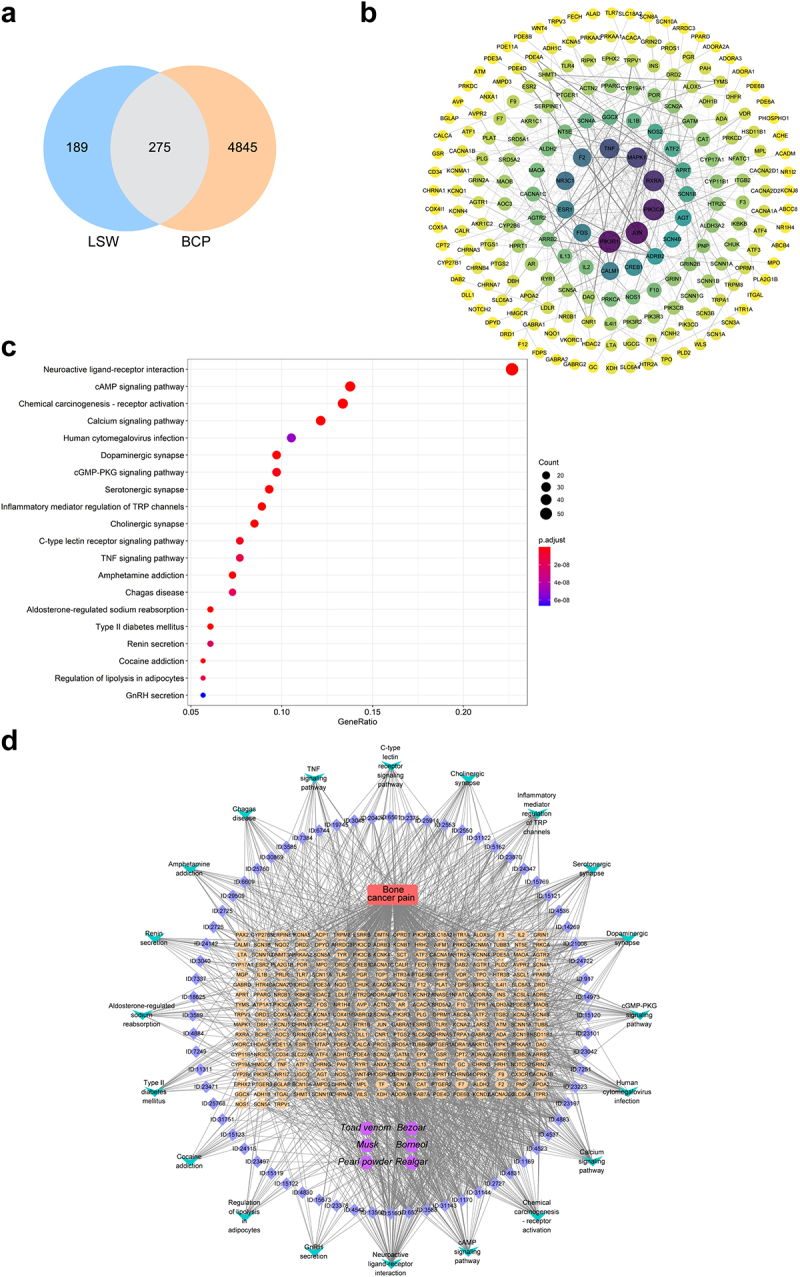
Network pharmacological analysis was conducted to explore the potential relationship between LSW and BCP. (a) A Venn diagram was utilized to visually represent the shared targets between LSW and BCP. (b) The PPI network was established to depict shared targets of LSW and BCP. (c) KEGG pathway analysis was conducted. (d) The component-disease-pathway-target network diagram was constructed. The purple, blue, red, orange, and green nodes represent compounds, active ingredients, diseases, target genes, and pathways, respectively.

Previous studies have indicated that LSW possesses analgesic and anti-inflammatory properties,^9,10,24^ suggesting a potential therapeutic effect on BCP. Therefore, a network pharmacological analysis was conducted to investigate the relationship between LSW and BCP. The analysis involved matching 464 LSW-related targets with 5120 BCP-related targets, resulting in the identification of 275 targets as potential candidates for LSW in the treatment of BCP (Figure 1a). A PPI network was constructed using Cytoscape to identify hub genes targeted by LSW in BCP treatment, revealing key targets such as PIK3R1, JUN, PIK3CA, RXRA, and MAPK1 (Figure 1b). The involvement of the PI3K signaling pathway in LSW’s regulation of BCP was highlighted. KEGG enrichment analysis was conducted to explore the therapeutic pathways of the potential targets, with the neuroactive ligand-receptor interaction pathway being significantly affected (Figure 1c). A component-disease-pathway-target network was constructed to elucidate the molecular mechanism underlying LSW’s efficacy in BCP treatment, providing a comprehensive understanding of the interconnections (Figure 1d). Network topology analysis identified several key active ingredients of LSW against BCP, including Deoxycholic acid, Adrenaline, Cholicacid, Androsterone, 3beta-Hydroxy-5alpha-Androstan-17-One, 3alpha-Hydroxy-5alpha-Androstan-17-One, Bufothionine, and Decamine, based on their higher degrees within the network (Table 2).

**Table 2.** Key ingredients information.

Name	Average Shortest Path Length	Betweenness Centrality	Closeness Centrality	Degree
Deoxycholic acid	2.684814	0.007158	0.372465	45
Adrenaline	2.759312	0.011564	0.362409	44
Cholic acid	2.707736	0.006412	0.369312	42
Androsterone	2.690544	0.005179	0.371672	41
3beta-Hydroxy-5alpha-Androstan-17-One	2.690544	0.005179	0.371672	41
3alpha-Hydroxy-5alpha-Androstan-17-One	2.690544	0.005179	0.371672	41
Bufothionine	2.782235	0.007	0.359423	40
Decamine	2.770774	0.008252	0.36091	40

### 3.2. LSW inhibited PI3K/Akt and TRPV1 signaling in walker 256 cells and BCP rats

**Figure 2.**
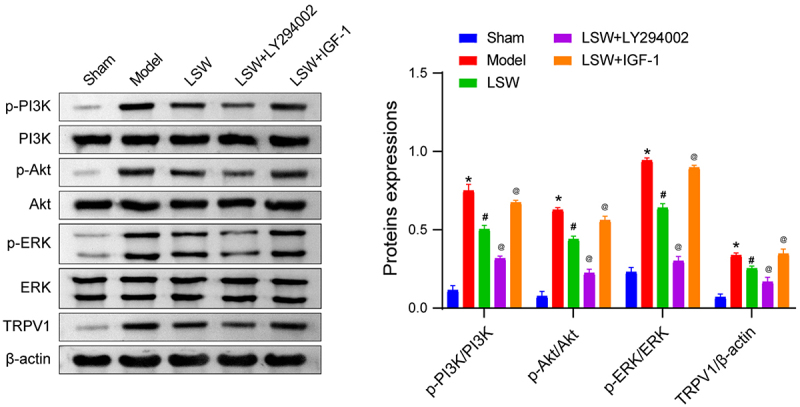
LSW inhibited PI3K/Akt and TRPV1 signaling pathways. The levels of p-PI3K/PI3K, *p*-Akt/Akt, p-ERK/ERK, and TRPV1 in the spinal dorsal horn of rats with BCP. The original blots are presented in supplementary figure S1. *n*=6 per group. *p < .05 vs. Sham. ^#^*p* < .05 vs. Model. ^@^*p* < .05 vs. LSW.

The network pharmacology analysis indicated that LSW could potentially have therapeutic benefits for BCP by modulating the PI3K signaling pathway. To investigate this further, we evaluated the expression of the PI3K/Akt signaling pathway in the spinal dorsal horn of rats. Our findings from qRT-PCR and western blotting analyses revealed a significant decrease in the expression of the PI3K/Akt pathway in the LSW group compared with the Model group (Figure 2). Consistent with expectations, treatment with LY294002, a specific inhibitor of PI3K, led to a reduction in the expression of the PI3K/Akt signaling pathway, while the application of IGF-1, a known activator of PI3K,^25^ resulted in an increase in pathway expression. Additionally, the expression of ERK, a pivotal downstream protein of the PI3K/Akt pathway, exhibited similar trends. Previous studies have indicated that TRPV1 in the dorsal root ganglion is involved in BCP,^26^ with its mechanism linked to the activation of the PI3K/Akt pathway.^27^ Consequently, we also assessed the expression of TRPV1 in the spinal dorsal horn of rats. Our results demonstrated a significant increase in TRPV1 expression in BCP rats, which was restored upon LSW administration (Figure 2). Additionally, co-administration of LY294002 enhanced the efficacy of LSW and reduced TRPV1 expression. In contrast, the administration of IGF-1 counteracted the effects of LSW, leading to elevated TRPV1 levels. These findings suggest that LSW inhibits the activation of PI3K/Akt and TRPV1 signaling in the spinal dorsal horn of BCP rats, potentially mediating therapeutic effects on BCP through these pathways.

### 3.3. The effects of LSW on the BCP rat model

**Figure 3.**
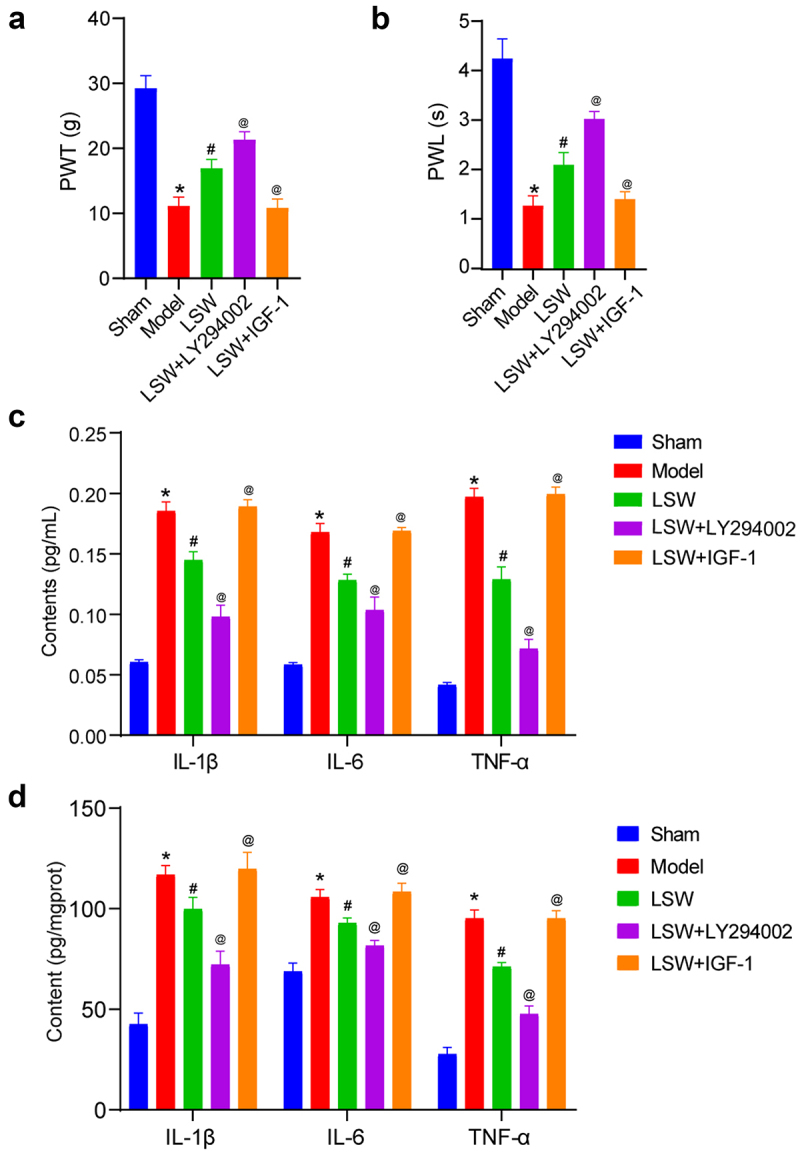
LSW effectively delayed the occurrence of BCP in rats. (a)-(b) PWT and PWL measurements were conducted in BCP rats. (c) The concentrations of IL-1β, IL-6, and TNF-α in the serum of rats were measured. (d) The concentrations of IL-1β, IL-6, and TNF-α in the spinal cord L4–6 of rats were measured. *n*=6 per group. *p < .05 vs. Sham. ^#^*p* < .05 vs. Model. ^@^*p* < .05 vs. LSW.

In this study, an investigation was conducted to determine the impact of LSW on BCP in rats. The presence of persistent pain responses in rats afflicted with bone cancer was confirmed through a notable increase in both mechanical and thermal sensitivity.^28^ Pain assessment was conducted by measuring the PWT and PWL. Following the injection of Walker 256 cells for three weeks, a significant decrease in PWT and PWL was observed. Nevertheless, oral administration of LSW resulted in elevated PWT and PWL, indicating the analgesic properties of LSW in BCP rats. In addition, it was observed that LY294002 further enhanced PWT and PWL, while IGF-1 reversed the effects of LSW (Figures 3a and 3b). Subsequent analysis involved the assessment of inflammatory cytokine levels in rat serum. The ELISA results revealed that LSW effectively reduced the heightened levels of IL-1β, IL-6, and TNF-α induced by BCP, demonstrating the anti-inflammatory attributes of LSW in BCP rats. Furthermore, LY294002 was found to decrease these cytokine levels, whereas IGF-1 reversed the effects of LSW, leading to an increase in inflammatory mediators (Figure 3c). Moreover, the impact of LSW on the regulation of inflammatory factors in the spinal cord L4–6 of BCP rats was investigated. As shown in Figure 3d, the levels of IL-1β, IL-6, and TNF-α in the spinal cord of BCP rats were significantly elevated but were mitigated following oral administration of LSW. Treatment with the PI3K inhibitor LY294002 further reduced the levels of these inflammatory factors, whereas the PI3K agonist IGF-1 promoted the inflammatory response. These findings suggest that LSW exerts analgesic and anti-inflammatory effects by PI3K/Akt signaling pathway in BCP rats.

### 3.4. LSW inhibited the expressions of GFAP and Iba1 in BCP rats

**Figure 4.**
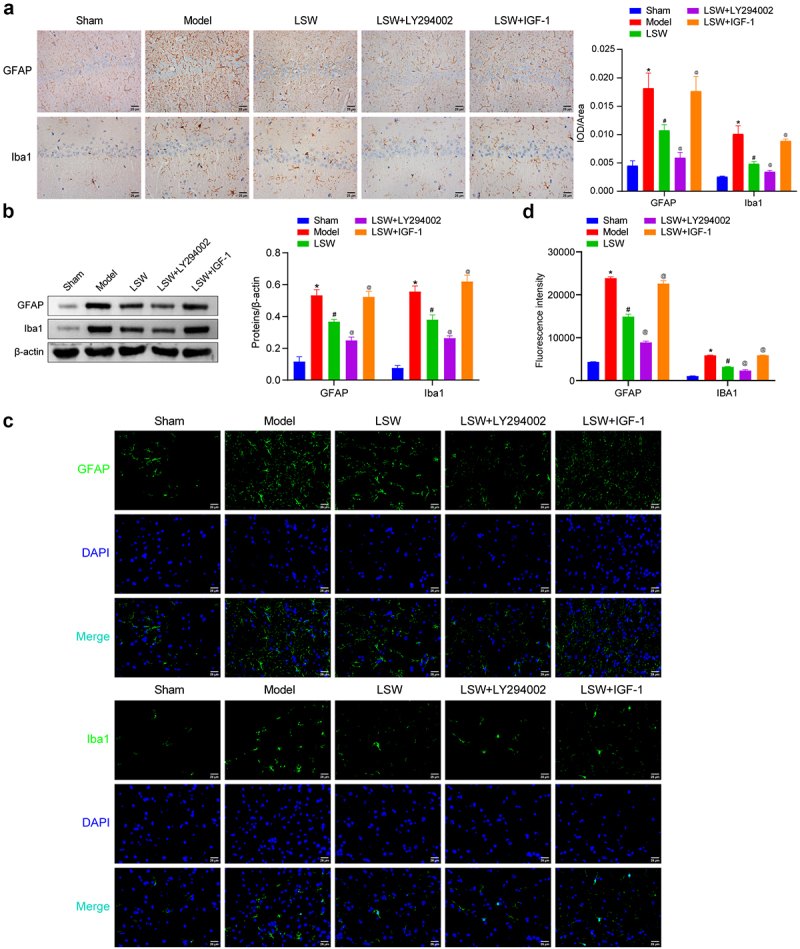
LSW inhibited the expressions of GFAP and Iba1 in BCP rats. (a) The astrocyte and microglia marker GFAP and Iba1 expressions were evaluated in brain tissues, respectively. (b) The expressions of GFAP and Iba1 were examined in the spinal dorsal horn of rats. The original blots are presented in supplementary figure S1. (c-d) The expressions of GFAP and Iba1 were examined using IF in the spinal dorsal horn of rats. *n*=6 per group. *p < .05 vs. Sham. ^#^*p* < .05 vs. Model. ^@^*p* < .05 vs. LSW.

During the progression of BCP, the presence of bone tumors may lead to damage to peripheral nerves, subsequently causing sensitization of primary sensory neurons and activation of glial cells.^29^ Research has shown that astrocytes and microglia in the central nervous system of BCP rats play a significant role in pain mechanisms.^30,31^ To identify the specific cell types influencing BCP, we conducted IHC using cell-specific markers GFAP (astrocytes) and Iba1 (microglia). The analysis of brain tissues from BCP rats revealed a notable increase in GFAP and Iba1 expression in the CA1 region of the hippocampus, which was reduced by treatment with LSW (Figure 4a). This suggests that LSW may inhibit the activation of astrocytes and microglia in BCP rats. Western blotting analysis of the spinal dorsal horn in BCP rats produced similar results, with LSW showing effectiveness that was further enhanced by LY294002 and reversed by IGF-1 (Figure 4b). IF results of GFAP and Iba1 expression in the spinal dorsal horn supported these findings (Figures 4c and 4d). These results suggest that microglia and astrocytes may contribute to the development of BCP in rats through the PI3K/Akt signaling pathway.

### 3.5. Effects of LSW on Walker 256 cells

**Figure 5.**
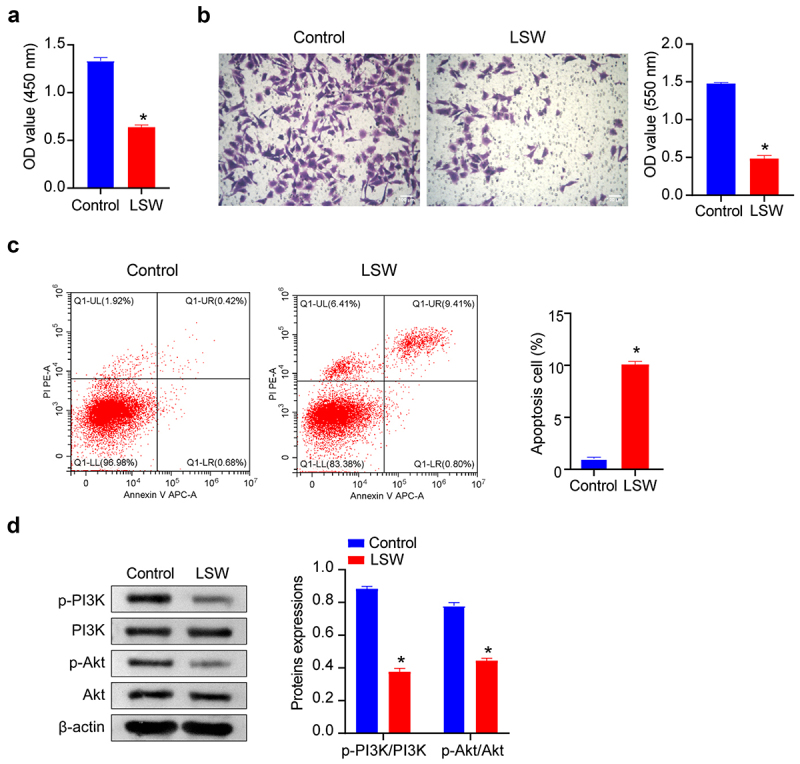
Effects of LSW on Walker 256 cells. (a) The cell viability of Walker 256 cells was examined after exposure to LSW for 24 h. (b) Transwell assay of Walker 256 cells following treatment with LSW for 24 h. (c) Flow cytometry was employed to measure apoptosis of Walker 256 cells following treatment with LSW for 24 h. (d) The effects of LSW on the levels of p-PI3K/PI3K and p-Akt/Akt in Walker 256 cells. The original blots are presented in supplementary figure S1. *n*=3 per group. *p < .05 vs. Control.

Our investigation then focused on examining the regulatory effects of LSW on Walker 256 cells subsequent to LSW treatment. The assessment of cell viability of Walker 256 cells was conducted using the CCK-8 assay. As illustrated in Figure 5a, the results indicated a decrease in the viability of Walker 256 cells after treatment with LSW, indicating an inhibitory effect of LSW on the proliferation of these cells. Additionally, outcomes from the Transwell assay revealed a suppression in the migration of Walker 256 cells by LSW (Figure 5b). From the apoptosis detection, we found that LSW promoted apoptosis of Walker 256 cells (Figure 5c). Furthermore, as displayed in Figure 5d, the levels of p-PI3K/PI3K and p-Akt/Akt were notably diminished following treatment with LSW, indicating an inhibition of the PI3K/Akt signaling pathway by LSW. Therefore, LSW was found to impede the proliferation and migration of Walker 256 cells while promoting apoptosis through the PI3K/Akt pathway.

### 3.6. Identification of ingredients of LSW and LSW-contained serum

**Figure 6.**
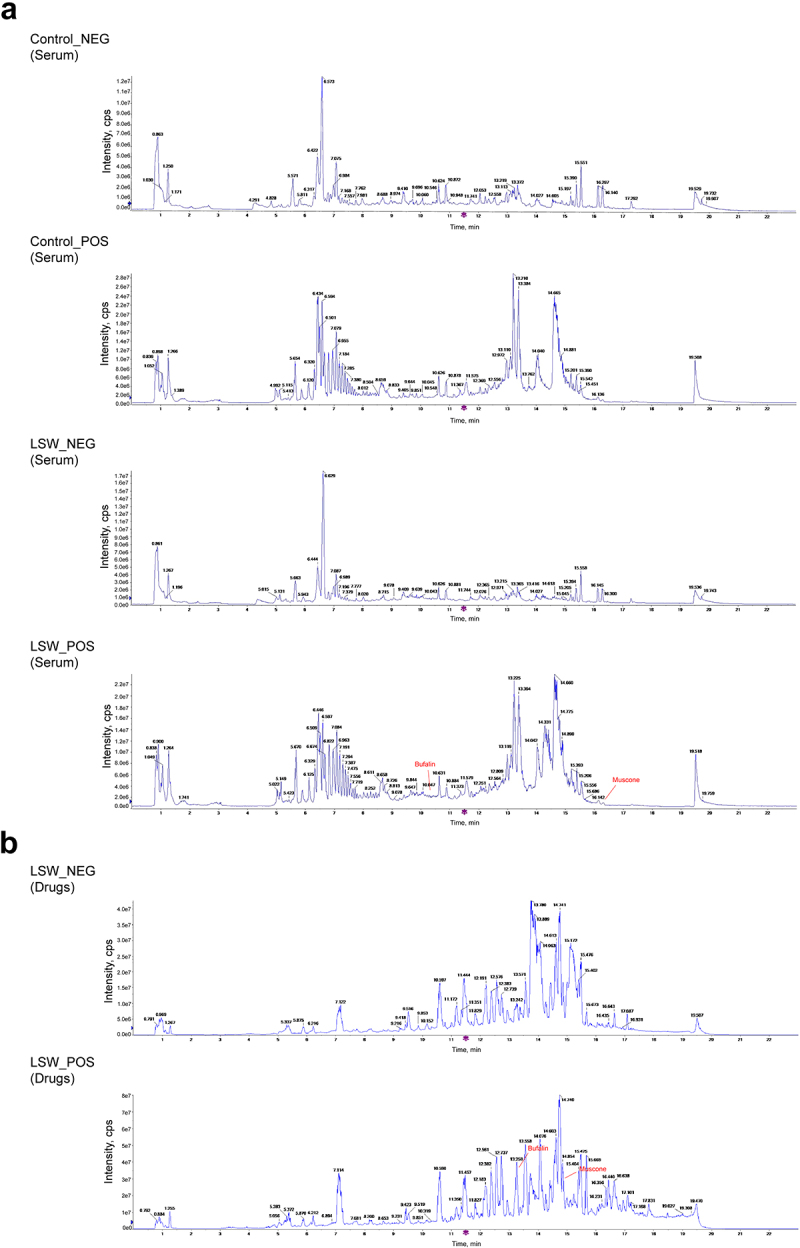
Identification of ingredients of LSW and LSW-containing serum. (a) UPLC-MS identification of LSW-contained serum. (b) UPLC-MS identification of LSW.

Following this, a UPLC-MS analysis was conducted on both LSW and LSW-contained serum to determine the bioactive ingredients within LSW. Several shared abundant ingredients were detected in both LSW and LSW-contained serum, as outlined in Table S3-S6, such as Cholan-24-oic acid, Linoleic acid, and Taurine. This discovery indicates that the therapeutic constituents of LSW exhibit favorable oral bioavailability in healthy animals. Furthermore, Bufalin and Muscone, which were not found in rat serum untreated with LSW, were identified in LSW-contained serum, in addition to Deoxycholic acid, the top-scoring compound in network pharmacology analysis (Figure 6a, Table S1-S4). These aforementioned ingredients were also present in LSW, as illustrated in Figure 6b and detailed in Table S5-S6. Therefore, it can be deduced that Bufalin and Muscone serve as discrete active ingredients present in both LSW and LSW-contained serum, suggesting that the effects of LSW might be modulated by these active ingredients.

### 3.7. The effects of Bufalin on the BCP rat model

**Figure 7.**
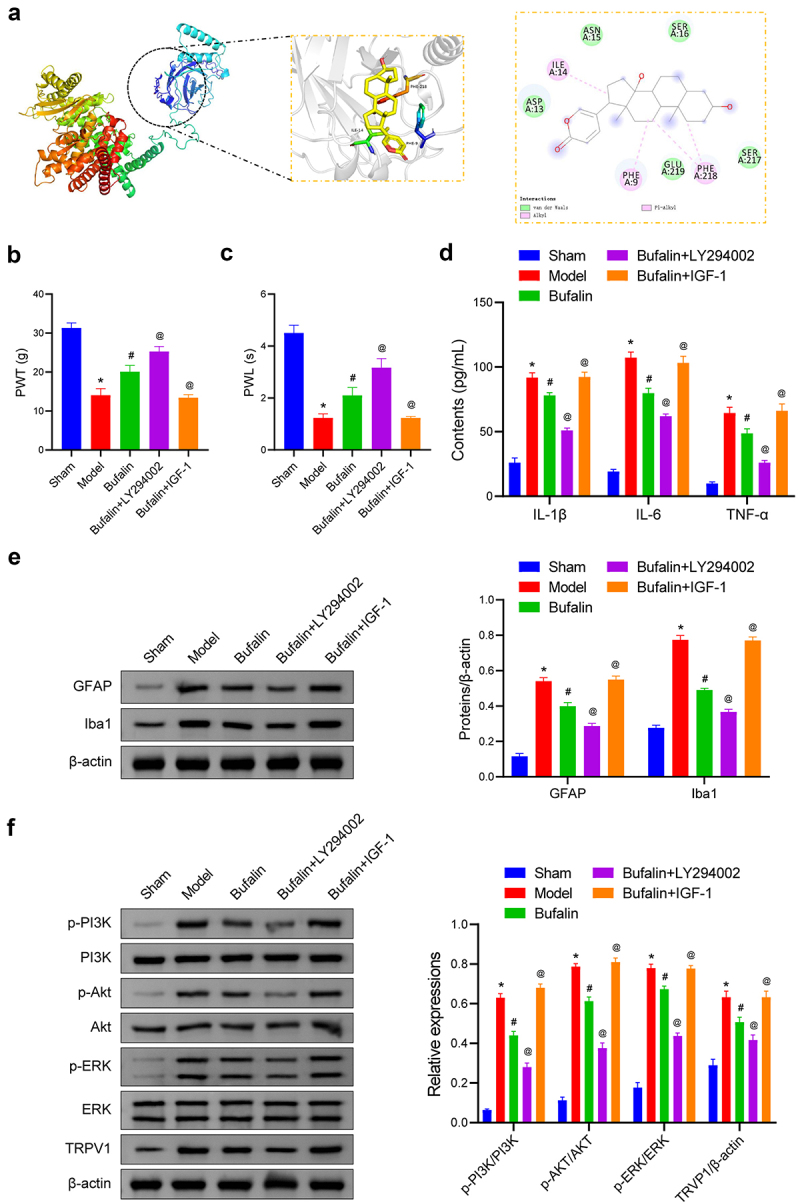
The effects of Bufalin on the BCP rat model. (a) The binding interactions between certain compounds and the binding site of the hub genes protein. (b-c) PWT and PWL measurements were conducted in BCP rats. (d) The concentrations of IL-1β, IL-6, and TNF-α in rat serum were measured. (e) The expression of GFAP and Iba1 was examined in the spinal dorsal horn of rats. The original blots are presented in supplementary figure S1. (f) The levels of p-PI3K/PI3K, p-Akt/Akt, p-ERK/ERK, and TRVP1 were examined. The original blots are presented in supplementary figure S1. *n*=6 per group. *p < .05 vs. Sham. ^#^*p* < .05 vs. Model. ^@^*p* < .05 vs. Bufalin.

Based on the UPLC-MS analysis findings, Bufalin and Muscone were identified as the primary active ingredients of LSW. Notably, Bufalin has demonstrated potential in mitigating cancer-induced pain and bone destruction in a bone cancer model.^32^ Consequently, it was hypothesized that LSW could potentially offer therapeutic benefits for rats with BCP through the action of Bufalin. To explore this further, molecular docking studies were conducted on Bufalin using the RCSB Protein Data Bank database targeting PI3K. The docking simulations revealed various binding modes such as van der Waals, alkyl, and pi-alkyl interactions (Figure 7a). The binding energy between Bufalin and PI3K was −6.9 kcal/mol, indicating a substantial affinity between Bufalin and PI3K. Subsequent investigations on Bufalin’s impact on BCP in rats showed significant improvements in pain thresholds and reduced inflammatory cytokine levels, suggesting analgesic and anti-inflammatory effects (Figures 7b–d). Additionally, Bufalin was found to modulate the activation of astrocytes and microglia in BCP rats (Figure 7e). These effects were further enhanced by LY294002 and reversed by IGF-1, indicating the involvement of the PI3K/Akt and TRPV1 signaling pathways in mediating Bufalin’s therapeutic effects on BCP rats.

## 4. Discussion

In various prevalent types of cancer such as breast, prostate, kidney, and lung cancers, tumors exhibit a notable propensity to spread to specific skeletal sites, including vertebrae, ribs, hip, femur, and tibia.^33^ Recent research indicates that targeting exosome-derived RNA could be a promising therapeutic approach.^34–37^ The frequent metastasis of tumors to bone can result in various complications such as pain, hypercalcemia, anemia, increased infection susceptibility, bone fractures, spinal cord compression, spinal instability, and mobility issues.^38^ Studies suggest that a significant proportion of patients with metastatic cancer pain experience inadequate relief from existing pharmacological interventions.^39,40^ Noteworthy, studies have demonstrated that endogenous cannabinoids can alleviate pain in murine bone cancer models without the adverse effects associated with opioids.^41^ The optimal treatment strategy currently sought is one that effectively addresses primary cancer while concurrently managing cancer-related pain, thereby addressing both the pain symptoms and the underlying disease.

TCM has a long-established history of utilizing pharmacological approaches for the treatment of BCP. Zhou *et al*. identified luteolin as a potential agent for alleviating BCP associated with lung cancer by targeting NLRP3 inflammasome and glial activation.^42^ Furthermore, the total alkaloids of *Corydalis* have been found to inhibit the RANKl-induced NF-κB and c-Fos/NFATc1 pathway, thereby mitigating the progression of BCP.^43^ In a similar vein, the current investigation demonstrated that LSW significantly elevated the pain threshold in rats, as indicated by enhanced PWT and PWL, while also reducing levels of inflammatory cytokines in the serum. These findings suggest that LSW effectively mitigated cancer-related pain and inflammation in BCP rats. Additionally, network pharmacology analysis revealed 275 shared targets between LSW and BCP, providing evidence of the close interactions between these entities.

In the majority of pain conditions, there are various mechanisms of pain signaling present in the vicinity of the affected area. One such mechanism is the PI3K/Akt pathway, which is known for its regulation of diverse biological processes such as cell proliferation, apoptosis, and tissue inflammation. This pathway plays a crucial role in the development of neuropathic pain, inflammatory mechanical pain, and cancer-related pain.^44,45^ Aberrant activation of the PI3K/Akt signaling is commonly observed in cancer-induced pain.^46^ In the BCP model, the analgesic effects were noted through the inhibition of the PI3K/Akt/WNK1 pathway.^12^ Furthermore, inhibiting the PI3K/Akt/mTOR pathway in the periaqueductal gray region resulted in a reduction in hyperalgesic responses in BCP rats.^47^ Our research revealed a consistent increase in PI3K/Akt signaling in BCP rats. Treatment with LY294002 was found to enhance the efficacy of LSW, whereas the application of IGF-1 counteracted the effects of LSW. These findings indicate that LSW treatment exerts its therapeutic effects through the inhibition of the PI3K/Akt signaling pathway. However, Li *et al*. reported a decrease in PI3K/Akt expression during the progression of postoperative chronic pain. Microglia are implicated in promoting the transformation of astrocytes into the A1 phenotype through the downregulation of PI3K/Akt activation, thereby exacerbating postoperative chronic pain.^48^

The activation of ERK and the regulation of TRPV1 activity in relation to PI3K have been examined.^49^ TRPV1, a nociceptor present in peripheral nerve fibers, plays a crucial role in the advancement of cancer-related pain.^50^ The involvement of TRPV1 in the development of neuropathic pain in rats with bone cancer has been previously confirmed.^23^ Moreover, it has been observed that nociception can arise in a model of mammary gland tumor cell inoculation in mice with bone metastasis, contingent upon the activation of TRPV1.^51^ PD-L1/PD-1 signaling has also been identified as inhibiting BCP by suppressing TRPV1 activity.^52^ In the present study, it has been observed that LSW demonstrates a downregulating effect on TRPV1 expression. Concurrently, the administration of LY294002 resulted in a further decrease in TRPV1 expression, whereas the application of IGF-1 reversed this effect. These findings suggest that LSW possesses therapeutic attributes by attenuating TRPV1 signaling through the PI3K/Akt pathway.

To identify the active ingredients responsible for the therapeutic effects of LSW on BCP, a UPLC-MS analysis was conducted on both LSW and serum containing LSW. Bufalin and Muscone were identified as unique ingredients present in the LSW and LSW-containing serum, which were absent in the serum of rats not treated with LSW. Bufalin is a naturally occurring small molecule known for its anti-inflammatory properties, showing effectiveness in alleviating cancer-induced pain and mitigating bone destruction in a murine bone cancer model.^32^ It has been recognized as a key regulator of bone cancer cell growth and apoptosis.^53^ Besides, the administration of Bufalin has demonstrated anti-inflammatory and analgesic effects in rodent models.^54^ Muscone, another active ingredient, is utilized in the treatment of various conditions such as neurological disorders, chronic inflammation, and ischemia-reperfusion injury.^55^ Muscone has been found to alleviate inflammatory pain by inhibiting microglial activation-mediated inflammatory responses through the NOX4/JAK2-STAT3 pathway and NLRP3 inflammasome.^56^ Given that Bufalin is the most abundant of the components that fulfill the condition of being a unique component of LSW and LSW-containing serum, we hypothesized that LSW might play a therapeutic role in BCP rats through the Bufalin component and performed molecular docking studies of Bufalin and PI3K. Our results revealed that Bufalin exerted analgesic and anti-inflammatory effects by PI3K/Akt and TRPV1 signaling pathways in BCP rats. The use of LSW thus holds promise in alleviating BCP by leveraging these specific ingredients, particularly Bufalin, offering a novel therapeutic strategy for BCP management.

In our study, various methods of administration such as oral, intrathecal, and intraperitoneal were employed. However, a standardized intrathecal injection approach is recommended for assessing central effects, which stands as a notable limitation of our study. Additionally, apart from Bufalin, other active components within LSW may also contribute significantly to its therapeutic efficacy, although their impact was not confirmed in this study, suggesting a potential avenue for future research. Furthermore, our study specifically addressed the effects of LSW on inflammation and nerve pain in BCP rats, yet the osteolytic lesions triggered by metastatic tumor cells were not investigated, indicating a limitation within the scope of our research. Moving forward, our focus will be directed toward exploring the role of LSW in addressing BCP-related osteolytic lesions to enhance the development of treatment strategies for BCP.

## 5. Conclusion

In summary, our study has established that LSW exhibits favorable bioavailability in BCP rats. This bioavailability effectively inhibits the malignant characteristics of Walker 256 cells *in vitro*, as well as alleviates BCP-related inflammatory pain *in vivo*. Furthermore, both the administration of LSW and its active ingredient Bufalin demonstrated inhibitory effects on the PI3K/Akt and TRPV1 signaling pathways during BCP treatment. These findings present promising new avenues for addressing BCP through innovative therapeutic strategies.

## Supplementary Material

Supplementary Figure S1.jpg

Supplementary Materials.docx

## Data Availability

If necessary, all raw data can be obtained from the corresponding author.

## Abbreviations

BCP, bone cancer pain; LSW, Liu-Shen-Wan; UPLC-MS, ultra-performance liquid chromatography-mass spectrometry; KEGG, Kyoto Encyclopedia of Genes and Genomes; SD, Sprague Dawley; PWT, paw withdrawal threshold; PWL, paw withdrawal latency; FBS, fetal bovine serum; OD, optical density; ELISA, enzyme-linked immunosorbent assay; IHC, immunocytochemistry; DAB, diaminobenzidine; qRT-PCR, quantitative reverse-transcription polymerase chain reaction; TRPV1, transient receptor potential vanillin subfamily member 1; BCA, bicinchoninic acid assay; ANOVA, analysis of variance.
