# Supplementary figures and images for: Liu-Shen-Wan inhibits PI3K/Akt and TRPV1 signaling alleviating bone cancer pain in rats
Source: Cancer Biol Ther. 2024 Nov 25;25(1):2432098. doi: 10.1080/15384047.2024.2432098 (PMC11601056; doi:10.1080/15384047.2024.2432098)

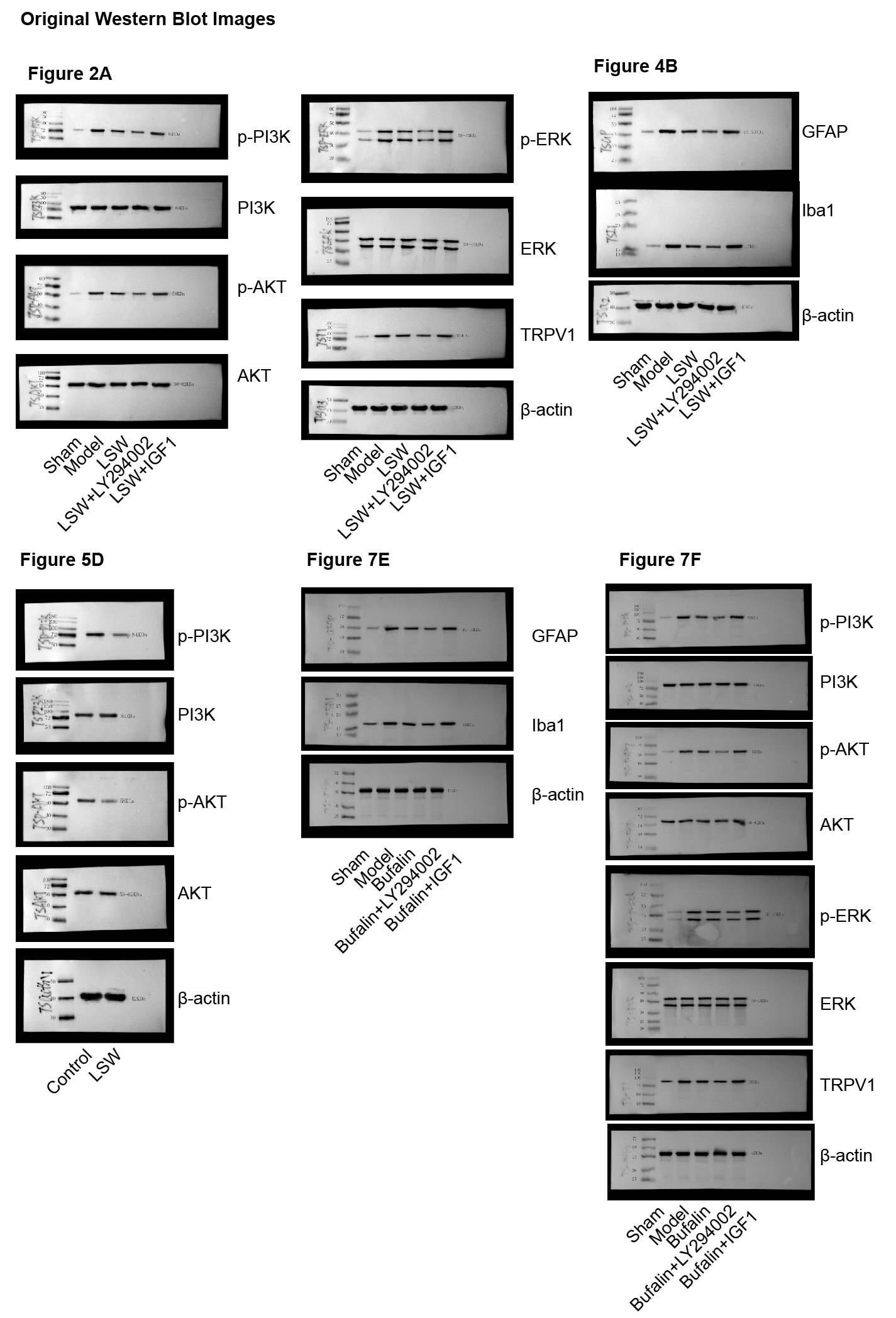

Supplement: Supplementary Figure S1.jpg [file KCBT_A_2432098_SM1978.jpg]
