# Supplementary material for: Liu-Shen-Wan inhibits PI3K/Akt and TRPV1 signaling alleviating bone cancer pain in rats
Source: Cancer Biol Ther. 2024 Nov 25;25(1):2432098. doi: 10.1080/15384047.2024.2432098 (PMC11601056; doi:10.1080/15384047.2024.2432098)
Supplement: Supplementary Materials.docx [file KCBT_A_2432098_SM1977.docx]

**Table S1. Quantification of drug-containing serum in the Control group (NEG mode)**

| **Area** | **Height** | **Retention Time** | **Adduct / Charge** | **Found At Mass** | **Library Hit** |
| --- | --- | --- | --- | --- | --- |
| 20,640 | 6,495 | 7.16 | [M-H]- | 165.0558 | (R)-2-Hydroxy-3-phenylpropionic acid |
| 900,200 | 66,160 | 2.52 | [M-H]- | 103.0401 | (R)-3-Hydroxybutyric acid |
| 6,429 | 810 | 5.48 | [M-H]- | 131.071 | (S)-(-)-2-Hydroxyisocaproic Acid |
| 446,400 | 127,900 | 10.43 | [M-H]- | 243.16 | 1,11-Undecanedicarboxylic acid |
| 8,436 | 1,872 | 10.68 | [M-H]- | 187.1342 | 10-HYDROXYDECANOATE |
| 24,040 | 8,610 | 15.55 | [M-H]- | 329.2136 | 11a-Hydroxyprogesterone |
| 1,121,000 | 329,400 | 13.36 | [M-H]- | 319.2271 | 12(S)HETE |
| 28,290 | 9,468 | 13.88 | [M-H]- | 319.2272 | 15(S)HETE |
| 12,350 | 3,114 | 4.77 | [M-H]- | 181.0367 | 1-Methyluric Acid |
| 4,729 | 356 | 5.98 | [M-H]- | 145.05 | 2,2-Dimethylsuccinic acid |
| 1,244 | 139 | 1.57 | [M-H]- | 99.0459 | 2,3-Pentanedione |
| 9,175 | 1,300 | 0.97 | [M-H]- | 163.0254 | 2,4-DIHYDROXYPTERIDINE |
| 9,020 | 2,531 | 13.78 | [M-H]- | 205.1598 | 2,4-Di-tert-butylphenol |
| 6,406 | 677 | 10.59 | [M-H]- | 219.1751 | 2,6-Di-tert-butyl-4-methylphenol |
| 9,537 | 1,214 | 15.33 | [M-H]- | 219.1753 | 2,6-Di-tert-butyl-4-methylphenol |
| 9,793 | 1,224 | 13.2 | [M-H]- | 219.1753 | 2,6-Di-tert-butyl-4-methylphenol |
| 8,665 | 2,177 | 5.61 | [M-H]- | 198.0328 | 2-Chloro-L-phenylalanine |
| 1,223 | 228 | 8.46 | [M-H]- | 168.0304 | 2-Furoylglycine |
| 895,600 | 65,720 | 2.52 | [M-H]- | 103.0401 | 2-Hydroxyisobutyric acid |
| 10,990 | 1,439 | 7.44 | [M-H]- | 131.0713 | 2-Hydroxyisocaproic Acid |
| 7,951 | 2,085 | 8.97 | [M-H]- | 159.1026 | 2-Hydroxyoctanoic acid |
| 43,560 | 6,535 | 5.47 | [M-H]- | 145.0506 | 2-METHYLGLUTARATE |
| 4,729 | 356 | 5.98 | [M-H]- | 145.05 | 2-METHYLGLUTARATE |
| 43,560 | 6,535 | 5.47 | [M-H]- | 145.0506 | 2-Methylglutaric acid |
| 4,729 | 356 | 5.98 | [M-H]- | 145.05 | 2-Methylglutaric acid |
| 4,880 | 616 | 2.36 | [M-H]- | 129.0194 | 2-METHYLMALEATE |
| 6,556 | 1,549 | 4.85 | [M-H]- | 131.0348 | 2-Methylsuccinic Acid |
| 10,850 | 3,164 | 6.87 | [M-H]- | 157.0866 | 2-Oxooctanoic acid |
| 1,697 | 286 | 9.76 | [M-H]- | 165.0562 | 3-(3-Hydroxyphenyl)Propionate Acid |
| 19,700 | 2,480 | 7.17 | [M-H]- | 179.0349 | 3-(4-HYDROXYPHENYL)PYRUVATE |
| 1,631 | 260 | 5 | [M-H]- | 179.0353 | 3-(4-HYDROXYPHENYL)PYRUVATE |
| 5,209 | 624 | 9.42 | [M-H]- | 226.9918 | 3,5-Dinitrosalicylate |
| 5,209 | 624 | 9.42 | [M-H]- | 226.9918 | 3,5-Dinitrosalicylic acid |
| 39,910 | 11,590 | 1.04 | [M-H]- | 130.0623 | 3-Guanidinopropionic acid |
| 5,018 | 301 | 13.81 | [M-H]- | 121.0292 | 3-HYDROXYBENZALDEHYDE |
| 1,851 | 262 | 16.5 | [M-H]- | 121.0292 | 3-HYDROXYBENZALDEHYDE |
| 6,580 | 1,183 | 8.07 | [M-H]- | 137.0246 | 3-Hydroxybenzoic acid |
| 900,200 | 66,160 | 2.52 | [M-H]- | 103.0401 | 3-HYDROXYBUTANOATE |
| 691,000 | 78,440 | 1.68 | [M-H]- | 103.04 | 3-HYDROXYBUTANOATE |
| 900,200 | 66,160 | 2.52 | [M-H]- | 103.0401 | 3-Hydroxybutyric acid |
| 26,210 | 2,968 | 1.59 | [M-H]- | 161.0455 | 3-HYDROXYMETHYLGLUTARATE |
| 15,190 | 1,694 | 3 | [M-H]- | 161.0456 | 3-HYDROXYMETHYLGLUTARATE |
| 7,181 | 1,910 | 6.25 | [M-H]- | 151.0401 | 3-HYDROXYPHENYLACETATE |
| 6,874 | 1,385 | 5.56 | [M-H]- | 202.0822 | 3-Indolebutyric acid |
| 278,900 | 87,890 | 8.72 | [M-H]- | 188.0717 | 3-Indolepropionic acid |
| 54,500 | 11,180 | 6.26 | [M-H]- | 159.0662 | 3-Methyladipic acid |
| 38,700 | 5,236 | 5.73 | [M-H]- | 156.0666 | 3-Methylcrotonyl Glycine |
| 3,592 | 641 | 7.33 | [M-H]- | 225.0423 | 3-NITRO-L-TYROSINE |
| 3,592 | 641 | 7.33 | [M-H]- | 225.0423 | 3-Nitrotyrosine |
| 20,600 | 3,234 | 9.69 | [M-H]- | 405.2631 | 3-Oxocholic acid |
| 2,069 | 606 | 1.03 | [M-H]- | 131.0655 | 4-Aminoindole |
| 1,851 | 262 | 16.5 | [M-H]- | 121.0292 | 4-HYDROXYBENZALDEHYDE |
| 6,580 | 1,183 | 8.07 | [M-H]- | 137.0246 | 4-HYDROXYBENZOATE |
| 6,580 | 1,183 | 8.07 | [M-H]- | 137.0246 | 4-Hydroxybenzoic acid |
| 4,426 | 867 | 10.18 | [M-H]- | 166.0506 | 4-HYDROXY-L-PHENYLGLYCINE |
| 4,687 | 732 | 5.95 | [M-H]- | 144.0457 | 4-Hydroxyquinoline |
| 3,089 | 926 | 7.8 | [M-H]- | 144.0454 | 4-Hydroxyquinoline |
| 27,060 | 3,433 | 8.2 | [M-H]- | 138.0197 | 4-Nitrophenol |
| 3,661 | 548 | 14.97 | [M-H]- | 138.0192 | 4-Nitrophenol |
| 3,662 | 697 | 4.46 | [M-H]- | 182.0461 | 4-PYRIDOXATE |
| 6,409 | 455 | 2.89 | [M-H]- | 182.0455 | 4-PYRIDOXATE |
| 2,069 | 606 | 1.03 | [M-H]- | 131.0655 | 5-Aminoindole |
| 54,790 | 15,380 | 6.89 | [M-H]- | 190.051 | 5-HYDROXYINDOLEACETATE |
| 35,560 | 6,674 | 4.73 | [M-H]- | 190.051 | 5-HYDROXYINDOLEACETATE |
| 10,180 | 2,793 | 6.16 | [M-H]- | 190.0508 | 5-HYDROXYINDOLEACETATE |
| 4,880 | 616 | 2.36 | [M-H]- | 129.0194 | 5-Oxo-2-tetrahydrofurancarboxylic acid |
| 45,040 | 13,190 | 1.31 | [M-H]- | 130.0873 | 6-Aminocaproic Acid |
| 54,500 | 11,180 | 6.26 | [M-H]- | 159.0662 | 6-CARBOXYHEXANOATE |
| 36,510 | 11,100 | 11.14 | [M-H]- | 389.2679 | 7a-Hydroxy-3-oxo-5b-cholanoic acid |
| 29,730 | 8,948 | 12.3 | [M-H]- | 389.2682 | 7a-Hydroxy-3-oxo-5b-cholanoic acid |
| 14,700 | 2,418 | 11.51 | [M-H]- | 389.2686 | 7a-Hydroxy-3-oxo-5b-cholanoic acid |
| 8,034 | 2,110 | 8.97 | [M-H]- | 159.1026 | 8-Hydroxyoctanoic acid |
| 13,140 | 3,223 | 4.77 | [M-H]- | 181.0367 | 9-Methyluric acid |
| 43,560 | 6,535 | 5.47 | [M-H]- | 145.0506 | ADIPATE |
| 4,729 | 356 | 5.98 | [M-H]- | 145.05 | ADIPATE |
| 4,409 | 366 | 15.89 | [M-H]- | 297.0452 | Aflatoxin P1 |
| 2,902 | 456 | 13.19 | [M-H]- | 297.0466 | Aflatoxin P1 |
| 2,902 | 456 | 13.19 | [M-H]- | 297.0466 | Aflatoxin P1 |
| 188,200 | 61,220 | 1 | [M-H]- | 157.0368 | ALLANTOIN |
| 45,040 | 13,190 | 1.31 | [M-H]- | 130.0873 | Alloisoleucine |
| 682,000 | 146,300 | 14.89 | [M-H]- | 277.2172 | Alpha-Linolenic acid |
| 45,040 | 13,190 | 1.31 | [M-H]- | 130.0873 | Aminocaproic acid |
| 18,050 | 3,783 | 18.74 | [M-H]- | 311.2953 | ARACHIDATE |
| 5,749 | 1,366 | 17.39 | [M-H]- | 311.2862 | ARACHIDATE |
| 18,050 | 3,783 | 18.74 | [M-H]- | 311.2953 | Arachidic acid |
| 5,749 | 1,366 | 17.39 | [M-H]- | 311.2862 | Arachidic acid |
| 81,180 | 23,250 | 1.25 | [M-H]- | 175.0248 | ASCORBATE |
| 1,697 | 286 | 9.76 | [M-H]- | 165.0562 | Atrolactic acid |
| 891,200 | 256,300 | 7.76 | [M-H]- | 187.0977 | Azelaic acid |
| 891,200 | 256,300 | 7.76 | [M-H]- | 187.0977 | AZELATE |
| 5,018 | 301 | 13.81 | [M-H]- | 121.0292 | BENZOATE |
| 1,851 | 262 | 16.5 | [M-H]- | 121.0292 | BENZOATE |
| 59,170 | 19,230 | 1.04 | [M-H]- | 88.0405 | BETA-ALANINE |
| 45,040 | 13,190 | 1.31 | [M-H]- | 130.0873 | Beta-Leucine |
| 66,960 | 11,730 | 17.7 | [M-H]- | 583.2549 | Bilirubin |
| 12,610 | 3,242 | 10.65 | [M-H]- | 581.2402 | BILIVERDIN |
| 6,324 | 938 | 14.72 | [M-H]- | 157.1229 | Butyl 3-methylbutanoate |
| 6,324 | 938 | 14.72 | [M-H]- | 157.1229 | Butyl 3-methylbutanoate |
| 1,840 | 406 | 16.25 | [M-H]- | 115.0767 | Butyl Acetate |
| 4,082 | 648 | 16.36 | [M-H]- | 115.0751 | Butyl Acetate |
| 19,700 | 2,480 | 7.17 | [M-H]- | 179.0349 | CAFFEATE |
| 22,490 | 7,311 | 12.33 | [M-H]- | 171.1389 | Capric acid |
| 3,686 | 350 | 16.5 | [M-H]- | 143.1073 | CAPRYLATE |
| 416,400 | 106,900 | 10.88 | [M-H]- | 391.284 | Chenodeoxycholic acid |
| 357,400 | 103,000 | 12.25 | [M-H]- | 391.284 | Chenodeoxycholic acid |
| 48,420 | 10,750 | 11.32 | [M-H]- | 391.2842 | Chenodeoxycholic acid |
| 901 | 248 | 9.64 | [M-H]- | 391.2754 | Chenodeoxycholic acid |
| 868,500 | 219,200 | 10.63 | [M-H]- | 407.2793 | Cholic acid |
| 124,700 | 35,060 | 9.71 | [M-H]- | 407.2792 | Cholic acid |
| 26,960 | 7,941 | 9.31 | [M-H]- | 407.2785 | Cholic acid |
| 66,670 | 12,170 | 4.82 | [M-H]- | 147.045 | Cinnamic acid |
| 94,500 | 14,930 | 1.33 | [M-H]- | 173.0092 | cis-Aconitic acid |
| 16,150 | 1,999 | 2.37 | [M-H]- | 173.0092 | cis-Aconitic acid |
| 16,150 | 1,999 | 2.37 | [M-H]- | 173.0092 | cis-Aconitic acid |
| 4,880 | 616 | 2.36 | [M-H]- | 129.0194 | Citraconic acid |
| 764,500 | 245,800 | 1.25 | [M-H]- | 191.0199 | CITRATE |
| 135,600 | 15,360 | 1.9 | [M-H]- | 191.0198 | CITRATE |
| 744,300 | 239,300 | 1.25 | [M-H]- | 191.0199 | CITRATE |
| 132,400 | 14,940 | 1.9 | [M-H]- | 191.0198 | CITRATE |
| 764,500 | 245,800 | 1.25 | [M-H]- | 191.0199 | Citric acid |
| 135,600 | 15,360 | 1.9 | [M-H]- | 191.0198 | Citric acid |
| 4,620 | 1,003 | 10.37 | [M-H]- | 249.0766 | Citrinin |
| 177,600 | 53,420 | 0.96 | [M-H]- | 174.0885 | Citrulline |
| 295,700 | 98,210 | 16.13 | [M-H]- | 331.264 | CP 47,497 C8-homolog Negative Mode |
| 39,910 | 11,590 | 1.04 | [M-H]- | 130.0623 | CREATINE |
| 59,170 | 19,230 | 1.04 | [M-H]- | 88.0405 | D-Alanine |
| 357,400 | 103,000 | 12.25 | [M-H]- | 391.284 | DEOXYCHOLATE |
| 416,400 | 106,900 | 10.88 | [M-H]- | 391.284 | Deoxycholic acid |
| 357,400 | 103,000 | 12.25 | [M-H]- | 391.284 | Deoxycholic acid |
| 48,420 | 10,750 | 11.32 | [M-H]- | 391.2842 | Deoxycholic acid |
| 901 | 248 | 9.64 | [M-H]- | 391.2754 | Deoxycholic acid |
| 28,670 | 4,336 | 1.85 | [M-H]- | 226.0835 | DEOXYCYTIDINE |
| 58,590 | 8,019 | 1.5 | [M-H]- | 117.0193 | D-Erythronolactone |
| 41,940 | 4,514 | 2.37 | [M-H]- | 117.0193 | D-Erythronolactone |
| 74,970 | 20,640 | 0.91 | [M-H]- | 179.056 | D-Fructose |
| 74,970 | 20,640 | 0.91 | [M-H]- | 179.056 | D-Fructose |
| 73,410 | 20,110 | 0.91 | [M-H]- | 179.056 | D-Fructose |
| 6,699 | 1,589 | 4.85 | [M-H]- | 131.0348 | Dimethylmalonic acid |
| 8,639 | 682 | 6.76 | [M-H]- | 89.0241 | D-Lactic acid |
| 8,639 | 682 | 6.76 | [M-H]- | 89.0241 | D-Lactic acid |
| 1,330,000 | 105,100 | 1.49 | [M-H]- | 89.0244 | DL-Glyceraldehyde |
| 8,440 | 673 | 6.77 | [M-H]- | 89.0244 | DL-Glyceraldehyde |
| 8,440 | 673 | 6.77 | [M-H]- | 89.0244 | DL-Glyceraldehyde |
| 158,000 | 48,460 | 9.8 | [M-H]- | 229.1445 | Dodecanedioic acid |
| 158,000 | 48,460 | 9.8 | [M-H]- | 229.1445 | Dodecanedioic acid |
| 158,000 | 48,460 | 9.8 | [M-H]- | 229.1445 | Dodecanedioic acid |
| 152,100 | 46,670 | 9.8 | [M-H]- | 229.1445 | Dodecanedioic acid |
| 185,100 | 62,320 | 13.77 | [M-H]- | 199.1705 | Dodecanoic acid |
| 45,020 | 13,440 | 0.96 | [M-H]- | 131.0823 | D-ORNITHINE |
| 74,970 | 20,640 | 0.91 | [M-H]- | 179.056 | D-Tagatose |
| 3,958 | 394 | 13.77 | [M-H]- | 165.0406 | D-Xylonic Acid Lithium Salt |
| 73,410 | 20,110 | 0.91 | [M-H]- | 179.056 | D-Galactose |
| 73,410 | 20,110 | 0.91 | [M-H]- | 179.056 | D-(+)-Glucose |
| 73,410 | 20,110 | 0.91 | [M-H]- | 179.056 | D-(+)-Mannose |
| 2,481,000 | 648,300 | 16.3 | [M-H]- | 281.2485 | Elaidic acid |
| 3,112 | 301 | 17.48 | [M-H]- | 281.2477 | Elaidic acid |
| 2,481,000 | 648,300 | 16.3 | [M-H]- | 281.2485 | Elaidic acid |
| 3,112 | 301 | 17.48 | [M-H]- | 281.2477 | Elaidic acid |
| 2,350,000 | 613,800 | 16.3 | [M-H]- | 281.2485 | Elaidic acid |
| 2,545 | 285 | 17.47 | [M-H]- | 281.2477 | Elaidic acid |
| 1,535 | 149 | 7.83 | [M-H]- | 269.0466 | Emodin |
| 23,440 | 4,769 | 18.81 | [M-H]- | 337.311 | Erucic acid |
| 266,700 | 84,510 | 15.05 | [M-H]- | 227.2016 | Ethyl dodecanoate |
| 798,000 | 189,200 | 17.3 | [M-H]- | 283.2643 | Ethyl hexadecanoate1 |
| 6,699 | 1,589 | 4.85 | [M-H]- | 131.0348 | ETHYLMALONATE |
| 19,430 | 6,161 | 9.03 | [M-H]- | 165.0557 | Ethylparaben |
| 2,294,000 | 640,700 | 15.39 | [M-H]- | 303.2328 | Fatty acid C20:4 |
| 6,507 | 1,204 | 6.13 | [M-H]- | 455.0941 | Flavin Mononucleotide |
| 6,618 | 921 | 9.81 | [M-H]- | 341.0033 | FPePA (3-Perfluoropentyl propanoic acid) (neg) |
| 4,711 | 676 | 9.64 | [M-H]- | 341.0034 | FPePA (3-Perfluoropentyl propanoic acid) (neg) |
| 5,367 | 636 | 2.36 | [M-H]- | 129.0194 | Fumaric acid monomethyl ester |
| 5,490 | 1,283 | 15.39 | [M-H]- | 297.1558 | Fumigaclavine A |
| 91,100 | 30,120 | 0.92 | [M-H]- | 145.0617 | GLUTAMINE |
| 6,699 | 1,589 | 4.85 | [M-H]- | 131.0348 | GLUTARATE |
| 6,699 | 1,589 | 4.85 | [M-H]- | 131.0348 | Glutaric acid |
| 1,334,000 | 105,600 | 1.49 | [M-H]- | 89.0244 | GLYCERALDEHYDE |
| 5,175 | 631 | 7.25 | [M-H]- | 89.0242 | GLYCERALDEHYDE |
| 8,639 | 682 | 6.76 | [M-H]- | 89.0241 | GLYCERALDEHYDE |
| 8,639 | 682 | 6.76 | [M-H]- | 89.0241 | GLYCERALDEHYDE |
| 35,760 | 10,400 | 1.05 | [M-H]- | 105.0192 | GLYCERATE |
| 35,760 | 10,400 | 1.05 | [M-H]- | 105.0192 | Glyceric acid |
| 95,750 | 30,870 | 9.75 | [M-H]- | 448.3057 | GLYCOCHENODEOXYCHOLATE |
| 41,790 | 12,380 | 10.98 | [M-H]- | 448.3053 | GLYCOCHENODEOXYCHOLATE |
| 187,900 | 55,950 | 9.67 | [M-H]- | 464.3008 | GLYCOCHOLATE |
| 32,780 | 6,226 | 9.04 | [M-H]- | 464.301 | GLYCOCHOLATE |
| 187,900 | 55,950 | 9.67 | [M-H]- | 464.3008 | Glycocholic acid |
| 32,780 | 6,226 | 9.04 | [M-H]- | 464.301 | Glycocholic acid |
| 98,160 | 30,030 | 16.69 | [M-H]- | 269.2484 | HEPTADECANOATE |
| 98,160 | 30,030 | 16.69 | [M-H]- | 269.2484 | Heptadecanoic acid |
| 73,280 | 22,670 | 12.24 | [M-H]- | 285.2064 | Hexadecanedioic acid |
| 9,048 | 1,607 | 11.63 | [M-H]- | 285.2062 | Hexadecanedioic acid |
| 1,667 | 508 | 15.62 | [M-H]- | 241.2584 | HEXADECANOL |
| 137,500 | 36,000 | 6.34 | [M-H]- | 178.0509 | HIPPURATE |
| 137,500 | 36,000 | 6.34 | [M-H]- | 178.0509 | Hippuric acid |
| 24,220 | 7,126 | 0.9 | [M-H]- | 154.0619 | HISTIDINE |
| 12,600 | 3,702 | 1.11 | [M-H]- | 188.1041 | Homocitrulline |
| 107,000 | 32,230 | 5.69 | [M-H]- | 181.0506 | HYDROXYPHENYLLACTATE |
| 107,000 | 32,230 | 5.69 | [M-H]- | 181.0506 | Hydroxyphenyllactic acid |
| 416,400 | 106,900 | 10.88 | [M-H]- | 391.284 | Hyodeoxycholic Acid |
| 357,400 | 103,000 | 12.25 | [M-H]- | 391.284 | Hyodeoxycholic Acid |
| 48,420 | 10,750 | 11.32 | [M-H]- | 391.2842 | Hyodeoxycholic Acid |
| 901 | 248 | 9.64 | [M-H]- | 391.2754 | Hyodeoxycholic Acid |
| 416,400 | 106,900 | 10.88 | [M-H]- | 391.284 | Hyodeoxycholic Acid |
| 357,400 | 103,000 | 12.25 | [M-H]- | 391.284 | Hyodeoxycholic Acid |
| 48,420 | 10,750 | 11.32 | [M-H]- | 391.2842 | Hyodeoxycholic Acid |
| 901 | 248 | 9.64 | [M-H]- | 391.2754 | Hyodeoxycholic Acid |
| 369,900 | 95,220 | 10.88 | [M-H]- | 391.284 | Hyodeoxycholic Acid |
| 324,100 | 93,590 | 12.25 | [M-H]- | 391.284 | Hyodeoxycholic Acid |
| 43,040 | 9,586 | 11.32 | [M-H]- | 391.2842 | Hyodeoxycholic Acid |
| 5,531 | 1,367 | 7.63 | [M-H]- | 160.0402 | Indole-3-carboxylic acid |
| 4,169 | 1,227 | 7.63 | [M-H]- | 160.0402 | Indole-5-Carboxylic Acid |
| 90,140 | 27,920 | 8.46 | [M-H]- | 186.0561 | Indoleacrylic acid |
| 52,850 | 15,590 | 7.4 | [M-H]- | 204.0664 | Indolelactic acid |
| 52,850 | 15,590 | 7.4 | [M-H]- | 204.0664 | Indolelactic acid |
| 51,530 | 15,210 | 7.4 | [M-H]- | 204.0664 | Indolelactic acid |
| 1,436,000 | 302,900 | 6.31 | [M-H]- | 212.0024 | INDOXYL SULFATE |
| 13,020 | 1,527 | 5.27 | [M-H]- | 212.0023 | INDOXYL SULFATE |
| 6,217 | 1,633 | 5.97 | [M-H]- | 87.0454 | ISOBUTYRATE |
| 45,040 | 13,190 | 1.31 | [M-H]- | 130.0873 | ISOLEUCINE |
| 92,930 | 28,450 | 16.69 | [M-H]- | 269.2484 | Isopropyl myristate |
| 88,200 | 14,500 | 5.85 | [M-H]- | 158.0822 | Isovalerylglycine |
| 4,880 | 616 | 2.36 | [M-H]- | 129.0194 | ITACONATE |
| 4,880 | 616 | 2.36 | [M-H]- | 129.0194 | Itaconic acid |
| 865,200 | 109,600 | 6.33 | [M-H]- | 129.0557 | KETOLEUCINE |
| 13,180 | 2,299 | 5.96 | [M-H]- | 188.0355 | KYNURENATE |
| 2,184 | 638 | 8.71 | [M-H]- | 188.0418 | KYNURENATE |
| 43,700 | 13,200 | 11.64 | [M-H]- | 194.0822 | L- tyrosine methyl ester |
| 21,570 | 6,668 | 7.16 | [M-H]- | 165.0558 | L-3-Phenyllactic acid |
| 1,334,000 | 105,600 | 1.49 | [M-H]- | 89.0244 | LACTATE |
| 5,175 | 631 | 7.25 | [M-H]- | 89.0242 | LACTATE |
| 8,639 | 682 | 6.76 | [M-H]- | 89.0241 | LACTATE |
| 8,639 | 682 | 6.76 | [M-H]- | 89.0241 | LACTATE |
| 59,170 | 19,230 | 1.04 | [M-H]- | 88.0405 | L-ALANINE |
| 45,040 | 13,190 | 1.31 | [M-H]- | 130.0873 | L-Alloisoleucine |
| 185,100 | 62,320 | 13.77 | [M-H]- | 199.1705 | LAURATE |
| 45,040 | 13,190 | 1.31 | [M-H]- | 130.0873 | LEUCINE |
| 2,409 | 497 | 15.43 | [M-H]- | 233.158 | Lidocaine |
| 4,297,000 | 1,286,000 | 15.55 | [M-H]- | 279.2328 | Linoleic acid |
| 4,141 | 669 | 16.33 | [M-H]- | 279.2305 | Linoleic acid |
| 45,040 | 13,190 | 1.31 | [M-H]- | 130.0873 | L-Isoleucine |
| 7,469 | 1,947 | 13.95 | [M-H]- | 375.2887 | LITHOCHOLATE |
| 7,469 | 1,947 | 13.95 | [M-H]- | 375.2887 | Lithocholic acid |
| 7,469 | 1,947 | 13.95 | [M-H]- | 375.2887 | Lithocholic acid |
| 5,681 | 1,678 | 13.95 | [M-H]- | 375.2887 | Lithocholic acid |
| 23,300 | 2,041 | 12.97 | [M-H]- | 482.2933 | LITHOCHOLYLTAURINE |
| 45,040 | 13,190 | 1.31 | [M-H]- | 130.0873 | L-Leucine |
| 109,800 | 38,210 | 1.22 | [M-H]- | 133.0144 | L-Malic acid |
| 45,040 | 13,190 | 1.31 | [M-H]- | 130.0873 | L-Norleucine |
| 45,020 | 13,440 | 0.96 | [M-H]- | 131.0823 | L-ORNITHINE |
| 1,392,000 | 245,100 | 4.82 | [M-H]- | 164.0716 | L-Phenylalanine |
| 74,970 | 20,640 | 0.91 | [M-H]- | 179.056 | L-Sorbose |
| 3,607,000 | 704,100 | 5.57 | [M-H]- | 203.0828 | L-Tryptophan |
| 586,900 | 84,560 | 2.67 | [M-H]- | 180.0668 | L-Tyrosine |
| 16,920 | 2,803 | 0.77 | [M-H]- | 145.0983 | LYSINE |
| 110,000 | 37,690 | 1.22 | [M-H]- | 133.0144 | L-苹果L-Malic acid |
| 109,800 | 38,210 | 1.22 | [M-H]- | 133.0144 | MALATE |
| 109,800 | 38,210 | 1.22 | [M-H]- | 133.0144 | Malic acid |
| 7,181 | 1,910 | 6.25 | [M-H]- | 151.0401 | Mandelic Acid |
| 1,180 | 319 | 6.26 | [M-H]- | 107.0502 | m-Cresol |
| 1,180 | 319 | 6.26 | [M-H]- | 107.0502 | m-Cresol |
| 4,880 | 616 | 2.36 | [M-H]- | 129.0194 | Mesaconic acid |
| 1,697 | 286 | 9.76 | [M-H]- | 165.0562 | m-EthoxybenzoIC ACID1 |
| 43,560 | 6,535 | 5.47 | [M-H]- | 145.0506 | METHYGLUTARATE |
| 4,729 | 356 | 5.98 | [M-H]- | 145.05 | METHYGLUTARATE |
| 4,729 | 356 | 5.98 | [M-H]- | 145.05 | Methylglutaric acid |
| 5,326 | 1,457 | 16.03 | [M-H]- | 293.2486 | Methyllinoleate |
| 59,110 | 8,143 | 1.5 | [M-H]- | 117.0193 | METHYLMALONATE |
| 43,210 | 4,559 | 2.37 | [M-H]- | 117.0193 | METHYLMALONATE |
| 59,110 | 8,143 | 1.5 | [M-H]- | 117.0193 | Methylmalonic acid |
| 43,210 | 4,559 | 2.37 | [M-H]- | 117.0193 | Methylmalonic acid |
| 13,330 | 3,784 | 17.98 | [M-H]- | 297.2796 | Methylstearate |
| 865,200 | 109,600 | 6.33 | [M-H]- | 129.0557 | MEVALOLACTONE |
| 857,900 | 108,500 | 6.33 | [M-H]- | 129.0557 | MEVALOLACTONE |
| 274,000 | 87,330 | 15.05 | [M-H]- | 227.2016 | Myristic acid |
| 11,200 | 1,230 | 1.63 | [M-H]- | 130.0509 | N-ACETYLALANINE |
| 25,950 | 6,907 | 1.24 | [M-H]- | 173.0567 | N-ACETYLASPARAGINE |
| 4,450 | 559 | 1.65 | [M-H]- | 174.0407 | N-ACETYLASPARTATE |
| 11,200 | 1,230 | 1.63 | [M-H]- | 130.0509 | N-Acetyl-L-alanine |
| 43,910 | 12,730 | 6.85 | [M-H]- | 172.0977 | N-ACETYLLEUCINE |
| 42,500 | 12,380 | 6.85 | [M-H]- | 172.0977 | N-Acetyl-L-leucine |
| 65,290 | 19,210 | 7.21 | [M-H]- | 206.0822 | N-Acetyl-L-phenylalanine |
| 64,210 | 19,740 | 7.42 | [M-H]- | 245.0931 | N-Acetyl-L-tryptophan |
| 65,290 | 19,210 | 7.21 | [M-H]- | 206.0822 | N-ACETYLPHENYLALANINE |
| 7,653 | 2,088 | 1.17 | [M-H]- | 102.0197 | N-FORMYLGLYCINE |
| 7,414 | 2,055 | 1.17 | [M-H]- | 102.0197 | N-FORMYLGLYCINE |
| 92,850 | 25,200 | 5.52 | [M-H]- | 176.0386 | N-Formyl-L-methionine_ |
| 90,980 | 24,830 | 5.52 | [M-H]- | 176.0386 | N-Formyl-L-methionine_ |
| 541 | 156 | 6 | [M-H]- | 144.1026 | N-Methyl-L-leucine hydrochloride |
| 14,650 | 4,061 | 17.98 | [M-H]- | 297.2796 | Nonadecanoic acid |
| 762 | 104 | 13.59 | [M-H]- | 297.2865 | Nonadecanoic acid |
| 45,040 | 13,190 | 1.31 | [M-H]- | 130.0873 | NORLEUCINE |
| 36,510 | 11,100 | 11.14 | [M-H]- | 389.2679 | Nutriacholic acid |
| 29,730 | 8,948 | 12.3 | [M-H]- | 389.2682 | Nutriacholic acid |
| 14,700 | 2,418 | 11.51 | [M-H]- | 389.2686 | Nutriacholic acid |
| 25,950 | 6,907 | 1.24 | [M-H]- | 173.0567 | Nα-Acetyl-L-asparagine |
| 38,250 | 8,978 | 8.01 | [M-H]- | 402.0772 | Ochratoxin A |
| 36,650 | 11,450 | 13.42 | [M-H]- | 313.2383 | Octadecanedioic acid |
| 3,291 | 789 | 14.03 | [M-H]- | 249.1487 | Octisalate |
| 2,481,000 | 648,300 | 16.3 | [M-H]- | 281.2485 | OLEATE |
| 3,112 | 301 | 17.48 | [M-H]- | 281.2477 | OLEATE |
| 2,481,000 | 648,300 | 16.3 | [M-H]- | 281.2485 | Oleic acid |
| 3,112 | 301 | 17.48 | [M-H]- | 281.2477 | Oleic acid |
| 45,020 | 13,440 | 0.96 | [M-H]- | 131.0823 | Ornithine |
| 586,900 | 84,560 | 2.67 | [M-H]- | 180.0668 | o-Tyrosine |
| 2,050 | 462 | 1.25 | [M-H]- | 88.9897 | OXALATE |
| 2,050 | 462 | 1.25 | [M-H]- | 88.9897 | OXALATE |
| 2,607 | 342 | 6.62 | [M-H]- | 130.9942 | Oxaloacetic acid |
| 60,400 | 18,550 | 1.25 | [M-H]- | 145.0142 | OXOGLUTARATE |
| 60,400 | 18,550 | 1.25 | [M-H]- | 145.0142 | Oxoglutaric acid |
| 2,208,000 | 673,100 | 16.15 | [M-H]- | 255.2332 | PALMITATE |
| 9,134 | 2,003 | 12.17 | [M-H]- | 255.2321 | PALMITATE |
| 2,936 | 770 | 14.04 | [M-H]- | 255.233 | PALMITATE |
| 1,037 | 191 | 14.96 | [M-H]- | 277.1295 | Pantetheine |
| 865,200 | 109,600 | 6.33 | [M-H]- | 129.0557 | PANTOLACTONE |
| 392,200 | 109,100 | 5.19 | [M-H]- | 218.1035 | PANTOTHENATE |
| 392,200 | 109,100 | 5.19 | [M-H]- | 218.1035 | Pantothenic acid |
| 2,614 | 234 | 4.75 | [M-H]- | 179.0561 | PARAXANTHINE |
| 57,260 | 19,300 | 15.62 | [M-H]- | 241.2172 | Pentadecanoic acid |
| 1,881 | 465 | 14.88 | [M-H]- | 241.219 | Pentadecanoic acid |
| 46,140 | 14,300 | 1.33 | [M-H]- | 112.9853 | PFEA (perfluoro-n-ethanoic acid) (neg) |
| 2,726 | 531 | 10.3 | [M-H]- | 112.9857 | PFEA (perfluoro-n-ethanoic acid) (neg) |
| 1,053 | 122 | 7.63 | [M-H]- | 182.95 | PFESi (perfluoroethane sulfinate) (neg) |
| 1,053 | 122 | 7.63 | [M-H]- | 182.95 | PFESi (perfluoroethane sulfinate) (neg) |
| 32,110 | 6,227 | 5.81 | [M-H]- | 93.0344 | PHENOL |
| 5,814 | 564 | 16.55 | [M-H]- | 93.0342 | PHENOL |
| 7,010 | 1,891 | 6.25 | [M-H]- | 151.0401 | Phenoxyacetic acid-1 |
| 425,500 | 124,700 | 6.71 | [M-H]- | 192.0665 | Phenylacetylglycine |
| 1,392,000 | 245,100 | 4.82 | [M-H]- | 164.0716 | PHENYLALANINE |
| 1,697 | 286 | 9.76 | [M-H]- | 165.0562 | Phenyllactate (Pla) |
| 65,790 | 7,334 | 6.17 | [M-H]- | 165.0192 | Phthalic acid |
| 63,680 | 7,104 | 6.17 | [M-H]- | 165.0192 | Phthalic acid |
| 54,500 | 11,180 | 6.26 | [M-H]- | 159.0662 | Pimelic acid |
| 1,697 | 286 | 9.76 | [M-H]- | 165.0562 | p-Methoxyphenylacetic acid |
| 17,850 | 4,595 | 9.68 | [M-H]- | 351.2166 | Prostaglandin D2 |
| 17,850 | 4,595 | 9.68 | [M-H]- | 351.2166 | Prostaglandin E2 |
| 4,519 | 715 | 10.19 | [M-H]- | 329.0031 | Pyrroloquinoline Quinone |
| 1,239 | 146 | 11.74 | [M-H]- | 329.0019 | Pyrroloquinoline Quinone |
| 2,314 | 259 | 9.66 | [M-H]- | 329.0053 | Pyrroloquinoline Quinone |
| 6,418 | 645 | 8.08 | [M-H]- | 166.0144 | QUINOLINATE |
| 895,600 | 65,720 | 2.52 | [M-H]- | 103.0401 | R-3-Hydroxybutyric acid |
| 4,608 | 316 | 6.27 | [M-H]- | 383.0959 | S-ADENOSYLHOMOCYSTEINE |
| 6,128 | 1,180 | 8.07 | [M-H]- | 137.0246 | Salicyclic Acid |
| 6,580 | 1,183 | 8.07 | [M-H]- | 137.0246 | SALICYLATE |
| 6,580 | 1,183 | 8.07 | [M-H]- | 137.0246 | Salicylic acid |
| 521,700 | 48,460 | 19.58 | [M-H]- | 265.1476 | Sambucinol |
| 64,330 | 15,460 | 15.55 | [M-H]- | 265.1465 | Sambucinol |
| 32,920 | 8,038 | 8.93 | [M-H]- | 265.1451 | Sambucinol |
| 34,900 | 8,423 | 16.3 | [M-H]- | 265.1478 | Sambucinol |
| 28,380 | 2,354 | 14.45 | [M-H]- | 265.1466 | Sambucinol |
| 59,170 | 19,230 | 1.04 | [M-H]- | 88.0405 | SARCOSINE |
| 182,000 | 45,350 | 8.47 | [M-H]- | 201.1133 | SEBACATE |
| 182,000 | 45,350 | 8.47 | [M-H]- | 201.1133 | Sebacic acid |
| 9,234 | 1,921 | 7.21 | [M-H]- | 253.0182 | Shikimate 3-phosphate |
| 840,700 | 198,300 | 17.3 | [M-H]- | 283.2643 | STEARATE |
| 840,700 | 198,300 | 17.3 | [M-H]- | 283.2643 | Stearic acid |
| 127,800 | 31,800 | 7.03 | [M-H]- | 173.0819 | SUBERATE |
| 30,460 | 7,312 | 7.53 | [M-H]- | 173.0816 | SUBERATE |
| 127,800 | 31,800 | 7.03 | [M-H]- | 173.0819 | Suberic acid |
| 59,110 | 8,143 | 1.5 | [M-H]- | 117.0193 | SUCCINATE |
| 43,210 | 4,559 | 2.37 | [M-H]- | 117.0193 | SUCCINATE |
| 59,110 | 8,143 | 1.5 | [M-H]- | 117.0193 | Succinic acid |
| 43,210 | 4,559 | 2.37 | [M-H]- | 117.0193 | Succinic acid |
| 47,950 | 11,930 | 0.93 | [M-H]- | 124.0072 | TAURINE |
| 819,700 | 178,200 | 9.44 | [M-H]- | 514.2829 | Taurocholic acid |
| 319,900 | 71,710 | 8.53 | [M-H]- | 514.283 | Taurocholic acid |
| 9,780 | 2,070 | 10.04 | [M-H]- | 514.2659 | Taurocholic acid |
| 144,600 | 24,450 | 8.98 | [M-H]- | 498.2878 | Taurodeoxycholic acid |
| 65,790 | 7,334 | 6.17 | [M-H]- | 165.0192 | Terephthalic Acid |
| 58,280 | 16,490 | 11.03 | [M-H]- | 257.1756 | Tetradecanedioic acid |
| 2,614 | 234 | 4.75 | [M-H]- | 179.0561 | THEOPHYLLINE |
| 248,200 | 42,130 | 9.18 | [M-H]- | 369.2273 | Thromboxane B2 |
| 78,520 | 16,030 | 4.85 | [M-H]- | 241.0829 | THYMIDINE |
| 7,022 | 1,335 | 4.85 | [M-H]- | 125.0357 | THYMINE |
| 6,807 | 1,334 | 4.85 | [M-H]- | 125.0357 | Thymine-1 |
| 15,220 | 4,551 | 9.35 | [M-H]- | 775.6771 | THYROXINE |
| 97,090 | 15,290 | 1.33 | [M-H]- | 173.0092 | trans-Aconitic acid |
| 16,630 | 2,050 | 2.37 | [M-H]- | 173.0092 | trans-Aconitic acid |
| 16,630 | 2,050 | 2.37 | [M-H]- | 173.0092 | trans-Aconitic acid |
| 66,670 | 12,170 | 4.82 | [M-H]- | 147.045 | trans-Cinnamic acid |
| 21,070 | 4,663 | 9.36 | [M-H]- | 227.1287 | Traumatic acid |
| 6,659 | 1,625 | 10.06 | [M-H]- | 227.1285 | Traumatic acid |
| 1,697 | 286 | 9.76 | [M-H]- | 165.0562 | Tropic acid |
| 586,900 | 84,560 | 2.67 | [M-H]- | 180.0668 | TYROSINE |
| 62,770 | 20,680 | 1.28 | [M-H]- | 180.0666 | TYROSINE |
| 158,100 | 47,630 | 9.15 | [M-H]- | 215.129 | Undecanedioic acid |
| 941,100 | 298,600 | 1.25 | [M-H]- | 167.0213 | URATE |
| 629,600 | 80,600 | 2.06 | [M-H]- | 167.0212 | URATE |
| 74,110 | 9,819 | 2.73 | [M-H]- | 243.0623 | URIDINE |
| 416,400 | 106,900 | 10.88 | [M-H]- | 391.284 | URSODEOXYCHOLATE |
| 357,400 | 103,000 | 12.25 | [M-H]- | 391.284 | URSODEOXYCHOLATE |
| 48,420 | 10,750 | 11.32 | [M-H]- | 391.2842 | URSODEOXYCHOLATE |
| 901 | 248 | 9.64 | [M-H]- | 391.2754 | URSODEOXYCHOLATE |
| 416,400 | 106,900 | 10.88 | [M-H]- | 391.284 | Ursodeoxycholic acid |
| 357,400 | 103,000 | 12.25 | [M-H]- | 391.284 | Ursodeoxycholic acid |
| 48,420 | 10,750 | 11.32 | [M-H]- | 391.2842 | Ursodeoxycholic acid |
| 901 | 248 | 9.64 | [M-H]- | 391.2754 | Ursodeoxycholic acid |
| 416,400 | 106,900 | 10.88 | [M-H]- | 391.284 | Ursodeoxycholic acid |
| 357,400 | 103,000 | 12.25 | [M-H]- | 391.284 | Ursodeoxycholic acid |
| 48,420 | 10,750 | 11.32 | [M-H]- | 391.2842 | Ursodeoxycholic acid |
| 901 | 248 | 9.64 | [M-H]- | 391.2754 | Ursodeoxycholic acid |
| 369,900 | 95,220 | 10.88 | [M-H]- | 391.284 | Ursodeoxycholic acid |
| 324,100 | 93,590 | 12.25 | [M-H]- | 391.284 | Ursodeoxycholic acid |
| 43,040 | 9,586 | 11.32 | [M-H]- | 391.2842 | Ursodeoxycholic acid |
| 2,481,000 | 648,300 | 16.3 | [M-H]- | 281.2485 | Vaccenic acid |
| 3,112 | 301 | 17.48 | [M-H]- | 281.2477 | Vaccenic acid |
| 62,480 | 17,860 | 4.85 | [M-H]- | 283.0686 | Xanthosine |
| 3,519 | 430 | 9.51 | [M-H]- | 363.05 | XANTHOSINE-MONOPHOSPHATE |
| 58,590 | 8,019 | 1.5 | [M-H]- | 117.0193 | Amber Acid |
| 41,940 | 4,514 | 2.37 | [M-H]- | 117.0193 | Amber Acid |
| 4,124,000 | 1,238,000 | 15.55 | [M-H]- | 279.2328 | Linoleic acid |
| 324,100 | 93,590 | 12.25 | [M-H]- | 391.284 | Deoxycholic acid |
| 43,040 | 9,586 | 11.32 | [M-H]- | 391.2842 | Deoxycholic acid |
| 1,166 | 398 | 7.13 | [M-H]- | 603.3853 | Ziyuglycoside II |
| 652 | 189 | 6.38 | [M-H]- | 383.2394 | Kirenol +HCOOH |
| 6,128 | 1,180 | 8.07 | [M-H]- | 137.0246 | 4-Hydroxybenzoic acid |
| 71,030 | 9,448 | 2.73 | [M-H]- | 243.0623 | Uridine |
| 48,150 | 4,641 | 1.54 | [M-H]- | 130.0872 | Isoleucine |
| 103,200 | 29,370 | 6.37 | [M-H]- | 383.1917 | Isorhynchophylline |
| 1,989 | 594 | 13.37 | [M-H]- | 741.3868 | Periplocoside +HCOOH |
| 744,300 | 239,300 | 1.25 | [M-H]- | 191.0199 | Citric acid |
| 132,400 | 14,940 | 1.9 | [M-H]- | 191.0198 | Citric acid |
| 6,128 | 1,180 | 8.07 | [M-H]- | 137.0246 | salicylic acid |
| 369,900 | 95,220 | 10.88 | [M-H]- | 391.284 | Ursodeoxycholic Acid |
| 324,100 | 93,590 | 12.25 | [M-H]- | 391.284 | Ursodeoxycholic Acid |
| 43,040 | 9,586 | 11.32 | [M-H]- | 391.2842 | Ursodeoxycholic Acid |
| 737 | 220 | 12.6 | [M-H]- | 455.3605 | Ursolic Acid |
| 1,167,000 | 254,300 | 9.38 | [M-H]- | 498.2877 | Tauroursodeoxycholic acid |
| 488,400 | 68,950 | 10.92 | [M-H]- | 498.2881 | Tauroursodeoxycholic acid |
| 47,270 | 11,740 | 0.93 | [M-H]- | 124.0072 | Taurine |
| 369,900 | 95,220 | 10.88 | [M-H]- | 391.284 | Hyodeoxycholic acid |
| 324,100 | 93,590 | 12.25 | [M-H]- | 391.284 | Hyodeoxycholic acid |
| 43,040 | 9,586 | 11.32 | [M-H]- | 391.2842 | Hyodeoxycholic acid |
| 58,590 | 8,019 | 1.5 | [M-H]- | 117.0193 | Succinic acid |
| 41,940 | 4,514 | 2.37 | [M-H]- | 117.0193 | Succinic acid |
| 172,100 | 51,950 | 0.96 | [M-H]- | 174.0885 | Citrulline |
| 81,240 | 26,070 | 9.75 | [M-H]- | 448.3057 | Glycoursodeoxycholic acid |
| 34,940 | 10,320 | 10.98 | [M-H]- | 448.3053 | Glycoursodeoxycholic acid |
| 158,100 | 47,140 | 9.67 | [M-H]- | 464.3008 | Glycocholic acid |
| 24,760 | 4,921 | 9.04 | [M-H]- | 464.301 | Glycocholic acid |
| 21,040 | 6,171 | 8.9 | [M-H]- | 464.3 | Glycocholic acid |
| 737 | 220 | 12.6 | [M-H]- | 455.3605 | Betulinic acid |
| 377 | 107 | 9.68 | [M-H]- | 453.1417 | Nodakenin +HCOOH |
| 22,760 | 6,995 | 0.9 | [M-H]- | 154.0619 | Histidine |
| 73,700 | 21,630 | 1.25 | [M-H]- | 175.0248 | Vitamin C |
| 19,430 | 6,161 | 9.03 | [M-H]- | 165.0557 | Ethyl 4-hydroxybenzoate |
| 65,930 | 12,050 | 4.82 | [M-H]- | 147.045 | cinnamic acid |
| 5,029 | 1,395 | 0.95 | [M-H]- | 147.0491 | cinnamic acid |
| 52,780 | 9,086 | 17.7 | [M-H]- | 583.2549 | Bilirubin |
| 761,800 | 192,200 | 10.63 | [M-H]- | 407.2793 | Cholan-24-oic acid |
| 110,200 | 30,850 | 9.72 | [M-H]- | 407.2792 | Cholan-24-oic acid |
| 3,511,000 | 684,100 | 5.57 | [M-H]- | 203.0828 | L-Tryptophan |
| 1,372,000 | 241,600 | 4.82 | [M-H]- | 164.0716 | Phenprobamate |
| 1,372,000 | 241,600 | 4.82 | [M-H]- | 164.0716 | Phenylalanine |
| 1,453 | 373 | 5.8 | [M-H]- | 173.0457 | Shikimic acid |
| 564 | 284 | 15.19 | [M-H]- | 327.1672 | crocetin |
| 369,900 | 95,220 | 10.88 | [M-H]- | 391.284 | Chenodeoxycholic acid |
| 324,100 | 93,590 | 12.25 | [M-H]- | 391.284 | Chenodeoxycholic acid |
| 43,040 | 9,586 | 11.32 | [M-H]- | 391.2842 | Chenodeoxycholic acid |
| 737 | 220 | 12.6 | [M-H]- | 455.3605 | Oleanolic acid |

Note: NEG, negative.

**Table S2. Quantification of drug-containing serum in the Control group (POS mode)**

| **Area** | **Height** | **Retention Time** | **Adduct / Charge** | **Found At Mass** | **Library Hit** |
| --- | --- | --- | --- | --- | --- |
| 42890 | 9483 | 1.01 | [M+H]+ | 104.0703 | (S)-β-Aminoisobutyric Acid |
| 22240 | 6178 | 10.42 | [M+H]+ | 245.1748 | 1,11-Undecanedicarboxylic acid |
| 33870 | 6077 | 7.76 | [M+H]+ | 107.0697 | 1,2,4-Butanetriol |
| 43740 | 2375 | 19.91 | [M+H]+ | 109.0759 | 1,3-Phenylenediamine |
| 2373 | 290 | 1.55 | [M+H]+ | 107.0847 | 1,4-Xylene |
| 2397 | 569 | 0.1 | [M+H]+ | 107.0846 | 1,4-Xylene |
| 6349 | 969 | 6.8 | [M+H]+ | 107.0851 | 1,4-Xylene |
| 138600 | 41880 | 12.74 | [M+H]+ | 301.2168 | 13-cis-Retinoic acid |
| 40550 | 11220 | 10.73 | [M+H]+ | 253.1796 | 15-Hydroxyculmorone |
| 54620 | 15140 | 14.78 | [M+H]+ | 303.2321 | 17a-Methyltestosterone |
| 54620 | 15140 | 14.78 | [M+H]+ | 303.2321 | 17a-Methyltestosterone |
| 335200 | 114000 | 1.27 | [M+H]+ | 130.0858 | 1-Aminocyclopentanecarboxylic acid |
| 39400 | 3626 | 1.55 | [M+H]+ | 130.0861 | 1-Aminocyclopentanecarboxylic acid |
| 51190 | 5550 | 5.78 | [M+H]+ | 100.0753 | 1-Methyl-2-pyrrolidone |
| 78960 | 5927 | 14.44 | [M+H]+ | 100.0757 | 1-Methyl-2-pyrrolidone |
| 51190 | 5550 | 5.78 | [M+H]+ | 100.0753 | 1-Methyl-2-pyrrolidone |
| 36900 | 3420 | 2.45 | [M+H]+ | 100.0756 | 1-Methyl-2-pyrrolidone |
| 38460 | 4221 | 13.88 | [M+H]+ | 100.0755 | 1-Methyl-2-pyrrolidone |
| 9035 | 965 | 2.91 | [M+H]+ | 282.1184 | 1-Methyladenosine |
| 168700 | 39260 | 5.66 | [M+H]+ | 144.0806 | 1-NAPHTHYLAMINE |
| 4272 | 700 | 4.83 | [M+H]+ | 147.0655 | 2,2-Dimethylsuccinic acid |
| 1728 | 207 | 17.86 | [M+H]+ | 105.0691 | 2,3-DIAMINOPROPIONATE |
| 2601 | 277 | 1.53 | [M+H]+ | 101.0589 | 2,3-Pentanedione |
| 35920 | 13120 | 19.48 | [M+H]+ | 122.0964 | 2,4-Dimethylaniline |
| 2750 | 606 | 9.33 | [M+H]+ | 97.0642 | 2,5-Dimethylfuran |
| 35920 | 13120 | 19.48 | [M+H]+ | 122.0964 | 2,6-Dimethylaniline |
| 42890 | 9483 | 1.01 | [M+H]+ | 104.0703 | 2-Aminoisobutyric Acid |
| 35250 | 8881 | 5.69 | [M+H]+ | 200.0474 | 2-Chloro-L-phenylalanine |
| 28030 | 5896 | 5.6 | [M+H]+ | 133.0857 | 2-Hydroxyisocaproic Acid |
| 207500 | 63280 | 6.77 | [M+H]+ | 194.0812 | 2-Methylhippuric acid |
| 8350 | 2253 | 6.11 | [M+H]+ | 174.055 | 2-QUINOLINECARBOXYLATE |
| 69170 | 5114 | 3.29 | [M+H]+ | 154.0498 | 3-AMINO-4-HYDROXYBENZOATE |
| 50790 | 9962 | 4.9 | [M+H]+ | 154.0498 | 3-AMINO-4-HYDROXYBENZOATE |
| 69170 | 5114 | 3.29 | [M+H]+ | 154.0498 | 3-AMINO-5-HYDROXYBENZOATE |
| 50790 | 9962 | 4.9 | [M+H]+ | 154.0498 | 3-AMINO-5-HYDROXYBENZOATE |
| 42890 | 9483 | 1.01 | [M+H]+ | 104.0703 | 3-Aminobutyric acid |
| 426800 | 133400 | 8.73 | [M+H]+ | 190.0856 | 3-Indolepropionic acid |
| 34760 | 10030 | 7.06 | [M+H]+ | 148.0753 | 3-METHYL-2-OXINDOLE |
| 6319 | 967 | 5.83 | [M+H]+ | 158.0806 | 3-Methylcrotonyl Glycine |
| 81140 | 16130 | 5.66 | [M+H]+ | 132.0805 | 3-Methylindole |
| 220200 | 69910 | 1.12 | [M+H]+ | 138.0547 | 3-Pyridylacetic acid |
| 220200 | 69910 | 1.12 | [M+H]+ | 138.0547 | 4-AMINOBENZOATE |
| 217000 | 68680 | 1.12 | [M+H]+ | 138.0547 | 4-Aminobenzoic acid |
| 32050 | 9099 | 9.11 | [M+H]+ | 345.243 | 4-Beta-hydroxystanozolol |
| 1134000 | 307000 | 1.32 | [M+H]+ | 165.0545 | 4-COUMARATE |
| 557300 | 59160 | 3.04 | [M+H]+ | 165.0545 | 4-COUMARATE |
| 10240 | 1895 | 9.49 | [M+H]+ | 151.0754 | 4-Ethylbenzoic Acid |
| 211200 | 36250 | 1.3 | [M+H]+ | 123.0441 | 4-HYDROXYBENZALDEHYDE |
| 60370 | 6701 | 3.05 | [M+H]+ | 123.0439 | 4-HYDROXYBENZALDEHYDE |
| 263600 | 56880 | 5.66 | [M+H]+ | 146.0599 | 4-Hydroxyquinoline |
| 3934 | 1058 | 6.22 | [M+H]+ | 146.0599 | 4-Hydroxyquinoline |
| 6571 | 1231 | 7.86 | [M+H]+ | 177.0545 | 4-甲基伞形酮 Hymecromone |
| 108200 | 27490 | 5.07 | [M+H]+ | 192.0657 | 5-HYDROXYINDOLEACETATE |
| 10280 | 2971 | 6.22 | [M+H]+ | 192.0649 | 5-HYDROXYINDOLEACETATE |
| 108200 | 27490 | 5.07 | [M+H]+ | 192.0657 | 5-Hydroxyindoleacetic acid |
| 10280 | 2971 | 6.22 | [M+H]+ | 192.0649 | 5-Hydroxyindoleacetic acid |
| 25240 | 3052 | 5.28 | [M+H]+ | 221.092 | 5-HYDROXYTRYPTOPHAN |
| 8680 | 725 | 2.64 | [M+H]+ | 221.0915 | 5-HYDROXYTRYPTOPHAN |
| 42710 | 4566 | 2.87 | [M+H]+ | 126.0659 | 5-METHYLCYTOSINE |
| 29520 | 8667 | 5.61 | [M+H]+ | 298.0967 | 5-Methylthioadenosine |
| 11610 | 1288 | 4.97 | [M+H]+ | 132.1025 | 6-Aminocaproic Acid |
| 3934 | 1058 | 6.22 | [M+H]+ | 146.0599 | 8-Hydroxyquinoline |
| 671 | 113 | 19.57 | [M+H]+ | 279.0309 | Acecarbromal |
| 32960 | 2742 | 5.29 | [M+H]+ | 89.0596 | ACETOIN |
| 17190 | 2561 | 6.03 | [M+H]+ | 89.0596 | ACETOIN |
| 17650 | 1616 | 9.01 | [M+H]+ | 89.0594 | ACETOIN |
| 3811000 | 323700 | 1.72 | [M+H]+ | 132.1018 | Alloisoleucine |
| 11610 | 1288 | 4.97 | [M+H]+ | 132.1025 | Alloisoleucine |
| 164500 | 47100 | 0.94 | [M+H]+ | 120.065 | ALLOTHREONINE |
| 397000 | 128300 | 14.89 | [M+H]+ | 279.2317 | Alpha-Linolenic acid |
| 37120 | 8994 | 1.08 | [M+H]+ | 162.0762 | AMINOADIPATE |
| 37120 | 8994 | 1.08 | [M+H]+ | 162.0762 | Aminoadipic acid |
| 1597 | 276 | 5.01 | [M+H]+ | 256.0909 | Aminoflubendazol |
| 3258 | 363 | 7.4 | [M+H]+ | 233.1334 | Aminoglutethimide |
| 577 | 136 | 2.31 | [M+H]+ | 85.047 | Amitrole |
| 7057 | 1540 | 5.08 | [M+H]+ | 94.065 | ANILINE |
| 220200 | 69910 | 1.12 | [M+H]+ | 138.0547 | ANTHRANILATE |
| 146800 | 30460 | 11.81 | [M+H]+ | 448.3423 | Arachidorylcarnitine(C20:4) |
| 25000 | 6666 | 4.91 | [M+H]+ | 175.1188 | ARGININE |
| 25770 | 6279 | 8.93 | [M+H]+ | 267.1593 | Aspterric acid |
| 142600 | 19250 | 4.98 | [M+H]+ | 194.1179 | BDB |
| 43330 | 3295 | 1.93 | [M+H]+ | 194.1174 | BDB |
| 36420 | 16330 | 19.47 | [M+H]+ | 194.1179 | BDB |
| 2024000 | 606300 | 1.01 | [M+H]+ | 118.0859 | BETAINE |
| 6888 | 3731 | 19.47 | [M+H]+ | 118.0861 | BETAINE |
| 167900 | 27480 | 17.71 | [M+H]+ | 585.2696 | BILIRUBIN |
| 76180 | 17540 | 9.25 | [M+H]+ | 585.2703 | BILIRUBIN |
| 19130 | 5288 | 11.81 | [M+H]+ | 585.2703 | BILIRUBIN |
| 13630 | 2130 | 17.71 | [M+H]+ | 583.2531 | BILIVERDIN |
| 57980 | 11440 | 17.48 | [M+H]+ | 391.2842 | BIS(2-ETHYLHEXYL)PHTHALATE |
| 32960 | 2742 | 5.29 | [M+H]+ | 89.0596 | BUTANOATE |
| 17190 | 2561 | 6.03 | [M+H]+ | 89.0596 | BUTANOATE |
| 17650 | 1616 | 9.01 | [M+H]+ | 89.0594 | BUTANOATE |
| 52500 | 13010 | 11.91 | [M+H]+ | 195.1015 | Butylparaben |
| 256100 | 40190 | 5.41 | [M+H]+ | 232.1547 | Butyrylcarnitine(C4) |
| 93710 | 22240 | 5.11 | [M+H]+ | 149.0598 | Cinnamic acid |
| 7717 | 1864 | 8.41 | [M+H]+ | 143.1056 | Cis-3-Hexenylacetate |
| 1004000 | 219800 | 11.82 | [M+H]+ | 424.3422 | Ciscis9,12-Octadecadienoylcarnitine(C18:2) |
| 8498 | 2385 | 10.16 | [M+H]+ | 153.1273 | cis-Citral |
| 4191 | 1104 | 9.16 | [M+H]+ | 153.1273 | cis-Citral |
| 456200 | 123500 | 0.98 | [M+H]+ | 176.1029 | Citrulline |
| 7043000 | 2192000 | 1.06 | [M+H]+ | 132.0764 | CREATINE |
| 370500 | 117300 | 1.27 | [M+H]+ | 244.0929 | CYTIDINE |
| 593600 | 183600 | 1.27 | [M+H]+ | 112.0502 | CYTOSINE |
| 229100 | 26810 | 2.14 | [M+H]+ | 112.0505 | CYTOSINE |
| 40170 | 11010 | 9.41 | [M+H]+ | 316.2485 | Decanoylcarnitine (C10) |
| 296100 | 85870 | 1.14 | [M+H]+ | 146.1174 | DEOXYCARNITINE |
| 779800 | 210500 | 11.37 | [M+H]+ | 393.2864 | DEOXYCHOLATE |
| 122600 | 14730 | 2.14 | [M+H]+ | 228.098 | Deoxycytidine |
| 347700 | 110700 | 1.27 | [M+H]+ | 228.0979 | DEOXYCYTIDINE |
| 489300 | 169400 | 0.93 | [M+H]+ | 147.0759 | D-Glutamine |
| 111100 | 10520 | 1.87 | [M+H]+ | 87.0439 | DIACETYL |
| 861400 | 143900 | 13.79 | [M+H]+ | 279.1593 | Dibutyl phthalate |
| 155900 | 11190 | 14.31 | [M+H]+ | 279.1593 | Dibutyl phthalate |
| 74580 | 4551 | 5.47 | [M+H]+ | 279.1593 | Dibutyl phthalate |
| 44090 | 5852 | 5.79 | [M+H]+ | 279.1591 | Dibutyl phthalate |
| 951700 | 154400 | 13.79 | [M+H]+ | 279.1593 | DIISOBUTYL PHTHALATE |
| 153900 | 10810 | 5.37 | [M+H]+ | 279.1593 | DIISOBUTYL PHTHALATE |
| 159700 | 11830 | 14.32 | [M+H]+ | 279.1593 | DIISOBUTYL PHTHALATE |
| 57040 | 7264 | 5.79 | [M+H]+ | 279.1591 | DIISOBUTYL PHTHALATE |
| 4563 | 1161 | 9.15 | [M+H]+ | 217.1435 | Dimethyl azelate |
| 6072 | 1583 | 1.27 | [M+H]+ | 91.039 | DL-Glyceraldehyde |
| 114000 | 22300 | 0.79 | [M+H]+ | 147.1126 | D-Lysine |
| 6267 | 1065 | 5.2 | [M+H]+ | 180.0888 | D-MANNOSAMINE |
| 8814 | 2399 | 9.8 | [M+H]+ | 231.1593 | Dodecanedioic acid |
| 8814 | 2399 | 9.8 | [M+H]+ | 231.1593 | Dodecanedioic acid |
| 8814 | 2399 | 9.8 | [M+H]+ | 231.1593 | Dodecanedioic acid |
| 6985 | 2283 | 9.8 | [M+H]+ | 231.1593 | Dodecanedioic acid |
| 1710000 | 526900 | 13.35 | [M+H]+ | 303.2321 | EICOSAPENTAENOATE |
| 22810 | 4730 | 13.96 | [M+H]+ | 283.2629 | ELAIDATE |
| 12490 | 2296 | 7.69 | [M+H]+ | 886.529 | Emamectin B1a |
| 51140 | 14030 | 7.64 | [M+H]+ | 872.5406 | Emamectin B1b |
| 12490 | 2296 | 7.69 | [M+H]+ | 886.529 | Emamectin benzoate B1a |
| 42610 | 11820 | 15.53 | [M+H]+ | 269.2265 | Epimetendiol |
| 14380 | 3282 | 9.54 | [M+H]+ | 249.149 | Eremofortin B |
| 18600 | 4685 | 11.91 | [M+H]+ | 167.0702 | Ethylsalicylate |
| 614 | 135 | 16.3 | [M+H]+ | 223.2063 | Farnesol |
| 16870 | 5021 | 11.08 | [M+H]+ | 328.0791 | Fluazifop free acid |
| 30720 | 8678 | 0.93 | [M+H]+ | 261.0374 | FRUCTOSE 6-PHOSPHATE |
| 6267 | 1065 | 5.2 | [M+H]+ | 180.0888 | GALACTOSAMINE |
| 137000 | 34440 | 12.96 | [M+H]+ | 279.2317 | GAMMA-LINOLENATE |
| 27770 | 8400 | 11.63 | [M+H]+ | 279.2321 | GAMMA-LINOLENATE |
| 30260 | 7836 | 13.68 | [M+H]+ | 279.2321 | GAMMA-LINOLENATE |
| 6267 | 1065 | 5.2 | [M+H]+ | 180.0888 | GLUCOSAMINE |
| 30720 | 8678 | 0.93 | [M+H]+ | 261.0374 | Glucose 1-phosphate |
| 30720 | 8678 | 0.93 | [M+H]+ | 261.0374 | GLUCOSE 6-PHOSPHATE |
| 233500 | 63450 | 0.95 | [M+H]+ | 148.0601 | GLUTAMATE |
| 489300 | 169400 | 0.93 | [M+H]+ | 147.0759 | GLUTAMINE |
| 78620 | 15260 | 1.2 | [M+H]+ | 308.0907 | Glutathione |
| 78620 | 15260 | 1.2 | [M+H]+ | 308.0907 | Glutathione |
| 293700 | 81070 | 0.99 | [M+H]+ | 258.11 | Glycerophosphocholine |
| 78830 | 21680 | 9.75 | [M+H]+ | 450.3215 | GLYCOCHENODEOXYCHOLATE |
| 66120 | 19320 | 10.98 | [M+H]+ | 450.3212 | GLYCOCHENODEOXYCHOLATE |
| 143000 | 41910 | 9.67 | [M+H]+ | 466.3167 | GLYCOCHOLATE |
| 143000 | 41910 | 9.67 | [M+H]+ | 466.3167 | Glycocholic acid |
| 24040 | 4301 | 1.25 | [M+H]+ | 173.0915 | Glycyl-L-Proline |
| 51130 | 16340 | 11.95 | [M+H]+ | 362.0404 | Haloxyfop-P |
| 135300 | 38740 | 6.95 | [M+H]+ | 569.3134 | Helvolic acid |
| 9120 | 922 | 5.85 | [M+H]+ | 271.2627 | HEPTADECANOATE |
| 9120 | 922 | 5.85 | [M+H]+ | 271.2627 | HEPTADECANOATE |
| 52260 | 16550 | 7 | [M+H]+ | 260.1856 | Hexanoylcarnitine (C6) |
| 36510 | 8440 | 6.41 | [M+H]+ | 180.0657 | HIPPURATE |
| 36510 | 8440 | 6.41 | [M+H]+ | 180.0657 | Hippuric acid |
| 63610 | 20500 | 1.14 | [M+H]+ | 190.1183 | Homocitrulline |
| 164500 | 47100 | 0.94 | [M+H]+ | 120.065 | HOMOSERINE |
| 10240 | 1895 | 9.49 | [M+H]+ | 151.0754 | Hydrocinnamic acid |
| 10240 | 1895 | 9.49 | [M+H]+ | 151.0754 | Hydrocinnamic acid |
| 8399 | 1756 | 9.49 | [M+H]+ | 151.0754 | Hydrocinnamic acid |
| 779800 | 210500 | 11.37 | [M+H]+ | 393.2864 | Hyodeoxycholic Acid |
| 779800 | 210500 | 11.37 | [M+H]+ | 393.2864 | Hyodeoxycholic Acid |
| 292700 | 79280 | 11.37 | [M+H]+ | 393.2864 | Hyodeoxycholic Acid |
| 426800 | 133400 | 8.73 | [M+H]+ | 190.0856 | INDOLE-3-METHYL ACETATE |
| 551700 | 103500 | 4.97 | [M+H]+ | 160.0757 | INDOLEACETALDEHYDE |
| 34330 | 7655 | 5.66 | [M+H]+ | 160.0762 | INDOLEACETALDEHYDE |
| 57220 | 17130 | 8.02 | [M+H]+ | 176.0706 | Indoleacetic acid |
| 6840000 | 1525000 | 5.66 | [M+H]+ | 188.0708 | Indoleacrylic acid |
| 30850 | 7693 | 7.33 | [M+H]+ | 188.0708 | Indoleacrylic acid |
| 22970 | 6869 | 7.43 | [M+H]+ | 206.0814 | Indolelactic acid |
| 22970 | 6869 | 7.43 | [M+H]+ | 206.0814 | Indolelactic acid |
| 22820 | 6540 | 7.43 | [M+H]+ | 206.0814 | Indolelactic acid |
| 22150 | 5263 | 6.37 | [M+H]+ | 134.0603 | Indoxyl |
| 12130 | 2121 | 6.87 | [M+H]+ | 148.0391 | Isatin |
| 32960 | 2742 | 5.29 | [M+H]+ | 89.0596 | ISOBUTYRATE |
| 17190 | 2561 | 6.03 | [M+H]+ | 89.0596 | ISOBUTYRATE |
| 17650 | 1616 | 9.01 | [M+H]+ | 89.0594 | ISOBUTYRATE |
| 256100 | 40190 | 5.41 | [M+H]+ | 232.1547 | Isobutyrylcarnitine (isoC4) |
| 49690 | 14730 | 9.99 | [M+H]+ | 165.0914 | Isoeugenol |
| 3811000 | 323700 | 1.72 | [M+H]+ | 132.1018 | ISOLEUCINE |
| 11610 | 1288 | 4.97 | [M+H]+ | 132.1025 | ISOLEUCINE |
| 102600 | 10460 | 2.08 | [M+H]+ | 123.0553 | Isonicotinamide |
| 5717 | 492 | 5.41 | [M+H]+ | 271.2632 | Isopropyl myristate |
| 149800 | 45000 | 8.73 | [M+H]+ | 130.0646 | Isoquinoline |
| 29760 | 5952 | 5.66 | [M+H]+ | 130.0653 | Isoquinoline |
| 189600 | 44880 | 6.05 | [M+H]+ | 246.1701 | Isovalerylcarnitine |
| 2380 | 583 | 16.13 | [M+H]+ | 352.1962 | JWH-250 N-4 or 5-hydroxypentyl metabolite |
| 234100 | 54910 | 5.07 | [M+H]+ | 209.0924 | KYNURENINE |
| 2759000 | 878400 | 1.27 | [M+H]+ | 204.1232 | L-Acetylcarnitine |
| 1019000 | 125000 | 1.84 | [M+H]+ | 204.123 | L-Acetylcarnitine |
| 13840 | 1982 | 4.81 | [M+H]+ | 204.1232 | L-Acetylcarnitine |
| 156000 | 46940 | 0.94 | [M+H]+ | 120.065 | L-allo-Threonine |
| 25000 | 6666 | 4.91 | [M+H]+ | 175.1188 | L-Arginine |
| 158100 | 40840 | 10.38 | [M+H]+ | 344.2799 | LAUROYLCARNITINE |
| 716100 | 236600 | 1 | [M+H]+ | 162.1122 | L-CARNITINE |
| 716100 | 236600 | 1 | [M+H]+ | 162.1122 | L-CARNITINE |
| 3811000 | 323700 | 1.72 | [M+H]+ | 132.1018 | LEUCINE |
| 13400 | 3340 | 7.29 | [M+H]+ | 702.3994 | Leucomycin |
| 233500 | 63450 | 0.95 | [M+H]+ | 148.0601 | L-Glutamic acid |
| 489300 | 169400 | 0.93 | [M+H]+ | 147.0759 | L-Glutamine |
| 164500 | 47100 | 0.94 | [M+H]+ | 120.065 | L-Homoserine |
| 5249 | 1447 | 10.16 | [M+H]+ | 171.1382 | Linalool oxide |
| 393400 | 108600 | 15.55 | [M+H]+ | 281.2477 | LINOLEATE |
| 17640 | 5501 | 12.36 | [M+H]+ | 281.2474 | LINOLEATE |
| 3811000 | 323700 | 1.72 | [M+H]+ | 132.1018 | L-Leucine |
| 114000 | 22300 | 0.79 | [M+H]+ | 147.1126 | L-Lysine |
| 776700 | 250200 | 1.27 | [M+H]+ | 150.0581 | L-Methionine |
| 1045000 | 315900 | 1.08 | [M+H]+ | 116.0703 | L-Proline |
| 164500 | 47100 | 0.94 | [M+H]+ | 120.065 | L-Threonine |
| 6546000 | 1529000 | 5.66 | [M+H]+ | 205.0974 | L-Tryptophan |
| 3272000 | 893500 | 1.32 | [M+H]+ | 182.0811 | L-Tyrosine |
| 1640000 | 169500 | 3.04 | [M+H]+ | 182.0812 | L-Tyrosine |
| 763200 | 241800 | 1.26 | [M+H]+ | 118.086 | L-Valine |
| 114000 | 22300 | 0.79 | [M+H]+ | 147.1126 | LYSINE |
| 30720 | 8678 | 0.93 | [M+H]+ | 261.0374 | MANNOSE 6-PHOSPHATE |
| 30720 | 8678 | 0.93 | [M+H]+ | 261.0374 | MANNOSE 6-PHOSPHATE |
| 6586 | 1519 | 0.96 | [M+H]+ | 261.0368 | MANNOSE 6-PHOSPHATE |
| 110100 | 10500 | 1.87 | [M+H]+ | 87.0439 | Mesaconic acid_-CO2 |
| 1308 | 229 | 18.64 | [M+H]+ | 99.0797 | MESITYL OXIDE |
| 776700 | 250200 | 1.27 | [M+H]+ | 150.0581 | METHIONINE |
| 105500 | 27970 | 10.42 | [M+H]+ | 227.1644 | Methyl dihydrojasmonate |
| 2864 | 319 | 14.27 | [M+H]+ | 295.2643 | Methyllinoleate |
| 32830 | 9670 | 5.61 | [M+H]+ | 298.0967 | METHYLTHIOADENOSINE |
| 1727 | 345 | 5.73 | [M+H]+ | 280.1864 | Moxisylyte |
| 2373 | 290 | 1.55 | [M+H]+ | 107.0847 | m-Xylene |
| 2397 | 569 | 0.1 | [M+H]+ | 107.0846 | m-Xylene |
| 6349 | 969 | 6.8 | [M+H]+ | 107.0851 | m-Xylene |
| 570500 | 124300 | 11.29 | [M+H]+ | 372.311 | Myristoylcar-nitine (C14) |
| 207500 | 63280 | 6.77 | [M+H]+ | 194.0812 | N-(2-Methylbenzoyl)glycine |
| 35920 | 13120 | 19.48 | [M+H]+ | 122.0964 | N,N-Dimethylaniline |
| 230400 | 59010 | 1.14 | [M+H]+ | 203.1501 | N,N-DIMETHYLARGININE |
| 230400 | 59010 | 1.14 | [M+H]+ | 203.1501 | N,N-DIMETHYL-ARGININE |
| 1304 | 185 | 9.44 | [M+H]+ | 164.0391 | N-ACETYLCYSTEINE |
| 9591 | 2814 | 6.9 | [M+H]+ | 174.1122 | N-ACETYLLEUCINE |
| 9591 | 2814 | 6.9 | [M+H]+ | 174.1122 | N-ACETYLLEUCINE |
| 9588 | 2787 | 6.9 | [M+H]+ | 174.1122 | N-Acetyl-L-leucine |
| 9588 | 2787 | 6.9 | [M+H]+ | 174.1122 | N-Acetyl-L-leucine |
| 20050 | 6108 | 7.23 | [M+H]+ | 208.0969 | N-Acetyl-L-phenylalanine |
| 32060 | 10150 | 7.44 | [M+H]+ | 247.1075 | N-Acetyl-L-tryptophan |
| 20050 | 6108 | 7.23 | [M+H]+ | 208.0969 | N-ACETYLPHENYLALANINE |
| 35320 | 10700 | 1.27 | [M+H]+ | 189.1235 | N-ALPHA-ACETYLLYSINE |
| 13110 | 2927 | 10.89 | [M+H]+ | 275.2003 | Nandrolon |
| 13110 | 2927 | 10.89 | [M+H]+ | 275.2003 | Nandrolon |
| 1154 | 176 | 5.5 | [M+H]+ | 275.073 | Neburon |
| 1521 | 177 | 5.48 | [M+H]+ | 573.0982 | N-HOEAmP-FHxSE (N-hydroxyethyldimethylammoniopropyl perfluorohexanesulfonamidoethanol) (pos) |
| 102600 | 10460 | 2.08 | [M+H]+ | 123.0553 | Niacinamide |
| 102600 | 10460 | 2.08 | [M+H]+ | 123.0553 | NICOTINAMIDE |
| 104100 | 10430 | 2.08 | [M+H]+ | 123.0553 | NICOTINAMIDE |
| 2024000 | 606300 | 1.01 | [M+H]+ | 118.0859 | N-Methyl-a-aminoisobutyric acid |
| 6888 | 3731 | 19.47 | [M+H]+ | 118.0861 | N-Methyl-a-aminoisobutyric acid |
| 37120 | 8994 | 1.08 | [M+H]+ | 162.0762 | N-METHYLGLUTAMATE |
| 42890 | 9483 | 1.01 | [M+H]+ | 104.0703 | N-Methyl-L-alanine |
| 2858 | 794 | 9.45 | [M+H]+ | 414.269 | Norbuprenorphine |
| 3811000 | 323700 | 1.72 | [M+H]+ | 132.1018 | NORLEUCINE |
| 11610 | 1288 | 4.97 | [M+H]+ | 132.1025 | NORLEUCINE |
| 763200 | 241800 | 1.26 | [M+H]+ | 118.086 | NORVALINE |
| 337200 | 93690 | 10.06 | [M+H]+ | 391.2845 | Nutriacholic acid |
| 335700 | 101800 | 11.14 | [M+H]+ | 391.2845 | Nutriacholic acid |
| 215200 | 54080 | 10.63 | [M+H]+ | 391.2844 | Nutriacholic acid |
| 35320 | 10700 | 1.27 | [M+H]+ | 189.1235 | Nα-Acetyl-L-lysine |
| 99120 | 27180 | 16.85 | [M+H]+ | 284.2951 | Octadecanamide |
| 26990 | 7827 | 8.32 | [M+H]+ | 288.2167 | Octanoylcarnitine (C8) |
| 84400 | 17180 | 6.05 | [M+H]+ | 130.1588 | Octylamine |
| 22810 | 4730 | 13.96 | [M+H]+ | 283.2629 | OLEATE |
| 1681000 | 319300 | 12.37 | [M+H]+ | 426.3581 | OleylCarnitine(C18:1) |
| 3272000 | 893500 | 1.32 | [M+H]+ | 182.0811 | o-Tyrosine |
| 1640000 | 169500 | 3.04 | [M+H]+ | 182.0812 | o-Tyrosine |
| 67550 | 9521 | 5.36 | [M+H]+ | 279.1346 | Oxadixyl |
| 1647 | 207 | 11.62 | [M+H]+ | 279.126 | Oxadixyl |
| 6493 | 966 | 13.79 | [M+H]+ | 279.1264 | Oxadixyl |
| 482000 | 156700 | 0.93 | [M+H]+ | 130.0493 | OXOPROLINE |
| 81740 | 10950 | 2.3 | [M+H]+ | 130.0497 | OXOPROLINE |
| 2373 | 290 | 1.55 | [M+H]+ | 107.0847 | o-Xylene |
| 2397 | 569 | 0.1 | [M+H]+ | 107.0846 | o-Xylene |
| 6349 | 969 | 6.8 | [M+H]+ | 107.0851 | o-Xylene |
| 268900 | 76350 | 15.28 | [M+H]+ | 255.2321 | PALMITOLEATE |
| 1745000 | 335400 | 12.17 | [M+H]+ | 400.3421 | PALMITOYLCARNITINE |
| 220200 | 69910 | 1.12 | [M+H]+ | 138.0547 | p-Aminobenzoic acid |
| 69810 | 9767 | 5.36 | [M+H]+ | 279.1364 | Pantetheine |
| 677600 | 121800 | 5.31 | [M+H]+ | 220.1181 | PANTOTHENATE |
| 677600 | 121800 | 5.31 | [M+H]+ | 220.1181 | Pantothenic acid |
| 2225 | 512 | 11.66 | [M+H]+ | 135.1165 | p-Cymene |
| 9956000 | 2701000 | 6.67 | [M+H]+ | 476.3065 | PEG-10mer Ammonium adduct |
| 21130 | 3136 | 7.29 | [M+H]+ | 476.3069 | PEG-10mer Ammonium adduct |
| 10540000 | 2913000 | 6.82 | [M+H]+ | 520.3324 | PEG-11mer Ammonium adduct |
| 10180000 | 2840000 | 6.95 | [M+H]+ | 564.3584 | PEG-12mer Ammonium adduct |
| 3211000 | 886200 | 6.12 | [M+H]+ | 344.2282 | PEG-7mer Ammonium adduct |
| 6293000 | 1885000 | 6.32 | [M+H]+ | 388.2546 | PEG-8mer Ammonium adduct |
| 8879000 | 2637000 | 6.51 | [M+H]+ | 432.2807 | PEG-9mer Ammonium adduct |
| 8000 | 1251 | 10.25 | [M+H]+ | 167.1065 | Perillic acid |
| 36290 | 13190 | 19.48 | [M+H]+ | 122.0964 | PHENETHYLAMINE |
| 5729 | 594 | 3.04 | [M+H]+ | 95.0497 | PHENOL |
| 207500 | 63280 | 6.77 | [M+H]+ | 194.0812 | Phenylacetylglycine |
| 340100 | 116500 | 1.27 | [M+H]+ | 130.0858 | PIPECOLATE |
| 340100 | 116500 | 1.27 | [M+H]+ | 130.0858 | Pipecolic acid |
| 11430 | 1280 | 4.88 | [M+H]+ | 130.0856 | Pipecolic acid |
| 125200 | 7249 | 2.41 | [M+H]+ | 218.1389 | Propionylcarnitine (C3) |
| 60950 | 8101 | 4.49 | [M+H]+ | 218.1389 | Propionylcarnitine (C3) |
| 482000 | 156700 | 0.93 | [M+H]+ | 130.0493 | PYROGLUTAMATE |
| 81740 | 10950 | 2.3 | [M+H]+ | 130.0497 | PYROGLUTAMATE |
| 482000 | 156700 | 0.93 | [M+H]+ | 130.0493 | Pyroglutamic acid |
| 81740 | 10950 | 2.3 | [M+H]+ | 130.0497 | Pyroglutamic acid |
| 482000 | 156700 | 0.93 | [M+H]+ | 130.0493 | Pyrrolidonecarboxylic acid |
| 81740 | 10950 | 2.3 | [M+H]+ | 130.0497 | Pyrrolidonecarboxylic acid |
| 8350 | 2253 | 6.11 | [M+H]+ | 174.055 | Quinaldic acid |
| 33700 | 10190 | 6.4 | [M+H]+ | 377.1459 | Riboflavin |
| 25770 | 6279 | 8.93 | [M+H]+ | 267.1593 | Sambucinol |
| 27170 | 4462 | 5.3 | [M+H]+ | 101.0596 | Senecioic acid |
| 118400 | 23960 | 4.98 | [M+H]+ | 177.1023 | Serotonin |
| 4227 | 503 | 0.99 | [M+H]+ | 175.0566 | SHIKIMATE |
| 137600 | 19360 | 0.8 | [M+H]+ | 146.165 | SPERMIDINE |
| 32360 | 7326 | 11.32 | [M+H]+ | 302.3055 | SPHINGANINE |
| 534200 | 101100 | 13.04 | [M+H]+ | 428.3732 | Stearoylcarnitine (C18) |
| 18900 | 5537 | 0.94 | [M+H]+ | 126.0215 | TAURINE |
| 118800 | 23390 | 9.44 | [M+H]+ | 516.2987 | Taurocholic acid |
| 40520 | 7361 | 8.54 | [M+H]+ | 516.2992 | Taurocholic acid |
| 197900 | 41230 | 9.39 | [M+H]+ | 500.3037 | Taurodeoxycholic acid |
| 140800 | 20070 | 10.93 | [M+H]+ | 500.3039 | Taurodeoxycholic acid |
| 164500 | 47100 | 0.94 | [M+H]+ | 120.065 | THREONINE |
| 29160 | 8279 | 9.35 | [M+H]+ | 777.6937 | THYROXINE |
| 27170 | 4462 | 5.3 | [M+H]+ | 101.0596 | Tiglic acid |
| 504900 | 86940 | 13.79 | [M+H]+ | 279.16 | Tolycaine |
| 34800 | 5146 | 15.25 | [M+H]+ | 279.1604 | Tolycaine |
| 21350 | 2187 | 6 | [M+H]+ | 279.1593 | Tolycaine |
| 93710 | 22240 | 5.11 | [M+H]+ | 149.0598 | trans-Cinnamic acid |
| 220200 | 69910 | 1.12 | [M+H]+ | 138.0547 | TRIGONELLINE |
| 15330 | 1860 | 6.27 | [M+H]+ | 138.0912 | Tyramine |
| 3272000 | 893500 | 1.32 | [M+H]+ | 182.0811 | TYROSINE |
| 1640000 | 169500 | 3.04 | [M+H]+ | 182.0812 | TYROSINE |
| 21720 | 2264 | 3.27 | [M+H]+ | 113.0341 | URACIL |
| 542100 | 70710 | 2.3 | [M+H]+ | 169.0357 | URATE |
| 274200 | 86440 | 1.27 | [M+H]+ | 169.0355 | URATE |
| 79630 | 23790 | 1.27 | [M+H]+ | 245.0771 | URIDINE |
| 16460 | 1942 | 3.27 | [M+H]+ | 245.0769 | URIDINE |
| 779800 | 210500 | 11.37 | [M+H]+ | 393.2864 | URSODEOXYCHOLATE |
| 763200 | 241800 | 1.26 | [M+H]+ | 118.086 | VALINE |
| 127800 | 21340 | 4.9 | [M+H]+ | 153.041 | XANTHINE |
| 31160 | 9073 | 4.95 | [M+H]+ | 285.084 | XANTHOSINE |
| 164500 | 47100 | 0.94 | [M+H]+ | 120.065 | α-Methyl-DL-serine |
| 2864 | 319 | 14.27 | [M+H]+ | 295.2643 | Methyl linoleate |
| 3772000 | 320300 | 1.72 | [M+H]+ | 132.1018 | eucine |
| 2196000 | 400600 | 1.4 | [M+H]+ | 132.1017 | Leucine |
| 335200 | 114000 | 1.27 | [M+H]+ | 130.0858 | Pipecolinic acid |
| 39400 | 3626 | 1.55 | [M+H]+ | 130.0861 | Pipecolinic acid |
| 13100 | 3548 | 10.34 | [M+H]+ | 233.1536 | Alantolactone |
| 417200 | 117600 | 11.37 | [M+H]+ | 376.2594 | Tuberostemonine |
| 14380 | 3282 | 9.54 | [M+H]+ | 249.149 | Parthenolide |
| 703200 | 233300 | 1 | [M+H]+ | 162.1122 | L-Carnitine |
| 21240 | 5548 | 1.16 | [M+H]+ | 162.1123 | L-Carnitine |
| 2196000 | 400600 | 1.4 | [M+H]+ | 132.1017 | Isoleucine |
| 3772000 | 320300 | 1.72 | [M+H]+ | 132.1018 | Isoleucine |
| 2196000 | 400600 | 1.4 | [M+H]+ | 132.1017 | Isoleucine |
| 10050 | 918 | 4.97 | [M+H]+ | 132.1025 | Isoleucine |
| 13100 | 3548 | 10.34 | [M+H]+ | 233.1536 | Isoalantolactone |
| 1603000 | 496000 | 13.35 | [M+H]+ | 303.2321 | Kaurenoic acid |
| 54620 | 15140 | 14.78 | [M+H]+ | 303.2321 | Kaurenoic acid |
| 54620 | 15140 | 14.78 | [M+H]+ | 303.2321 | Kaurenoic acid |
| 13100 | 3548 | 10.34 | [M+H]+ | 233.1536 | Costunolide |
| 4191 | 1104 | 9.16 | [M+H]+ | 153.1273 | camphor; |
| 6137 | 1218 | 5.09 | [M+H]+ | 137.0456 | 6-Hydroxypurine |
| 104100 | 10430 | 2.08 | [M+H]+ | 123.0553 | Nicotinamide |
| 419700 | 121000 | 0.98 | [M+H]+ | 176.1029 | Citrulline |
| 58630 | 16670 | 9.86 | [M+H]+ | 251.1642 | Nardosinone |
| 121400 | 35660 | 9.67 | [M+H]+ | 466.3167 | Glycocholic acid |
| 2004000 | 603200 | 1.01 | [M+H]+ | 118.0859 | Betaine |
| 6700 | 3653 | 19.47 | [M+H]+ | 118.0861 | Betaine |
| 1229000 | 428500 | 1.23 | [M+H]+ | 144.1016 | Stachydrine hydrochloride |
| 5513 | 766 | 1.49 | [M+H]+ | 144.1016 | Stachydrine hydrochloride |
| 395100 | 156600 | 0.92 | [M+H]+ | 175.1189 | L(+)-Arginine |
| 23300 | 6443 | 4.91 | [M+H]+ | 175.1191 | L(+)-Arginine |
| 128100 | 38900 | 12.74 | [M+H]+ | 301.2168 | Vitamin A acid |
| 32460 | 9472 | 6.4 | [M+H]+ | 377.1459 | Vitamin B2 |
| 753600 | 240400 | 1.26 | [M+H]+ | 118.086 | L-Valine |
| 17170 | 4550 | 11.91 | [M+H]+ | 167.0702 | Ethyl 4-hydroxybenzoate |
| 92330 | 22000 | 5.11 | [M+H]+ | 149.0598 | cinnamic acid |
| 3519 | 964 | 8.9 | [M+H]+ | 149.0592 | cinnamic acid |
| 351200 | 111700 | 1.27 | [M+H]+ | 244.0929 | Cytidine |
| 217000 | 68680 | 1.12 | [M+H]+ | 138.0547 | Trigonelline |
| 1028000 | 313900 | 1.08 | [M+H]+ | 116.0703 | Proline |
| 156000 | 46940 | 0.94 | [M+H]+ | 120.065 | Threonine |
| 77610 | 20780 | 10.47 | [M+H]+ | 387.2527 | Bufalin |
| 200400 | 56280 | 0.95 | [M+H]+ | 148.0601 | Glutamic acid |
| 150800 | 42930 | 15.28 | [M+H]+ | 237.2214 | Ambroxane |
| 131200 | 35020 | 1.32 | [M+H]+ | 147.044 | Coumarin |

Note: POS, positive.

**Table S3. Quantification of drug-containing serum in the LSW group (NEG mode)**

| Area | Height | Retention Time | Adduct / Charge | Found At Mass | Library Hit |
| --- | --- | --- | --- | --- | --- |
| 377900 | 32000 | 19.6 | [M-H]- | 265.1472 | Sambucinol |
| 25330 | 4770 | 18.82 | [M-H]- | 337.3109 | Erucic acid |
| 22010 | 4668 | 18.76 | [M-H]- | 311.295 | ARACHIDATE |
| 22010 | 4668 | 18.76 | [M-H]- | 311.295 | Arachidic acid |
| 13480 | 3483 | 18 | [M-H]- | 297.2793 | Methylstearate |
| 14540 | 3754 | 18 | [M-H]- | 297.2793 | Nonadecanoic acid |
| 67360 | 11200 | 17.71 | [M-H]- | 583.2551 | Bilirubin |
| 51560 | 8601 | 17.71 | [M-H]- | 583.2551 | Bilirubin |
| 2333 | 304 | 17.45 | [M-H]- | 281.249 | Elaidic acid |
| 2333 | 304 | 17.45 | [M-H]- | 281.249 | Elaidic acid |
| 2333 | 304 | 17.45 | [M-H]- | 281.249 | OLEATE |
| 2333 | 304 | 17.45 | [M-H]- | 281.249 | Oleic acid |
| 2333 | 304 | 17.45 | [M-H]- | 281.249 | Vaccenic acid |
| 2076 | 257 | 17.44 | [M-H]- | 281.2477 | Elaidic acid |
| 692200 | 163400 | 17.3 | [M-H]- | 283.2638 | Ethyl hexadecanoate1 |
| 727300 | 171200 | 17.3 | [M-H]- | 283.2638 | STEARATE |
| 727300 | 171200 | 17.3 | [M-H]- | 283.2638 | Stearic acid |
| 15980 | 1934 | 16.97 | [M-H]- | 365.3414 | NERVONATE |
| 15980 | 1934 | 16.97 | [M-H]- | 365.3414 | Nervonic acid |
| 87060 | 26590 | 16.7 | [M-H]- | 269.2478 | HEPTADECANOATE |
| 87060 | 26590 | 16.7 | [M-H]- | 269.2478 | Heptadecanoic acid |
| 82390 | 25210 | 16.7 | [M-H]- | 269.2478 | Isopropyl myristate |
| 1804 | 142 | 16.49 | [M-H]- | 249.1484 | Octisalate |
| 4851 | 324 | 16.46 | [M-H]- | 121.029 | 4-HYDROXYBENZALDEHYDE |
| 11180 | 1175 | 16.44 | [M-H]- | 219.1752 | 2,6-Di-tert-butyl-4-methylphenol |
| 3768 | 505 | 16.42 | [M-H]- | 73.0291 | PROPANOATE |
| 3768 | 505 | 16.42 | [M-H]- | 73.0291 | PROPIONATE |
| 1897000 | 484400 | 16.3 | [M-H]- | 281.2478 | Elaidic acid |
| 1897000 | 484400 | 16.3 | [M-H]- | 281.2478 | Elaidic acid |
| 1799000 | 459700 | 16.3 | [M-H]- | 281.2478 | Elaidic acid |
| 1897000 | 484400 | 16.3 | [M-H]- | 281.2478 | OLEATE |
| 1897000 | 484400 | 16.3 | [M-H]- | 281.2478 | Oleic acid |
| 18390 | 5041 | 16.3 | [M-H]- | 265.1464 | Sambucinol |
| 1897000 | 484400 | 16.3 | [M-H]- | 281.2478 | Vaccenic acid |
| 1877000 | 574000 | 16.15 | [M-H]- | 255.2324 | PALMITATE |
| 231900 | 74770 | 16.13 | [M-H]- | 331.2635 | CP 47,497 C8-homolog Negative Mode |
| 5251 | 1494 | 16.03 | [M-H]- | 293.2482 | Methyllinoleate |
| 3924 | 339 | 15.75 | [M-H]- | 151.04 | Mandelic Acid |
| 3924 | 339 | 15.75 | [M-H]- | 151.04 | Methylparaben |
| 4017 | 351 | 15.72 | [M-H]- | 138.0196 | 4-Nitrophenol |
| 6051 | 391 | 15.72 | [M-H]- | 115.0762 | Butyl Acetate |
| 2999 | 906 | 15.62 | [M-H]- | 241.2536 | HEXADECANOL |
| 59660 | 20600 | 15.62 | [M-H]- | 241.2169 | Pentadecanoic acid |
| 30790 | 11100 | 15.56 | [M-H]- | 329.2204 | 11a-Hydroxyprogesterone |
| 3879000 | 1244000 | 15.56 | [M-H]- | 279.2324 | Linoleic acid |
| 3713000 | 1195000 | 15.56 | [M-H]- | 279.2324 | Linoleic acid |
| 2301000 | 646000 | 15.4 | [M-H]- | 303.2326 | Fatty acid C20:4 |
| 1732 | 266 | 15.39 | [M-H]- | 249.1483 | Octisalate |
| 3685 | 570 | 15.37 | [M-H]- | 233.156 | Lidocaine |
| 1341 | 202 | 15.29 | [M-H]- | 269.244 | HEPTADECANOATE |
| 1341 | 202 | 15.29 | [M-H]- | 269.244 | Heptadecanoic acid |
| 33920 | 6281 | 15.22 | [M-H]- | 265.1461 | Sambucinol |
| 4506 | 1205 | 15.21 | [M-H]- | 283.2634 | Ethyl hexadecanoate1 |
| 3623 | 569 | 15.12 | [M-H]- | 297.046 | Aflatoxin P1 |
| 10490 | 1298 | 15.11 | [M-H]- | 219.1752 | 2,6-Di-tert-butyl-4-methylphenol |
| 3987 | 313 | 15.09 | [M-H]- | 101.0601 | Isovaleric acid-1 |
| 248100 | 80680 | 15.05 | [M-H]- | 227.2013 | Ethyl dodecanoate |
| 257700 | 83860 | 15.05 | [M-H]- | 227.2013 | MYRISTATE |
| 257700 | 83860 | 15.05 | [M-H]- | 227.2013 | Myristic acid |
| 2270 | 273 | 14.97 | [M-H]- | 171.1374 | Capric acid |
| 659300 | 151500 | 14.9 | [M-H]- | 277.2161 | Alpha-Linolenic acid |
| 160600 | 49110 | 14.79 | [M-H]- | 301.2168 | EICOSAPENTAENOATE |
| 6951 | 544 | 14.79 | [M-H]- | 93.0333 | PHENOL |
| 6951 | 544 | 14.79 | [M-H]- | 93.0333 | PHENOL |
| 1237 | 220 | 14.55 | [M-H]- | 165.0542 | Atrolactic acid |
| 1237 | 220 | 14.55 | [M-H]- | 165.0542 | Phenyllactate (Pla) |
| 2648 | 428 | 14.45 | [M-H]- | 115.0747 | Butyl Acetate |
| 5950 | 549 | 14.21 | [M-H]- | 283.2631 | STEARATE |
| 5950 | 549 | 14.21 | [M-H]- | 283.2631 | Stearic acid |
| 3679 | 931 | 13.95 | [M-H]- | 375.2891 | LITHOCHOLATE |
| 3679 | 931 | 13.95 | [M-H]- | 375.2891 | Lithocholic acid |
| 3679 | 931 | 13.95 | [M-H]- | 375.2891 | Lithocholic acid |
| 3024 | 841 | 13.95 | [M-H]- | 375.2891 | Lithocholic acid |
| 30160 | 8865 | 13.89 | [M-H]- | 319.227 | 12(S)HETE |
| 2239 | 258 | 13.82 | [M-H]- | 283.2635 | STEARATE |
| 2239 | 258 | 13.82 | [M-H]- | 283.2635 | Stearic acid |
| 19860 | 6781 | 13.78 | [M-H]- | 200.173 | 11-Aminoundecanoic acid |
| 8422 | 2040 | 13.78 | [M-H]- | 205.1589 | 2,4-Di-tert-butylphenol |
| 179500 | 60490 | 13.78 | [M-H]- | 199.1701 | Dodecanoic acid |
| 179500 | 60490 | 13.78 | [M-H]- | 199.1701 | LAURATE |
| 4411 | 383 | 13.66 | [M-H]- | 121.0285 | 3-HYDROXYBENZALDEHYDE |
| 4411 | 383 | 13.66 | [M-H]- | 121.0285 | 4-HYDROXYBENZALDEHYDE |
| 4411 | 383 | 13.66 | [M-H]- | 121.0285 | BENZOATE |
| 3817 | 596 | 13.56 | [M-H]- | 157.1227 | Ethyl Heptanoate |
| 26880 | 7880 | 13.43 | [M-H]- | 313.2388 | Octadecanedioic acid |
| 907900 | 268800 | 13.36 | [M-H]- | 319.227 | 12(S)HETE |
| 907900 | 268800 | 13.36 | [M-H]- | 319.227 | 5(S)HETE |
| 12460 | 4370 | 13.11 | [M-H]- | 319.2263 | 15(S)HETE |
| 5710 | 973 | 13.11 | [M-H]- | 219.1754 | 2,6-Di-tert-butyl-4-methylphenol |
| 20960 | 1876 | 12.96 | [M-H]- | 482.2947 | LITHOCHOLYLTAURINE |
| 1938 | 186 | 12.72 | [M-H]- | 297.0456 | Aflatoxin P1 |
| 7095 | 1224 | 12.38 | [M-H]- | 281.2488 | Elaidic acid |
| 7095 | 1224 | 12.38 | [M-H]- | 281.2488 | Elaidic acid |
| 6504 | 1130 | 12.38 | [M-H]- | 281.2488 | Elaidic acid |
| 7095 | 1224 | 12.38 | [M-H]- | 281.2488 | OLEATE |
| 7095 | 1224 | 12.38 | [M-H]- | 281.2488 | Oleic acid |
| 7095 | 1224 | 12.38 | [M-H]- | 281.2488 | Vaccenic acid |
| 15930 | 4725 | 12.3 | [M-H]- | 389.2688 | 7a-Hydroxy-3-oxo-5b-cholanoic acid |
| 15930 | 4725 | 12.3 | [M-H]- | 389.2688 | Nutriacholic acid |
| 228600 | 69250 | 12.25 | [M-H]- | 391.2844 | Chenodeoxycholic acid |
| 228600 | 69250 | 12.25 | [M-H]- | 391.2844 | DEOXYCHOLATE |
| 228600 | 69250 | 12.25 | [M-H]- | 391.2844 | Deoxycholic acid |
| 228600 | 69250 | 12.25 | [M-H]- | 391.2844 | Hyodeoxycholic Acid |
| 228600 | 69250 | 12.25 | [M-H]- | 391.2844 | Hyodeoxycholic Acid |
| 205500 | 62480 | 12.25 | [M-H]- | 391.2844 | Hyodeoxycholic Acid |
| 228600 | 69250 | 12.25 | [M-H]- | 391.2844 | URSODEOXYCHOLATE |
| 228600 | 69250 | 12.25 | [M-H]- | 391.2844 | Ursodeoxycholic acid |
| 228600 | 69250 | 12.25 | [M-H]- | 391.2844 | Ursodeoxycholic acid |
| 205500 | 62480 | 12.25 | [M-H]- | 391.2844 | Ursodeoxycholic acid |
| 205500 | 62480 | 12.25 | [M-H]- | 391.2844 | Deoxycholic acid |
| 205500 | 62480 | 12.25 | [M-H]- | 391.2844 | Ursodeoxycholic Acid |
| 205500 | 62480 | 12.25 | [M-H]- | 391.2844 | Hyodeoxycholic acid |
| 205500 | 62480 | 12.25 | [M-H]- | 391.2844 | Chenodeoxycholic acid |
| 30510 | 9717 | 12.24 | [M-H]- | 285.2069 | Hexadecanedioic acid |
| 46480 | 13840 | 11.7 | [M-H]- | 215.1652 | 3-Hydroxydodecanoic acid |
| 46480 | 13840 | 11.7 | [M-H]- | 215.1652 | 3-Hydroxydodecanoic acid |
| 44590 | 13430 | 11.7 | [M-H]- | 215.1652 | 3-Hydroxydodecanoic acid |
| 4506 | 1357 | 11.66 | [M-H]- | 169.123 | Linalool oxide |
| 29580 | 8569 | 11.65 | [M-H]- | 194.0821 | L- tyrosine methyl ester |
| 6594 | 1091 | 11.64 | [M-H]- | 285.2063 | Hexadecanedioic acid |
| 6275 | 1748 | 11.56 | [M-H]- | 157.1232 | Butyl 3-methylbutanoate |
| 6275 | 1748 | 11.56 | [M-H]- | 157.1232 | Ethyl Heptanoate |
| 6275 | 1748 | 11.56 | [M-H]- | 157.1232 | PROPYL HEXANOATE |
| 34190 | 6914 | 11.33 | [M-H]- | 391.284 | Chenodeoxycholic acid |
| 34190 | 6914 | 11.33 | [M-H]- | 391.284 | Deoxycholic acid |
| 34190 | 6914 | 11.33 | [M-H]- | 391.284 | Hyodeoxycholic Acid |
| 34190 | 6914 | 11.33 | [M-H]- | 391.284 | Hyodeoxycholic Acid |
| 30110 | 6113 | 11.33 | [M-H]- | 391.284 | Hyodeoxycholic Acid |
| 34190 | 6914 | 11.33 | [M-H]- | 391.284 | URSODEOXYCHOLATE |
| 34190 | 6914 | 11.33 | [M-H]- | 391.284 | Ursodeoxycholic acid |
| 34190 | 6914 | 11.33 | [M-H]- | 391.284 | Ursodeoxycholic acid |
| 30110 | 6113 | 11.33 | [M-H]- | 391.284 | Ursodeoxycholic acid |
| 30110 | 6113 | 11.33 | [M-H]- | 391.284 | Deoxycholic acid |
| 30110 | 6113 | 11.33 | [M-H]- | 391.284 | Ursodeoxycholic Acid |
| 30110 | 6113 | 11.33 | [M-H]- | 391.284 | Hyodeoxycholic acid |
| 30110 | 6113 | 11.33 | [M-H]- | 391.284 | Chenodeoxycholic acid |
| 26660 | 7211 | 11.14 | [M-H]- | 389.2681 | 7a-Hydroxy-3-oxo-5b-cholanoic acid |
| 26660 | 7211 | 11.14 | [M-H]- | 389.2681 | Nutriacholic acid |
| 796 | 122 | 11.08 | [M-H]- | 329.0015 | Pyrroloquinoline Quinone |
| 25170 | 7561 | 11.04 | [M-H]- | 257.1749 | Tetradecanedioic acid |
| 20670 | 6165 | 10.98 | [M-H]- | 448.3055 | GLYCOCHENODEOXYCHOLATE |
| 17170 | 5174 | 10.98 | [M-H]- | 448.3055 | Glycoursodeoxycholic acid |
| 269800 | 38430 | 10.92 | [M-H]- | 498.2881 | Taurodeoxycholic acid |
| 732 | 296 | 10.9 | [M-H]- | 437.2158 | Blinin +HCOOH |
| 317800 | 89530 | 10.89 | [M-H]- | 391.2844 | Chenodeoxycholic acid |
| 317800 | 89530 | 10.89 | [M-H]- | 391.2844 | Deoxycholic acid |
| 317800 | 89530 | 10.89 | [M-H]- | 391.2844 | Hyodeoxycholic Acid |
| 317800 | 89530 | 10.89 | [M-H]- | 391.2844 | Hyodeoxycholic Acid |
| 317800 | 89530 | 10.89 | [M-H]- | 391.2844 | URSODEOXYCHOLATE |
| 317800 | 89530 | 10.89 | [M-H]- | 391.2844 | Ursodeoxycholic acid |
| 317800 | 89530 | 10.89 | [M-H]- | 391.2844 | Ursodeoxycholic acid |
| 279700 | 79690 | 10.88 | [M-H]- | 391.2844 | Hyodeoxycholic Acid |
| 279700 | 79690 | 10.88 | [M-H]- | 391.2844 | Ursodeoxycholic acid |
| 279700 | 79690 | 10.88 | [M-H]- | 391.2844 | Ursodeoxycholic Acid |
| 279700 | 79690 | 10.88 | [M-H]- | 391.2844 | Hyodeoxycholic acid |
| 279700 | 79690 | 10.88 | [M-H]- | 391.2844 | Chenodeoxycholic acid |
| 11600 | 3383 | 10.66 | [M-H]- | 581.2407 | BILIVERDIN |
| 661700 | 184500 | 10.63 | [M-H]- | 407.2794 | Cholic acid |
| 584700 | 163400 | 10.63 | [M-H]- | 407.2794 | Cholan-24-oic acid |
| 18080 | 3065 | 10.57 | [M-H]- | 405.2627 | 3-Oxocholic acid |
| 327100 | 93620 | 10.43 | [M-H]- | 243.1601 | 1,11-Undecanedicarboxylic acid |
| 13080 | 3157 | 10.08 | [M-H]- | 215.1651 | 12-Hydroxydodecanoic acid |
| 13080 | 3157 | 10.08 | [M-H]- | 215.1651 | 3-Hydroxydodecanoic acid |
| 13080 | 3157 | 10.08 | [M-H]- | 215.1651 | 3-Hydroxydodecanoic acid |
| 12460 | 3082 | 10.08 | [M-H]- | 215.1651 | 3-Hydroxydodecanoic acid |
| 13080 | 3157 | 10.08 | [M-H]- | 215.1651 | OMEGA-HYDROXYDODECANOATE |
| 64120 | 16380 | 10.07 | [M-H]- | 407.2791 | Cholic acid |
| 8817 | 1190 | 9.83 | [M-H]- | 341.0045 | FPePA (3-Perfluoropentyl propanoic acid) (neg) |
| 35870 | 4282 | 9.82 | [M-H]- | 498.2894 | Taurodeoxycholic acid |
| 89820 | 28480 | 9.8 | [M-H]- | 229.1446 | Dodecanedioic acid |
| 89820 | 28480 | 9.8 | [M-H]- | 229.1446 | Dodecanedioic acid |
| 89820 | 28480 | 9.8 | [M-H]- | 229.1446 | Dodecanedioic acid |
| 85900 | 27350 | 9.8 | [M-H]- | 229.1446 | Dodecanedioic acid |
| 628 | 127 | 9.73 | [M-H]- | 453.1424 | Nodakenin +HCOOH |
| 10780 | 2802 | 9.69 | [M-H]- | 351.217 | Prostaglandin D2 |
| 10780 | 2802 | 9.69 | [M-H]- | 351.217 | Prostaglandin E2 |
| 3416 | 516 | 9.45 | [M-H]- | 187.0975 | Azelaic acid |
| 3416 | 516 | 9.45 | [M-H]- | 187.0975 | AZELATE |
| 897600 | 194800 | 9.39 | [M-H]- | 498.2878 | Taurodeoxycholic acid |
| 46440 | 7659 | 9.38 | [M-H]- | 369.2277 | Thromboxane B2 |
| 14460 | 3150 | 9.37 | [M-H]- | 227.1283 | Traumatic acid |
| 22080 | 6572 | 9.36 | [M-H]- | 775.6781 | THYROXINE |
| 16060 | 4745 | 9.32 | [M-H]- | 407.2786 | Cholic acid |
| 109300 | 30980 | 9.16 | [M-H]- | 215.129 | Undecanedioic acid |
| 3228 | 548 | 9.13 | [M-H]- | 151.04 | 3-Cresotinic acid |
| 3228 | 548 | 9.13 | [M-H]- | 151.04 | 3-HYDROXYPHENYLACETATE |
| 3228 | 548 | 9.13 | [M-H]- | 151.04 | 4-Hydroxy-3-methylbenzoic acid |
| 3320 | 517 | 9.13 | [M-H]- | 151.0397 | Anisic acid |
| 3228 | 548 | 9.13 | [M-H]- | 151.04 | MANDELATE |
| 3320 | 517 | 9.13 | [M-H]- | 151.0397 | p-Anisic acid |
| 3228 | 548 | 9.13 | [M-H]- | 151.04 | p-Anisic acid |
| 3228 | 548 | 9.13 | [M-H]- | 151.04 | P-HYDROXYPHENYLACETATE |
| 3320 | 517 | 9.13 | [M-H]- | 151.0397 | 3-Methoxybenzoic acid |
| 16550 | 3302 | 9.06 | [M-H]- | 464.3002 | GLYCOCHOLATE |
| 16550 | 3302 | 9.06 | [M-H]- | 464.3002 | Glycocholic acid |
| 6551 | 1058 | 9.05 | [M-H]- | 182.9883 | Chelidonic acid |
| 3970 | 719 | 9.05 | [M-H]- | 465.3016 | Cholesterol sulfate |
| 20120 | 5970 | 9.05 | [M-H]- | 165.0557 | Ethylparaben |
| 11770 | 2311 | 9.05 | [M-H]- | 464.3002 | Glycocholic acid |
| 20120 | 5970 | 9.05 | [M-H]- | 165.0557 | Ethyl 4-hydroxybenzoate |
| 5864 | 1684 | 8.98 | [M-H]- | 159.1028 | 2-Hydroxyoctanoic acid |
| 6046 | 1742 | 8.98 | [M-H]- | 159.1028 | 8-Hydroxyoctanoic acid |
| 55540 | 13980 | 8.94 | [M-H]- | 265.1449 | Sambucinol |
| 5455 | 1513 | 8.81 | [M-H]- | 149.0607 | 3-Phenylpropionic acid |
| 473000 | 151200 | 8.73 | [M-H]- | 188.0718 | 3-Indolepropionic acid |
| 364300 | 49550 | 8.54 | [M-H]- | 514.2832 | Taurocholic acid |
| 1481 | 200 | 8.48 | [M-H]- | 168.0302 | 2-Furoylglycine |
| 146900 | 46140 | 8.48 | [M-H]- | 186.0562 | Indoleacrylic acid |
| 123900 | 29730 | 8.48 | [M-H]- | 201.1134 | SEBACATE |
| 123900 | 29730 | 8.48 | [M-H]- | 201.1134 | Sebacic acid |
| 24310 | 3741 | 8.23 | [M-H]- | 138.0197 | 4-Nitrophenol |
| 5224 | 560 | 8.12 | [M-H]- | 89.0243 | GLYCERALDEHYDE |
| 5224 | 560 | 8.12 | [M-H]- | 89.0243 | LACTATE |
| 8569 | 1522 | 8.1 | [M-H]- | 137.0244 | 4-HYDROXYBENZOATE |
| 8569 | 1522 | 8.1 | [M-H]- | 137.0244 | 4-Hydroxybenzoic acid |
| 8173 | 1484 | 8.1 | [M-H]- | 137.0244 | Salicyclic Acid |
| 8569 | 1522 | 8.1 | [M-H]- | 137.0244 | Salicylic acid |
| 8173 | 1484 | 8.1 | [M-H]- | 137.0244 | salicylic acid |
| 11760 | 2080 | 8.07 | [M-H]- | 185.1183 | 10-Hydroxy-2-decenoic acid |
| 11760 | 2080 | 8.07 | [M-H]- | 185.1183 | 10-hydroxydec-2-enoic acid |
| 894 | 122 | 7.89 | [M-H]- | 182.9564 | PFESi (perfluoroethane sulfinate) (neg) |
| 9232 | 688 | 7.87 | [M-H]- | 112.9854 | PFEA (perfluoro-n-ethanoic acid) (neg) |
| 603000 | 173600 | 7.78 | [M-H]- | 187.0975 | Azelaic acid |
| 603000 | 173600 | 7.78 | [M-H]- | 187.0975 | AZELATE |
| 10510 | 2925 | 7.65 | [M-H]- | 583.255 | Bilirubin |
| 6159 | 1710 | 7.65 | [M-H]- | 160.0402 | Indole-3-carboxylic acid |
| 5299 | 1589 | 7.65 | [M-H]- | 160.0402 | Indole-5-Carboxylic Acid |
| 12060 | 1590 | 7.55 | [M-H]- | 131.0712 | 2-Hydroxyisocaproic Acid |
| 76920 | 24000 | 7.44 | [M-H]- | 245.0933 | N-Acetyl-L-tryptophan |
| 71740 | 22340 | 7.43 | [M-H]- | 204.0666 | Indolelactic acid |
| 71740 | 22340 | 7.43 | [M-H]- | 204.0666 | Indolelactic acid |
| 68240 | 21470 | 7.43 | [M-H]- | 204.0666 | Indolelactic acid |
| 990 | 182 | 7.38 | [M-H]- | 182.9585 | PFESi (perfluoroethane sulfinate) (neg) |
| 11590 | 2121 | 7.24 | [M-H]- | 179.0351 | 3-(4-HYDROXYPHENYL)PYRUVATE |
| 11590 | 2121 | 7.24 | [M-H]- | 179.0351 | CAFFEATE |
| 4999 | 1687 | 7.24 | [M-H]- | 164.0718 | L-Phenylalanine |
| 100500 | 32090 | 7.24 | [M-H]- | 206.0823 | N-Acetyl-L-phenylalanine |
| 100500 | 32090 | 7.24 | [M-H]- | 206.0823 | N-ACETYLPHENYLALANINE |
| 4999 | 1687 | 7.24 | [M-H]- | 164.0718 | PHENYLALANINE |
| 4755 | 1590 | 7.24 | [M-H]- | 164.0718 | Phenprobamate |
| 4755 | 1590 | 7.24 | [M-H]- | 164.0718 | Phenylalanine |
| 8069 | 598 | 7.22 | [M-H]- | 89.0248 | DL-Glyceraldehyde |
| 8294 | 605 | 7.21 | [M-H]- | 89.0248 | GLYCERALDEHYDE |
| 8294 | 605 | 7.21 | [M-H]- | 89.0248 | LACTATE |
| 3512 | 713 | 7.21 | [M-H]- | 147.0448 | cinnamic acid |
| 4197 | 1029 | 7.13 | [M-H]- | 107.0511 | m-Cresol |
| 108700 | 25140 | 7.06 | [M-H]- | 173.0819 | SUBERATE |
| 108700 | 25140 | 7.06 | [M-H]- | 173.0819 | Suberic acid |
| 910 | 211 | 6.99 | [M-H]- | 144.1059 | DEOXYCARNITINE |
| 652 | 211 | 6.99 | [M-H]- | 144.1059 | N-Methyl-L-leucine hydrochloride |
| 13060 | 4010 | 6.93 | [M-H]- | 144.0457 | 4-Hydroxyquinoline |
| 60030 | 17750 | 6.92 | [M-H]- | 190.051 | 5-HYDROXYINDOLEACETATE |
| 12580 | 3712 | 6.91 | [M-H]- | 157.0871 | 2-Oxooctanoic acid |
| 65650 | 19510 | 6.9 | [M-H]- | 172.098 | N-ACETYLLEUCINE |
| 63390 | 18990 | 6.9 | [M-H]- | 172.098 | N-Acetyl-L-leucine |
| 330900 | 98720 | 6.78 | [M-H]- | 192.0667 | Phenylacetylglycine |
| 1858 | 320 | 6.77 | [M-H]- | 163.0392 | p-Coumaric acid |
| 20260 | 6842 | 6.66 | [M-H]- | 581.2442 | BILIVERDIN |
| 11900 | 3171 | 6.52 | [M-H]- | 243.0811 | Biotin_ |
| 749200 | 100900 | 6.44 | [M-H]- | 129.0558 | 2-Methyl-3-ketovaleric acid |
| 749200 | 100900 | 6.44 | [M-H]- | 129.0558 | Ketoleucine |
| 749200 | 100900 | 6.44 | [M-H]- | 129.0558 | MEVALOLACTONE |
| 740700 | 99730 | 6.44 | [M-H]- | 129.0558 | MEVALOLACTONE |
| 4743 | 571 | 6.43 | [M-H]- | 131.0386 | GLUTARATE |
| 111400 | 31070 | 6.39 | [M-H]- | 383.191 | (±)-Isorhynchophylline |
| 1110000 | 256600 | 6.38 | [M-H]- | 212.0023 | INDOXYL SULFATE |
| 7717 | 2247 | 6.33 | [M-H]- | 151.0402 | 3-Cresotinic acid |
| 7717 | 2247 | 6.33 | [M-H]- | 151.0402 | 3-HYDROXYPHENYLACETATE |
| 7717 | 2247 | 6.33 | [M-H]- | 151.0402 | 4-Hydroxy-3-methylbenzoic acid |
| 7717 | 2247 | 6.33 | [M-H]- | 151.0402 | P-HYDROXYPHENYLACETATE |
| 49260 | 12140 | 6.32 | [M-H]- | 159.0662 | 3-Methyladipic acid |
| 49260 | 12140 | 6.32 | [M-H]- | 159.0662 | 6-CARBOXYHEXANOATE |
| 49260 | 12140 | 6.32 | [M-H]- | 159.0662 | Pimelic acid |
| 28670 | 2481 | 6.31 | [M-H]- | 121.0295 | 3-HYDROXYBENZALDEHYDE |
| 28670 | 2481 | 6.31 | [M-H]- | 121.0295 | 4-HYDROXYBENZALDEHYDE |
| 28670 | 2481 | 6.31 | [M-H]- | 121.0295 | BENZOATE |
| 1633 | 188 | 6.3 | [M-H]- | 190.08 | 5-Methoxytryptophol |
| 11420 | 1202 | 6.29 | [M-H]- | 166.0225 | QUINOLINATE |
| 11420 | 1202 | 6.29 | [M-H]- | 166.0225 | Quinolinic acid |
| 3666 | 448 | 6.29 | [M-H]- | 383.0985 | S-ADENOSYLHOMOCYSTEINE |
| 83530 | 12910 | 6.28 | [M-H]- | 165.0192 | Phthalic Acid_ |
| 82000 | 12670 | 6.28 | [M-H]- | 165.0192 | Phthalic Acid_ |
| 82000 | 12670 | 6.28 | [M-H]- | 165.0192 | Phthalic Acid_ |
| 83530 | 12910 | 6.28 | [M-H]- | 165.0192 | Terephthalic Acid |
| 6998 | 654 | 6.27 | [M-H]- | 89.024 | DL-Glyceraldehyde |
| 7158 | 679 | 6.27 | [M-H]- | 89.024 | GLYCERALDEHYDE |
| 7158 | 679 | 6.27 | [M-H]- | 89.024 | LACTATE |
| 3451 | 590 | 6.2 | [M-H]- | 137.025 | 3-Hydroxybenzoic acid |
| 3451 | 590 | 6.2 | [M-H]- | 137.025 | 4-HYDROXYBENZOATE |
| 3451 | 590 | 6.2 | [M-H]- | 137.025 | 4-Hydroxybenzoic acid |
| 2968 | 553 | 6.2 | [M-H]- | 137.0244 | Salicyclic Acid |
| 3451 | 590 | 6.2 | [M-H]- | 137.025 | SALICYLATE |
| 3451 | 590 | 6.2 | [M-H]- | 137.025 | Salicylic acid |
| 2968 | 553 | 6.2 | [M-H]- | 137.0244 | 4-Hydroxybenzoic acid |
| 2968 | 553 | 6.2 | [M-H]- | 137.0244 | salicylic acid |
| 4055 | 951 | 6.15 | [M-H]- | 455.096 | Flavin Mononucleotide |
| 4105 | 515 | 6.1 | [M-H]- | 175.024 | Vitamin C |
| 3948 | 1044 | 6.08 | [M-H]- | 87.0441 | ISOBUTYRATE |
| 1995 | 180 | 6.08 | [M-H]- | 117.0187 | METHYLMALONATE |
| 1995 | 180 | 6.08 | [M-H]- | 117.0187 | SUCCINATE |
| 1767 | 165 | 6.07 | [M-H]- | 117.0176 | D-Erythronolactone |
| 5730 | 1134 | 6.02 | [M-H]- | 144.0451 | 4-Hydroxyquinoline |
| 17420 | 3456 | 6.02 | [M-H]- | 188.0352 | KYNURENATE |
| 84830 | 15140 | 5.98 | [M-H]- | 158.0821 | Isovalerylglycine |
| 1110 | 117 | 5.98 | [M-H]- | 173.0441 | Shikimic acid |
| 244400 | 43000 | 5.77 | [M-H]- | 181.0505 | HYDROXYPHENYLLACTATE |
| 244400 | 43000 | 5.77 | [M-H]- | 181.0505 | Hydroxyphenyllactic acid |
| 5289 | 775 | 5.75 | [M-H]- | 241.118 | Diphenylcarbazide |
| 3690000 | 796900 | 5.66 | [M-H]- | 203.0828 | L-Tryptophan |
| 126100 | 20500 | 5.66 | [M-H]- | 176.0387 | N-Formyl-L-methionine_ |
| 122800 | 19650 | 5.66 | [M-H]- | 176.0387 | N-Formyl-L-methionine_ |
| 3596000 | 775500 | 5.66 | [M-H]- | 203.0828 | L-Tryptophan |
| 5852 | 724 | 5.61 | [M-H]- | 131.0711 | 2-Hydroxyisocaproic Acid |
| 50170 | 6622 | 5.6 | [M-H]- | 145.0507 | 2-METHYLGLUTARATE |
| 50170 | 6622 | 5.6 | [M-H]- | 145.0507 | ADIPATE |
| 2773 | 443 | 5.49 | [M-H]- | 194.0454 | o-Hydroxyhippuric acid |
| 10130 | 1623 | 5.46 | [M-H]- | 212.0024 | INDOXYL SULFATE |
| 2349 | 304 | 5.41 | [M-H]- | 179.0346 | 3-(4-HYDROXYPHENYL)PYRUVATE |
| 2349 | 304 | 5.41 | [M-H]- | 179.0346 | CAFFEATE |
| 1252 | 180 | 5.41 | [M-H]- | 97.0647 | Trans-2-Hexen-1-Al |
| 849200 | 155400 | 5.33 | [M-H]- | 218.1036 | PANTOTHENATE |
| 849200 | 155400 | 5.33 | [M-H]- | 218.1036 | Pantothenic acid |
| 99350 | 32040 | 5.21 | [M-H]- | 241.0826 | Thymidine-1 |
| 8758 | 1401 | 5.19 | [M-H]- | 131.0345 | 2-Methylsuccinic Acid |
| 8837 | 1423 | 5.19 | [M-H]- | 131.0345 | Dimethylmalonic acid |
| 8837 | 1423 | 5.19 | [M-H]- | 131.0345 | ETHYLMALONATE |
| 8837 | 1423 | 5.19 | [M-H]- | 131.0345 | GLUTARATE |
| 8837 | 1423 | 5.19 | [M-H]- | 131.0345 | Glutaric acid |
| 8837 | 1423 | 5.19 | [M-H]- | 131.0345 | Methylsuccinic acid |
| 1385000 | 327400 | 5.13 | [M-H]- | 164.0717 | L-Phenylalanine |
| 1385000 | 327400 | 5.13 | [M-H]- | 164.0717 | PHENYLALANINE |
| 10290 | 1931 | 5.13 | [M-H]- | 125.0353 | THYMINE |
| 9777 | 1912 | 5.13 | [M-H]- | 125.0353 | Thymine-1 |
| 1363000 | 322800 | 5.13 | [M-H]- | 164.0717 | Phenprobamate |
| 1363000 | 322800 | 5.13 | [M-H]- | 164.0717 | Phenylalanine |
| 45700 | 10080 | 5.09 | [M-H]- | 190.0508 | 5-HYDROXYINDOLEACETATE |
| 43730 | 10880 | 5.09 | [M-H]- | 207.0775 | KYNURENINE |
| 1000 | 194 | 5.08 | [M-H]- | 117.0197 | Acetoxyacetic Acid |
| 1000 | 194 | 5.08 | [M-H]- | 117.0197 | D-Erythronolactone |
| 16940 | 5157 | 5.06 | [M-H]- | 181.0365 | 1-Methyluric Acid |
| 159900 | 47180 | 4.97 | [M-H]- | 283.0681 | Xanthosine |
| 17070 | 3961 | 4.96 | [M-H]- | 182.0457 | 4-PYRIDOXATE |
| 20530 | 4381 | 4.83 | [M-H]- | 227.0676 | DEOXYURIDINE |
| 12580 | 1441 | 4.58 | [M-H]- | 115.0401 | METHYL ACETOACETATE |
| 77080 | 8352 | 3.28 | [M-H]- | 243.0624 | Uridine |
| 73840 | 8030 | 3.28 | [M-H]- | 243.0624 | Uridine |
| 129200 | 11100 | 3.22 | [M-H]- | 103.0401 | (S)-2-Hydroxybutanoicacid |
| 130300 | 11150 | 3.22 | [M-H]- | 103.0401 | 2-HYDROXYBUTYRATE |
| 14010 | 1594 | 3.04 | [M-H]- | 163.0402 | 4-COUMARATE |
| 659200 | 75290 | 3.04 | [M-H]- | 180.0666 | L-Tyrosine |
| 659200 | 75290 | 3.04 | [M-H]- | 180.0666 | o-Tyrosine |
| 659200 | 75290 | 3.04 | [M-H]- | 180.0666 | TYROSINE |
| 60060 | 6296 | 2.29 | [M-H]- | 128.0353 | OXOPROLINE |
| 60060 | 6296 | 2.29 | [M-H]- | 128.0353 | PYROGLUTAMATE |
| 60060 | 6296 | 2.29 | [M-H]- | 128.0353 | Pyroglutamic acid |
| 60060 | 6296 | 2.29 | [M-H]- | 128.0353 | Pyrrolidonecarboxylic acid |
| 876800 | 108900 | 2.29 | [M-H]- | 167.0212 | URATE |
| 50810 | 7613 | 2.15 | [M-H]- | 226.0835 | DEOXYCYTIDINE |
| 201300 | 21960 | 2.08 | [M-H]- | 191.0199 | CITRATE |
| 196300 | 21420 | 2.08 | [M-H]- | 191.0199 | CITRATE |
| 201300 | 21960 | 2.08 | [M-H]- | 191.0199 | Citric acid |
| 196300 | 21420 | 2.08 | [M-H]- | 191.0199 | Citric acid |
| 4827 | 715 | 1.96 | [M-H]- | 173.0092 | Dehydroascorbic acid |
| 26570 | 2230 | 1.87 | [M-H]- | 161.0454 | 3-HYDROXYMETHYLGLUTARATE |
| 763500 | 75210 | 1.85 | [M-H]- | 103.0401 | (R)-3-Hydroxybutyric acid |
| 763500 | 75210 | 1.85 | [M-H]- | 103.0401 | 3-HYDROXYBUTANOATE |
| 763500 | 75210 | 1.85 | [M-H]- | 103.0401 | 3-Hydroxybutyric acid |
| 759400 | 74760 | 1.85 | [M-H]- | 103.0401 | R-3-Hydroxybutyric acid |
| 12850 | 1295 | 1.81 | [M-H]- | 130.0509 | CIS-4-HYDROXY-D-PROLINE |
| 12850 | 1295 | 1.81 | [M-H]- | 130.0509 | N-ACETYLALANINE |
| 12850 | 1295 | 1.81 | [M-H]- | 130.0509 | N-Acetyl-L-alanine |
| 16670 | 2702 | 1.74 | [M-H]- | 148.0436 | METHIONINE |
| 122600 | 19300 | 1.72 | [M-H]- | 130.0873 | 6-Aminocaproic Acid |
| 122600 | 19300 | 1.72 | [M-H]- | 130.0873 | Alloisoleucine |
| 122600 | 19300 | 1.72 | [M-H]- | 130.0873 | Aminocaproic acid |
| 122600 | 19300 | 1.72 | [M-H]- | 130.0873 | Beta-Leucine |
| 122600 | 19300 | 1.72 | [M-H]- | 130.0873 | ISOLEUCINE |
| 122600 | 19300 | 1.72 | [M-H]- | 130.0873 | L-Alloisoleucine |
| 122600 | 19300 | 1.72 | [M-H]- | 130.0873 | LEUCINE |
| 122600 | 19300 | 1.72 | [M-H]- | 130.0873 | L-Isoleucine |
| 122600 | 19300 | 1.72 | [M-H]- | 130.0873 | L-Leucine |
| 122600 | 19300 | 1.72 | [M-H]- | 130.0873 | L-Norleucine |
| 122600 | 19300 | 1.72 | [M-H]- | 130.0873 | NORLEUCINE |
| 120800 | 19060 | 1.72 | [M-H]- | 130.0873 | Isoleucine |
| 63850 | 7550 | 1.63 | [M-H]- | 117.0194 | D-Erythronolactone |
| 64060 | 7637 | 1.63 | [M-H]- | 117.0194 | METHYLMALONATE |
| 64060 | 7637 | 1.63 | [M-H]- | 117.0194 | Methylmalonic acid |
| 64060 | 7637 | 1.63 | [M-H]- | 117.0194 | SUCCINATE |
| 64060 | 7637 | 1.63 | [M-H]- | 117.0194 | Succinic acid |
| 63850 | 7550 | 1.63 | [M-H]- | 117.0194 | Amber Acid |
| 63850 | 7550 | 1.63 | [M-H]- | 117.0194 | Succinic acid |
| 1723000 | 93440 | 1.52 | [M-H]- | 89.0244 | DL-Glyceraldehyde |
| 1728000 | 93700 | 1.52 | [M-H]- | 89.0244 | GLYCERALDEHYDE |
| 1728000 | 93700 | 1.52 | [M-H]- | 89.0244 | LACTATE |
| 89600 | 15310 | 1.41 | [M-H]- | 173.0092 | cis-Aconitic acid |
| 91750 | 15600 | 1.41 | [M-H]- | 173.0092 | trans-Aconitic acid |
| 66430 | 12890 | 1.34 | [M-H]- | 112.9855 | PFEA (perfluoro-n-ethanoic acid) (neg) |
| 309500 | 96000 | 1.33 | [M-H]- | 180.0667 | L-Tyrosine |
| 27900 | 6430 | 1.33 | [M-H]- | 188.0565 | N-ACETYLGLUTAMATE |
| 27900 | 6430 | 1.33 | [M-H]- | 188.0565 | N-Acetylglutamic acid |
| 309500 | 96000 | 1.33 | [M-H]- | 180.0667 | o-Tyrosine |
| 309500 | 96000 | 1.33 | [M-H]- | 180.0667 | TYROSINE |
| 9139 | 2327 | 1.32 | [M-H]- | 163.0395 | 4-COUMARATE |
| 9139 | 2327 | 1.32 | [M-H]- | 163.0395 | PHENYLPYRUVATE |
| 6632 | 2023 | 1.32 | [M-H]- | 163.0395 | Phenylpyruvic Acid |
| 3886 | 622 | 1.29 | [M-H]- | 88.9878 | OXALATE |
| 3886 | 622 | 1.29 | [M-H]- | 88.9878 | OXALATE |
| 50440 | 16900 | 1.28 | [M-H]- | 128.0352 | OXOPROLINE |
| 50440 | 16900 | 1.28 | [M-H]- | 128.0352 | PYROGLUTAMATE |
| 50440 | 16900 | 1.28 | [M-H]- | 128.0352 | Pyroglutamic acid |
| 50440 | 16900 | 1.28 | [M-H]- | 128.0352 | Pyrrolidonecarboxylic acid |
| 559 | 236 | 1.27 | [M-H]- | 128.0725 | 1-Aminocyclopentanecarboxylic acid |
| 902700 | 270600 | 1.27 | [M-H]- | 191.0199 | CITRATE |
| 881300 | 264600 | 1.27 | [M-H]- | 191.0199 | CITRATE |
| 902700 | 270600 | 1.27 | [M-H]- | 191.0199 | Citric acid |
| 986900 | 296300 | 1.27 | [M-H]- | 167.0212 | URATE |
| 559 | 236 | 1.27 | [M-H]- | 128.0725 | Pipecolinic acid |
| 881300 | 264600 | 1.27 | [M-H]- | 191.0199 | Citric acid |
| 347800 | 97590 | 1.26 | [M-H]- | 175.0249 | ASCORBATE |
| 347800 | 97590 | 1.26 | [M-H]- | 175.0249 | Ascorbic acid |
| 14580 | 3775 | 1.26 | [M-H]- | 173.0568 | N-ACETYLASPARAGINE |
| 336900 | 95160 | 1.26 | [M-H]- | 175.0249 | Vitamin C |
| 131300 | 44580 | 1.24 | [M-H]- | 133.014 | L-Malic acid |
| 129600 | 44020 | 1.24 | [M-H]- | 133.014 | L-Malic acid |
| 131300 | 44580 | 1.24 | [M-H]- | 133.014 | MALATE |
| 131300 | 44580 | 1.24 | [M-H]- | 133.014 | Malic acid |
| 50390 | 14810 | 1.06 | [M-H]- | 130.0621 | 3-Guanidinopropionic acid |
| 50390 | 14810 | 1.06 | [M-H]- | 130.0621 | CREATINE |
| 37810 | 10760 | 1.06 | [M-H]- | 105.0192 | GLYCERATE |
| 37810 | 10760 | 1.06 | [M-H]- | 105.0192 | Glyceric acid |
| 1418 | 344 | 1.03 | [M-H]- | 127.0521 | 5,6-Dihydro-5-Methyluracil |
| 54570 | 13460 | 1.02 | [M-H]- | 308.0983 | N-ACETYLNEURAMINATE |
| 54570 | 13460 | 1.02 | [M-H]- | 308.0983 | N-Acetylneuraminic acid |
| 192500 | 59080 | 0.97 | [M-H]- | 174.0884 | Citrulline |
| 186900 | 57480 | 0.97 | [M-H]- | 174.0884 | Citrulline |
| 88050 | 20000 | 0.95 | [M-H]- | 146.0457 | GLUTAMATE |
| 78310 | 18210 | 0.95 | [M-H]- | 146.0457 | Isoglutamic acid |
| 88050 | 20000 | 0.95 | [M-H]- | 146.0457 | L-Glutamic acid |
| 5205 | 1545 | 0.95 | [M-H]- | 187.1204 | L-Homoarginine |
| 78310 | 18210 | 0.95 | [M-H]- | 146.0457 | threo-β-Methylaspartic acid |
| 78310 | 18210 | 0.95 | [M-H]- | 146.0457 | Glutamic acid |
| 5084 | 1745 | 0.93 | [M-H]- | 127.0507 | 5,6-Dihydro-5-Methyluracil |
| 52000 | 13390 | 0.93 | [M-H]- | 124.0074 | TAURINE |
| 51600 | 13320 | 0.93 | [M-H]- | 124.0074 | Taurine |
| 142400 | 44300 | 0.92 | [M-H]- | 145.0618 | GLUTAMINE |
| 27530 | 10000 | 0.91 | [M-H]- | 154.0621 | HISTIDINE |
| 27160 | 9751 | 0.91 | [M-H]- | 154.0621 | Histidine |

Note: NEG, negative; LSW, liushenwan.

**Table 4. Quantification of drug-containing serum in the LSW group (POS mode)**

| **Area** | **Height** | **Retention Time** | **Adduct / Charge** | **Found At Mass** | **Library Hit** |
| --- | --- | --- | --- | --- | --- |
| 33420 | 2305 | 19.89 | [M+H]+ | 109.076 | 1,3-Phenylenediamine |
| 1586 | 272 | 19.62 | [M+H]+ | 180.0392 | S-CARBOXYMETHYLCYSTEINE |
| 16380 | 1965 | 19.57 | [M+H]+ | 217.0537 | 6-Aminopenicillanic acid |
| 41630 | 15150 | 19.48 | [M+H]+ | 122.0964 | 2,4-Dimethylaniline |
| 41630 | 15150 | 19.48 | [M+H]+ | 122.0964 | 2,6-Dimethylaniline |
| 43390 | 16880 | 19.48 | [M+H]+ | 194.1181 | BDB |
| 41630 | 15150 | 19.48 | [M+H]+ | 122.0964 | N,N-Dimethylaniline |
| 42760 | 15270 | 19.48 | [M+H]+ | 122.0964 | PHENETHYLAMINE |
| 8226 | 4010 | 19.47 | [M+H]+ | 118.0861 | BETAINE |
| 8226 | 4010 | 19.47 | [M+H]+ | 118.0861 | N-Methyl-a-aminoisobutyric acid |
| 8003 | 3923 | 19.47 | [M+H]+ | 118.0861 | Betaine |
| 2380 | 467 | 19.43 | [M+H]+ | 135.1169 | p-Cymene |
| 22400 | 3366 | 19.41 | [M+H]+ | 115.0502 | DIHYDROURACIL |
| 159800 | 24060 | 17.71 | [M+H]+ | 585.2724 | BILIRUBIN |
| 50710 | 10220 | 17.48 | [M+H]+ | 391.2856 | BIS(2-ETHYLHEXYL)PHTHALATE |
| 71160 | 19940 | 16.85 | [M+H]+ | 284.2958 | Octadecanamide |
| 21210 | 6132 | 16.15 | [M+H]+ | 257.2486 | PALMITATE |
| 5410 | 1540 | 16.15 | [M+H]+ | 239.2383 | Muscone |
| 379400 | 106300 | 15.56 | [M+H]+ | 281.2482 | LINOLEATE |
| 41130 | 11670 | 15.53 | [M+H]+ | 269.2271 | Epimetendiol |
| 27670 | 6409 | 15.4 | [M+H]+ | 279.1611 | Tolycaine |
| 238600 | 65440 | 15.28 | [M+H]+ | 255.2324 | PALMITOLEATE |
| 149200 | 42740 | 15.28 | [M+H]+ | 237.2215 | Ambroxane |
| 476200 | 114800 | 14.9 | [M+H]+ | 279.2328 | Alpha-Linolenic acid |
| 55140 | 18120 | 14.79 | [M+H]+ | 303.2331 | 17a-Methyltestosterone |
| 55140 | 18120 | 14.79 | [M+H]+ | 303.2331 | 17a-Methyltestosterone |
| 59100 | 19390 | 14.79 | [M+H]+ | 303.2331 | EICOSAPENTAENOATE |
| 59100 | 19390 | 14.79 | [M+H]+ | 303.2331 | EICOSAPENTAENOATE |
| 55140 | 18120 | 14.79 | [M+H]+ | 303.2331 | Kaurenoic acid |
| 55140 | 18120 | 14.79 | [M+H]+ | 303.2331 | Kaurenoic acid |
| 18150 | 2030 | 14.47 | [M+H]+ | 89.0597 | ACETOIN |
| 18150 | 2030 | 14.47 | [M+H]+ | 89.0597 | BUTANOATE |
| 18150 | 2030 | 14.47 | [M+H]+ | 89.0597 | ISOBUTYRATE |
| 18200 | 3612 | 14.13 | [M+H]+ | 251.1996 | Sclareolide |
| 27510 | 2671 | 13.88 | [M+H]+ | 123.0799 | 2,6-Xylenol |
| 34130 | 3280 | 13.86 | [M+H]+ | 100.0754 | 1-Methyl-2-pyrrolidone |
| 12470 | 1688 | 13.8 | [M+H]+ | 279.1305 | Oxadixyl |
| 460600 | 77090 | 13.8 | [M+H]+ | 279.1597 | Tolycaine |
| 801500 | 139500 | 13.79 | [M+H]+ | 279.1592 | Dibutyl phthalate |
| 857000 | 148600 | 13.79 | [M+H]+ | 279.1592 | DIISOBUTYL PHTHALATE |
| 19040 | 3132 | 13.79 | [M+H]+ | 279.1305 | Pantetheine |
| 51840 | 15720 | 13.78 | [M+H]+ | 201.1849 | Ethyl decanoate |
| 29200 | 7510 | 13.69 | [M+H]+ | 279.2321 | GAMMA-LINOLENATE |
| 1435000 | 435400 | 13.36 | [M+H]+ | 303.2319 | EICOSAPENTAENOATE |
| 1332000 | 404700 | 13.36 | [M+H]+ | 303.2319 | Kaurenoic acid |
| 14300 | 4012 | 13.36 | [M+H]+ | 237.1847 | Curdione |
| 14300 | 4012 | 13.36 | [M+H]+ | 237.1847 | Curcumol |
| 527400 | 97390 | 13.04 | [M+H]+ | 428.3734 | Stearoylcarnitine (C18) |
| 1263 | 274 | 13.03 | [M+H]+ | 760.5034 | Spinetoram B |
| 106600 | 25060 | 12.97 | [M+H]+ | 279.2318 | GAMMA-LINOLENATE |
| 34150 | 8931 | 12.86 | [M+H]+ | 327.0778 | Triphenylphosphate |
| 125600 | 36200 | 12.75 | [M+H]+ | 301.2164 | 13-cis-Retinoic acid |
| 115000 | 33780 | 12.75 | [M+H]+ | 301.2164 | Vitamin A acid |
| 26890 | 7322 | 12.7 | [M+H]+ | 255.2319 | PALMITOLEATE |
| 18400 | 2058 | 12.51 | [M+H]+ | 279.1614 | Tolycaine |
| 1281000 | 246500 | 12.38 | [M+H]+ | 426.3598 | OleylCarnitine(C18:1) |
| 22950 | 5836 | 12.37 | [M+H]+ | 281.249 | LINOLEATE |
| 25300 | 7606 | 12.34 | [M+H]+ | 173.154 | DECANOATE |
| 20280 | 6218 | 12.29 | [M+H]+ | 149.0961 | Anise oil |
| 8334 | 1978 | 12.25 | [M+H]+ | 393.2992 | Chenodeoxycholic acid |
| 1781000 | 352600 | 12.18 | [M+H]+ | 400.3419 | PALMITOYLCARNITINE |
| 12740 | 2723 | 12.15 | [M+H]+ | 149.0597 | TRANS-CINNAMATE |
| 7285 | 1417 | 12.1 | [M+H]+ | 284.2946 | Octadecanamide |
| 52830 | 17120 | 11.96 | [M+H]+ | 362.0397 | Haloxyfop-P |
| 47910 | 12710 | 11.92 | [M+H]+ | 195.1014 | Butylparaben |
| 20300 | 5109 | 11.92 | [M+H]+ | 167.0701 | Ethylsalicylate |
| 18520 | 4990 | 11.92 | [M+H]+ | 167.0701 | Ethyl 4-hydroxybenzoate |
| 793300 | 176300 | 11.83 | [M+H]+ | 424.3417 | Ciscis9,12-Octadecadienoylcarnitine(C18:2) |
| 29440 | 8134 | 11.82 | [M+H]+ | 585.2709 | BILIRUBIN |
| 118300 | 23810 | 11.81 | [M+H]+ | 448.3419 | Arachidorylcarnitine(C20:4) |
| 6942 | 1958 | 11.66 | [M+H]+ | 153.1272 | cis-Citral |
| 35830 | 10320 | 11.63 | [M+H]+ | 279.232 | GAMMA-LINOLENATE |
| 2978 | 296 | 11.59 | [M+H]+ | 279.1302 | Oxadixyl |
| 622100 | 170500 | 11.38 | [M+H]+ | 393.2859 | DEOXYCHOLATE |
| 622100 | 170500 | 11.38 | [M+H]+ | 393.2859 | Hyodeoxycholic Acid |
| 622100 | 170500 | 11.38 | [M+H]+ | 393.2859 | Hyodeoxycholic Acid |
| 213400 | 56330 | 11.38 | [M+H]+ | 393.2859 | Hyodeoxycholic Acid |
| 622100 | 170500 | 11.38 | [M+H]+ | 393.2859 | URSODEOXYCHOLATE |
| 352400 | 101800 | 11.38 | [M+H]+ | 376.2588 | Tuberostemonine |
| 33770 | 7402 | 11.33 | [M+H]+ | 302.3055 | SPHINGANINE |
| 447000 | 99410 | 11.29 | [M+H]+ | 372.3107 | Myristoylcar-nitine (C14) |
| 9977 | 1692 | 11.25 | [M+H]+ | 237.1848 | Curcumol |
| 56840 | 13430 | 11.16 | [M+H]+ | 583.2543 | BILIVERDIN |
| 212500 | 63040 | 11.14 | [M+H]+ | 391.2845 | Nutriacholic acid |
| 18050 | 5575 | 11.08 | [M+H]+ | 328.0792 | Fluazifop free acid |
| 34230 | 9465 | 10.98 | [M+H]+ | 450.3212 | GLYCOCHENODEOXYCHOLATE |
| 13290 | 2813 | 10.96 | [M+H]+ | 177.0545 | Hymecromone |
| 13290 | 2813 | 10.96 | [M+H]+ | 177.0545 | Hymecromone |
| 67170 | 9969 | 10.93 | [M+H]+ | 500.3035 | Taurodeoxycholic acid |
| 876 | 185 | 10.89 | [M+H]+ | 295.2593 | Methyllinoleate |
| 35810 | 10320 | 10.74 | [M+H]+ | 253.1796 | 15-Hydroxyculmorone |
| 71270 | 19910 | 10.66 | [M+H]+ | 583.2544 | BILIVERDIN |
| 172100 | 46310 | 10.64 | [M+H]+ | 391.284 | Nutriacholic acid |
| 36790 | 9360 | 10.48 | [M+H]+ | 387.2517 | Bufalin |
| 21450 | 6249 | 10.43 | [M+H]+ | 245.1746 | 1,11-Undecanedicarboxylic acid |
| 110000 | 30730 | 10.43 | [M+H]+ | 227.1641 | Methyl dihydrojasmonate |
| 152600 | 38820 | 10.39 | [M+H]+ | 344.2793 | LAUROYLCARNITINE |
| 15650 | 4670 | 10.34 | [M+H]+ | 233.1534 | Alantolactone |
| 15650 | 4670 | 10.34 | [M+H]+ | 233.1534 | Isoalantolactone |
| 15650 | 4670 | 10.34 | [M+H]+ | 233.1534 | Costunolide |
| 7900 | 1161 | 10.26 | [M+H]+ | 149.0952 | Anise oil |
| 7490 | 1822 | 10.17 | [M+H]+ | 171.1379 | Linalool oxide |
| 189600 | 55350 | 10.07 | [M+H]+ | 391.2839 | Nutriacholic acid |
| 55190 | 13190 | 10 | [M+H]+ | 279.159 | Dibutyl phthalate |
| 59200 | 14450 | 10 | [M+H]+ | 279.159 | DIISOBUTYL PHTHALATE |
| 33000 | 9488 | 9.99 | [M+H]+ | 165.0911 | Isoeugenol |
| 8164 | 596 | 9.98 | [M+H]+ | 253.1785 | 15-Hydroxyculmorone |
| 54000 | 15170 | 9.87 | [M+H]+ | 251.1641 | Nardosinone |
| 7026 | 2030 | 9.8 | [M+H]+ | 231.1587 | Dodecanedioic acid |
| 7026 | 2030 | 9.8 | [M+H]+ | 231.1587 | Dodecanedioic acid |
| 7026 | 2030 | 9.8 | [M+H]+ | 231.1587 | Dodecanedioic acid |
| 6525 | 1923 | 9.8 | [M+H]+ | 231.1587 | Dodecanedioic acid |
| 28000 | 7912 | 9.75 | [M+H]+ | 450.3211 | GLYCOCHENODEOXYCHOLATE |
| 17430 | 3535 | 9.54 | [M+H]+ | 249.1488 | Eremofortin B |
| 8245 | 2047 | 9.51 | [M+H]+ | 151.0752 | 4-Ethylbenzoic Acid |
| 8245 | 2047 | 9.51 | [M+H]+ | 151.0752 | Hydrocinnamic acid |
| 8245 | 2047 | 9.51 | [M+H]+ | 151.0752 | Hydrocinnamic acid |
| 7640 | 1971 | 9.51 | [M+H]+ | 151.0752 | Hydrocinnamic acid |
| 56320 | 11420 | 9.45 | [M+H]+ | 516.2986 | Taurocholic acid |
| 41910 | 11800 | 9.42 | [M+H]+ | 316.248 | Decanoylcarnitine (C10) |
| 7847 | 1357 | 9.4 | [M+H]+ | 502.3052 | Fexofenadine |
| 120700 | 24970 | 9.4 | [M+H]+ | 500.3036 | Taurodeoxycholic acid |
| 36910 | 10630 | 9.36 | [M+H]+ | 777.6942 | THYROXINE |
| 93720 | 21290 | 9.26 | [M+H]+ | 585.2702 | BILIRUBIN |
| 17350 | 5252 | 9.12 | [M+H]+ | 345.242 | 4-Beta-hydroxystanozolol |
| 34640 | 9900 | 8.94 | [M+H]+ | 267.159 | Aspterric acid |
| 34640 | 9900 | 8.94 | [M+H]+ | 267.159 | Sambucinol |
| 4112 | 1294 | 8.9 | [M+H]+ | 149.0593 | cinnamic acid |
| 31690 | 8581 | 8.83 | [M+H]+ | 363.2163 | CORTISOL |
| 508100 | 153600 | 8.74 | [M+H]+ | 190.0863 | 3-Indolepropionic acid |
| 508100 | 153600 | 8.74 | [M+H]+ | 190.0863 | INDOLE-3-METHYL ACETATE |
| 187100 | 56480 | 8.74 | [M+H]+ | 130.0652 | Isoquinoline |
| 24880 | 4197 | 8.54 | [M+H]+ | 516.2989 | Taurocholic acid |
| 35080 | 11070 | 8.48 | [M+H]+ | 188.0702 | Indoleacrylic acid |
| 6190 | 1681 | 8.42 | [M+H]+ | 143.1066 | Cis-3-Hexenylacetate |
| 30150 | 9221 | 8.33 | [M+H]+ | 288.2167 | Octanoylcarnitine (C8) |
| 34770 | 5618 | 8.11 | [M+H]+ | 331.2108 | 11a-Hydroxyprogesterone |
| 2859 | 648 | 8.02 | [M+H]+ | 279.0389 | Acecarbromal |
| 11530 | 2135 | 7.95 | [M+H]+ | 165.091 | 3-Phenylbutyric acid |
| 11530 | 2135 | 7.95 | [M+H]+ | 165.091 | Cumic acid |
| 115500 | 25000 | 7.77 | [M+H]+ | 163.1119 | 5-HYDROXYLYSINE |
| 36550 | 11150 | 7.44 | [M+H]+ | 247.1079 | N-Acetyl-L-tryptophan |
| 20850 | 5280 | 7.34 | [M+H]+ | 188.0707 | Indoleacrylic acid |
| 56850 | 17580 | 7.02 | [M+H]+ | 260.185 | Hexanoylcarnitine (C6) |
| 125700 | 34290 | 6.96 | [M+H]+ | 569.3114 | Helvolic acid |
| 9269000 | 2651000 | 6.96 | [M+H]+ | 564.3589 | PEG-12mer Ammonium adduct |
| 19670 | 5985 | 6.93 | [M+H]+ | 174.0548 | 2-QUINOLINECARBOXYLATE |
| 16030 | 4785 | 6.93 | [M+H]+ | 146.0595 | 4-Hydroxyquinoline |
| 19670 | 5985 | 6.93 | [M+H]+ | 174.0548 | 4-QUINOLINECARBOXYLATE |
| 16030 | 4785 | 6.93 | [M+H]+ | 146.0595 | 8-Hydroxyquinoline |
| 11030 | 3344 | 6.9 | [M+H]+ | 174.1121 | N-ACETYLLEUCINE |
| 10520 | 3247 | 6.9 | [M+H]+ | 174.1121 | N-Acetyl-L-leucine |
| 10520 | 3247 | 6.9 | [M+H]+ | 174.1121 | N-Acetyl-L-leucine |
| 10300000 | 2918000 | 6.83 | [M+H]+ | 520.3316 | PEG-11mer Ammonium adduct |
| 4984 | 642 | 6.8 | [M+H]+ | 107.0825 | 1,4-Xylene |
| 4984 | 642 | 6.8 | [M+H]+ | 107.0825 | m-Xylene |
| 4984 | 642 | 6.8 | [M+H]+ | 107.0825 | o-Xylene |
| 147800 | 44830 | 6.78 | [M+H]+ | 194.0808 | Phenylacetylglycine |
| 1100 | 217 | 6.78 | [M+H]+ | 89.1069 | PUTRESCINE |
| 11150 | 3529 | 6.75 | [M+H]+ | 174.1116 | N-ACETYLLEUCINE |
| 9403000 | 2627000 | 6.68 | [M+H]+ | 476.3061 | PEG-10mer Ammonium adduct |
| 4998 | 983 | 6.59 | [M+H]+ | 279.1398 | Pantetheine |
| 7515000 | 2099000 | 6.52 | [M+H]+ | 432.2794 | PEG-9mer Ammonium adduct |
| 10990 | 2898 | 6.42 | [M+H]+ | 105.0332 | 3-(Methylthio)propionaldehyde |
| 37580 | 8649 | 6.42 | [M+H]+ | 180.0654 | HIPPURATE |
| 37580 | 8649 | 6.42 | [M+H]+ | 180.0654 | Hippuric acid |
| 46920 | 14250 | 6.41 | [M+H]+ | 377.1449 | Riboflavin |
| 41090 | 12250 | 6.41 | [M+H]+ | 377.1449 | Vitamin B2 |
| 19580 | 4463 | 6.39 | [M+H]+ | 134.0602 | Indoxyl |
| 5842000 | 1695000 | 6.33 | [M+H]+ | 388.254 | PEG-8mer Ammonium adduct |
| 26240 | 2490 | 6.24 | [M+H]+ | 279.1592 | Tolycaine |
| 17060 | 4923 | 6.23 | [M+H]+ | 192.0655 | 5-HYDROXYINDOLEACETATE |
| 17060 | 4923 | 6.23 | [M+H]+ | 192.0655 | 5-Hydroxyindoleacetic acid |
| 4376 | 1009 | 6.19 | [M+H]+ | 379.1346 | Mefloquine |
| 10060 | 2275 | 6.14 | [M+H]+ | 174.054 | 2-QUINOLINECARBOXYLATE |
| 10060 | 2275 | 6.14 | [M+H]+ | 174.054 | Quinaldic acid |
| 3460000 | 1006000 | 6.13 | [M+H]+ | 344.2276 | PEG-7mer Ammonium adduct |
| 159700 | 46710 | 6.06 | [M+H]+ | 246.1697 | Isovalerylcarnitine |
| 34950 | 3655 | 6.04 | [M+H]+ | 100.0753 | 1-Methyl-2-pyrrolidone |
| 57420 | 5175 | 6 | [M+H]+ | 279.1589 | Dibutyl phthalate |
| 8960 | 1056 | 6 | [M+H]+ | 88.0748 | N-ETHYLACETAMIDE |
| 60570 | 5461 | 5.99 | [M+H]+ | 279.1589 | DIISOBUTYL PHTHALATE |
| 4456 | 1038 | 5.87 | [M+H]+ | 127.0392 | 2,3-Dimethylmaleic anhydride |
| 4858 | 1019 | 5.87 | [M+H]+ | 127.0392 | Maltol |
| 8505 | 1093 | 5.79 | [M+H]+ | 137.0588 | 4-Methylbenzoic acid |
| 8505 | 1093 | 5.79 | [M+H]+ | 137.0588 | m-Toluic acid |
| 8505 | 1093 | 5.79 | [M+H]+ | 137.0588 | PHENYL ACETATE |
| 5620 | 936 | 5.78 | [M+H]+ | 137.0587 | 4-Methoxybenzaldehyde |
| 5620 | 936 | 5.78 | [M+H]+ | 137.0587 | o-Toluic acid |
| 35530 | 9506 | 5.71 | [M+H]+ | 200.0474 | 2-Chloro-L-phenylalanine |
| 1666 | 447 | 5.7 | [M+H]+ | 280.1821 | Moxisylyte |
| 203600 | 47020 | 5.68 | [M+H]+ | 144.0802 | 1-NAPHTHYLAMINE |
| 88570 | 20480 | 5.68 | [M+H]+ | 132.0807 | 3-Methylindole |
| 7516000 | 1697000 | 5.68 | [M+H]+ | 188.0706 | Indoleacrylic acid |
| 7298000 | 1682000 | 5.68 | [M+H]+ | 205.0972 | L-Tryptophan |
| 296800 | 67710 | 5.67 | [M+H]+ | 146.0599 | 4-Hydroxyquinoline |
| 1231 | 319 | 5.65 | [M+H]+ | 275.0674 | Neburon |
| 34740 | 11370 | 5.63 | [M+H]+ | 298.0968 | 5-Methylthioadenosine |
| 36470 | 11940 | 5.63 | [M+H]+ | 298.0968 | METHYLTHIOADENOSINE |
| 5585 | 1608 | 5.6 | [M+H]+ | 150.0578 | L-Methionine |
| 5585 | 1608 | 5.6 | [M+H]+ | 150.0578 | METHIONINE |
| 12840 | 1033 | 5.5 | [M+H]+ | 205.1945 | Patchouli alcohol (loss H20) |
| 81370 | 6372 | 5.49 | [M+H]+ | 279.1588 | Dibutyl phthalate |
| 30480 | 1506 | 5.44 | [M+H]+ | 101.0957 | 2-Hexanone |
| 30480 | 1506 | 5.44 | [M+H]+ | 101.0957 | 2-Methylpentan-3-one |
| 347500 | 53450 | 5.44 | [M+H]+ | 232.1541 | Butyrylcarnitine(C4) |
| 347500 | 53450 | 5.44 | [M+H]+ | 232.1541 | Isobutyrylcarnitine (isoC4) |
| 30480 | 1506 | 5.44 | [M+H]+ | 101.0957 | Methyl isobutyl ketone |
| 128400 | 8308 | 5.43 | [M+H]+ | 279.1588 | DIISOBUTYL PHTHALATE |
| 6043 | 472 | 5.42 | [M+H]+ | 129.1261 | Octanal_ |
| 88970 | 5340 | 5.38 | [M+H]+ | 100.0753 | 1-Methyl-2-pyrrolidone |
| 46420 | 6392 | 5.37 | [M+H]+ | 279.1337 | Oxadixyl |
| 49760 | 6544 | 5.37 | [M+H]+ | 279.1349 | Pantetheine |
| 781200 | 142200 | 5.34 | [M+H]+ | 220.1181 | PANTOTHENATE |
| 781200 | 142200 | 5.34 | [M+H]+ | 220.1181 | Pantothenic acid |
| 29820 | 4393 | 5.33 | [M+H]+ | 101.0589 | Tiglic acid |
| 25710 | 4090 | 5.32 | [M+H]+ | 101.0589 | 2,3-Pentanedione |
| 9068 | 1974 | 5.23 | [M+H]+ | 180.0871 | GALACTOSAMINE |
| 9068 | 1974 | 5.23 | [M+H]+ | 180.0871 | GLUCOSAMINE |
| 67410 | 14630 | 5.22 | [M+H]+ | 127.05 | Thymine |
| 65620 | 14440 | 5.22 | [M+H]+ | 127.05 | Thymine |
| 65620 | 14440 | 5.22 | [M+H]+ | 127.05 | Thymine |
| 7585 | 1723 | 5.17 | [M+H]+ | 282.1181 | 1-Methyladenosine |
| 125000 | 17500 | 5.16 | [M+H]+ | 122.0961 | 2,4-Dimethylaniline |
| 125000 | 17500 | 5.16 | [M+H]+ | 122.0961 | 2,6-Dimethylaniline |
| 125000 | 17500 | 5.16 | [M+H]+ | 122.0961 | N,N-Dimethylaniline |
| 132600 | 18510 | 5.16 | [M+H]+ | 122.0961 | PHENETHYLAMINE |
| 5876 | 1362 | 5.15 | [M+H]+ | 122.0605 | Benzamide |
| 4582000 | 1085000 | 5.15 | [M+H]+ | 166.0863 | L-Phenylalanine |
| 4582000 | 1085000 | 5.15 | [M+H]+ | 166.0863 | PHENYLALANINE |
| 4516000 | 1072000 | 5.15 | [M+H]+ | 166.0863 | Phenprobamate |
| 4516000 | 1072000 | 5.15 | [M+H]+ | 166.0863 | Phenylalanine |
| 12200 | 3575 | 5.11 | [M+H]+ | 146.06 | 4-Hydroxyquinoline |
| 124200 | 28930 | 5.11 | [M+H]+ | 192.0654 | 5-HYDROXYINDOLEACETATE |
| 270000 | 68310 | 5.11 | [M+H]+ | 209.0918 | KYNURENINE |
| 1166 | 239 | 5.1 | [M+H]+ | 137.0758 | 6-Methylnicotinamide |
| 1975 | 491 | 5.04 | [M+H]+ | 166.1167 | Hordenine |
| 148700 | 19090 | 5.02 | [M+H]+ | 194.1175 | BDB |
| 475100 | 90340 | 5.01 | [M+H]+ | 160.0754 | INDOLEACETALDEHYDE |
| 8526 | 1125 | 5.01 | [M+H]+ | 182.0809 | L-Tyrosine |
| 8526 | 1125 | 5.01 | [M+H]+ | 182.0809 | o-Tyrosine |
| 101400 | 22380 | 5.01 | [M+H]+ | 177.1022 | Serotonin |
| 8526 | 1125 | 5.01 | [M+H]+ | 182.0809 | TYROSINE |
| 39600 | 11780 | 5 | [M+H]+ | 285.0827 | XANTHOSINE |
| 23940 | 7243 | 4.96 | [M+H]+ | 175.1184 | ARGININE |
| 23940 | 7243 | 4.96 | [M+H]+ | 175.1184 | L-Arginine |
| 24530 | 7114 | 4.96 | [M+H]+ | 175.1184 | L(+)-Arginine |
| 5311 | 666 | 4.88 | [M+H]+ | 169.039 | URATE |
| 16950 | 3024 | 4.86 | [M+H]+ | 204.1228 | L-Acetylcarnitine |
| 75410 | 10160 | 4.48 | [M+H]+ | 218.1385 | Propionylcarnitine (C3) |
| 54960 | 4854 | 3.28 | [M+H]+ | 154.0496 | 3-AMINO-4-HYDROXYBENZOATE |
| 20330 | 2038 | 3.27 | [M+H]+ | 113.0342 | URACIL |
| 16960 | 1895 | 3.27 | [M+H]+ | 245.0769 | URIDINE |
| 542100 | 55980 | 3.05 | [M+H]+ | 165.0545 | 4-COUMARATE |
| 54260 | 6475 | 3.05 | [M+H]+ | 123.0437 | 4-HYDROXYBENZALDEHYDE |
| 1536000 | 151300 | 3.05 | [M+H]+ | 182.0811 | L-Tyrosine |
| 1536000 | 151300 | 3.05 | [M+H]+ | 182.0811 | o-Tyrosine |
| 1536000 | 151300 | 3.05 | [M+H]+ | 182.0811 | TYROSINE |
| 61180 | 6400 | 3.04 | [M+H]+ | 147.044 | Coumarin |
| 1079 | 219 | 2.98 | [M+H]+ | 129.0654 | 5,6-Dihydro-5-Methyluracil |
| 1990 | 468 | 2.98 | [M+H]+ | 132.0665 | TRANS-4-HYDROXY-L-PROLINE |
| 2091 | 288 | 2.96 | [M+H]+ | 146.08 | 4-Acetamidobutyric Acid |
| 1894000 | 211500 | 2.92 | [M+H]+ | 132.1017 | Alloisoleucine |
| 1894000 | 211500 | 2.92 | [M+H]+ | 132.1017 | ISOLEUCINE |
| 1894000 | 211500 | 2.92 | [M+H]+ | 132.1017 | L-Alloisoleucine |
| 1894000 | 211500 | 2.92 | [M+H]+ | 132.1017 | LEUCINE |
| 1894000 | 211500 | 2.92 | [M+H]+ | 132.1017 | L-Isoleucine |
| 1894000 | 211500 | 2.92 | [M+H]+ | 132.1017 | L-Leucine |
| 1894000 | 211500 | 2.92 | [M+H]+ | 132.1017 | L-Norleucine |
| 1894000 | 211500 | 2.92 | [M+H]+ | 132.1017 | NORLEUCINE |
| 1876000 | 209300 | 2.92 | [M+H]+ | 132.1017 | Leucine |
| 1876000 | 209300 | 2.92 | [M+H]+ | 132.1017 | Isoleucine |
| 10810 | 1242 | 2.9 | [M+H]+ | 282.1199 | 1-Methyladenosine |
| 42340 | 4840 | 2.88 | [M+H]+ | 126.0658 | 5-METHYLCYTOSINE |
| 43220 | 3911 | 2.47 | [M+H]+ | 100.0754 | 1-Methyl-2-pyrrolidone |
| 1430 | 150 | 2.44 | [M+H]+ | 101.0589 | 2,3-Pentanedione |
| 178100 | 9668 | 2.42 | [M+H]+ | 218.1386 | Propionylcarnitine (C3) |
| 1367 | 160 | 2.37 | [M+H]+ | 147.0761 | Ureidoisobutyric Acid |
| 1172 | 129 | 2.35 | [M+H]+ | 130.0848 | 1-Aminocyclopentanecarboxylic acid |
| 1172 | 129 | 2.35 | [M+H]+ | 130.0848 | Pipecolinic acid |
| 88840 | 12290 | 2.29 | [M+H]+ | 130.0495 | OXOPROLINE |
| 88840 | 12290 | 2.29 | [M+H]+ | 130.0495 | PYROGLUTAMATE |
| 88840 | 12290 | 2.29 | [M+H]+ | 130.0495 | Pyroglutamic acid |
| 88840 | 12290 | 2.29 | [M+H]+ | 130.0495 | Pyrrolidonecarboxylic acid |
| 677100 | 90060 | 2.29 | [M+H]+ | 169.0355 | URATE |
| 247400 | 28590 | 2.15 | [M+H]+ | 112.0504 | CYTOSINE |
| 133400 | 15520 | 2.15 | [M+H]+ | 228.098 | DEOXYCYTIDINE |
| 6940 | 1144 | 2.08 | [M+H]+ | 193.0333 | Citric acid |
| 121600 | 11680 | 2.07 | [M+H]+ | 123.0551 | Isonicotinamide |
| 121600 | 11680 | 2.07 | [M+H]+ | 123.0551 | Niacinamide |
| 121600 | 11680 | 2.07 | [M+H]+ | 123.0551 | NICOTINAMIDE |
| 123500 | 11610 | 2.07 | [M+H]+ | 123.0551 | NICOTINAMIDE |
| 123500 | 11610 | 2.07 | [M+H]+ | 123.0551 | Nicotinamide |
| 4331 | 365 | 2.01 | [M+H]+ | 180.0319 | S-CARBOXYMETHYLCYSTEINE |
| 3424 | 302 | 1.99 | [M+H]+ | 107.0844 | 1,4-Xylene |
| 3424 | 302 | 1.99 | [M+H]+ | 107.0844 | m-Xylene |
| 3424 | 302 | 1.99 | [M+H]+ | 107.0844 | o-Xylene |
| 11700 | 1816 | 1.98 | [M+H]+ | 139.0499 | UROCANATE |
| 11700 | 1816 | 1.98 | [M+H]+ | 139.0499 | Urocanic acid |
| 40900 | 3046 | 1.95 | [M+H]+ | 194.1175 | BDB |
| 100400 | 9371 | 1.87 | [M+H]+ | 87.0437 | DIACETYL |
| 99020 | 9301 | 1.87 | [M+H]+ | 87.0437 | Mesaconic acid_-CO2 |
| 1163000 | 146800 | 1.84 | [M+H]+ | 204.1232 | L-Acetylcarnitine |
| 3751 | 427 | 1.8 | [M+H]+ | 129.0657 | 5,6-Dihydro-5-Methyluracil |
| 3866000 | 323900 | 1.72 | [M+H]+ | 132.1017 | Alloisoleucine |
| 3866000 | 323900 | 1.72 | [M+H]+ | 132.1017 | ISOLEUCINE |
| 3866000 | 323900 | 1.72 | [M+H]+ | 132.1017 | LEUCINE |
| 3866000 | 323900 | 1.72 | [M+H]+ | 132.1017 | L-Leucine |
| 3866000 | 323900 | 1.72 | [M+H]+ | 132.1017 | L-Norleucine |
| 3866000 | 323900 | 1.72 | [M+H]+ | 132.1017 | NORLEUCINE |
| 3829000 | 321800 | 1.72 | [M+H]+ | 132.1017 | Leucine |
| 3829000 | 321800 | 1.72 | [M+H]+ | 132.1017 | Isoleucine |
| 7145 | 1006 | 1.57 | [M+H]+ | 194.1174 | BDB |
| 24680 | 2329 | 1.55 | [M+H]+ | 130.086 | 1-Aminocyclopentanecarboxylic acid |
| 24680 | 2329 | 1.55 | [M+H]+ | 130.086 | Pipecolinic acid |
| 4489 | 543 | 1.53 | [M+H]+ | 144.1016 | Stachydrine hydrochloride |
| 2542 | 214 | 1.51 | [M+H]+ | 157.1219 | gamma-Nonanolactone |
| 2266000 | 392700 | 1.4 | [M+H]+ | 132.1016 | Leucine |
| 2266000 | 392700 | 1.4 | [M+H]+ | 132.1016 | Isoleucine |
| 2266000 | 392700 | 1.4 | [M+H]+ | 132.1016 | Isoleucine |
| 1087000 | 305400 | 1.32 | [M+H]+ | 165.0545 | 4-COUMARATE |
| 23790 | 4638 | 1.32 | [M+H]+ | 91.0389 | GLYCERALDEHYDE |
| 23790 | 4638 | 1.32 | [M+H]+ | 91.0389 | LACTATE |
| 3325000 | 925800 | 1.32 | [M+H]+ | 182.0812 | L-Tyrosine |
| 3325000 | 925800 | 1.32 | [M+H]+ | 182.0812 | o-Tyrosine |
| 3325000 | 925800 | 1.32 | [M+H]+ | 182.0812 | TYROSINE |
| 205100 | 35090 | 1.3 | [M+H]+ | 123.0434 | 4-HYDROXYBENZALDEHYDE |
| 215600 | 73350 | 1.27 | [M+H]+ | 130.0855 | 1-Aminocyclopentanecarboxylic acid |
| 390500 | 121700 | 1.27 | [M+H]+ | 244.0927 | CYTIDINE |
| 549500 | 170900 | 1.27 | [M+H]+ | 112.0504 | CYTOSINE |
| 339600 | 105300 | 1.27 | [M+H]+ | 228.0979 | DEOXYCYTIDINE |
| 6435 | 1926 | 1.27 | [M+H]+ | 91.0389 | DL-Glyceraldehyde |
| 3087000 | 966800 | 1.27 | [M+H]+ | 204.123 | L-Acetylcarnitine |
| 754600 | 238800 | 1.27 | [M+H]+ | 150.0581 | L-Methionine |
| 754600 | 238800 | 1.27 | [M+H]+ | 150.0581 | METHIONINE |
| 224200 | 75220 | 1.27 | [M+H]+ | 130.0855 | PIPECOLATE |
| 224200 | 75220 | 1.27 | [M+H]+ | 130.0855 | Pipecolic acid |
| 347700 | 110400 | 1.27 | [M+H]+ | 169.0355 | URATE |
| 215600 | 73350 | 1.27 | [M+H]+ | 130.0855 | Pipecolinic acid |
| 368500 | 116300 | 1.27 | [M+H]+ | 244.0927 | Cytidine |
| 773400 | 246200 | 1.26 | [M+H]+ | 118.0859 | L-Valine |
| 773400 | 246200 | 1.26 | [M+H]+ | 118.0859 | NORVALINE |
| 773400 | 246200 | 1.26 | [M+H]+ | 118.0859 | VALINE |
| 764300 | 244700 | 1.26 | [M+H]+ | 118.0859 | L-Valine |
| 26570 | 5605 | 1.25 | [M+H]+ | 146.0924 | 4-GUANIDINOBUTANOATE |
| 1022000 | 377500 | 1.23 | [M+H]+ | 144.1019 | Stachydrine hydrochloride |
| 116100 | 23500 | 1.2 | [M+H]+ | 308.0913 | Glutathione |
| 116100 | 23500 | 1.2 | [M+H]+ | 308.0913 | Glutathione |
| 24830 | 6198 | 1.16 | [M+H]+ | 162.1124 | L-Carnitine |
| 328100 | 94920 | 1.14 | [M+H]+ | 146.1172 | DEOXYCARNITINE |
| 60120 | 18370 | 1.14 | [M+H]+ | 190.1185 | Homocitrulline |
| 249900 | 63310 | 1.14 | [M+H]+ | 203.1502 | N,N-DIMETHYLARGININE |
| 249900 | 63310 | 1.14 | [M+H]+ | 203.1502 | N,N-DIMETHYL-ARGININE |
| 182800 | 53900 | 1.12 | [M+H]+ | 138.0545 | 3-Pyridylacetic acid |
| 182800 | 53900 | 1.12 | [M+H]+ | 138.0545 | 4-AMINOBENZOATE |
| 178100 | 53310 | 1.12 | [M+H]+ | 138.0545 | 4-Aminobenzoic acid |
| 182800 | 53900 | 1.12 | [M+H]+ | 138.0545 | ANTHRANILATE |
| 182800 | 53900 | 1.12 | [M+H]+ | 138.0545 | p-Aminobenzoic acid |
| 182800 | 53900 | 1.12 | [M+H]+ | 138.0545 | TRIGONELLINE |
| 178100 | 53310 | 1.12 | [M+H]+ | 138.0545 | Trigonelline |
| 41970 | 10500 | 1.08 | [M+H]+ | 162.0761 | AMINOADIPATE |
| 41970 | 10500 | 1.08 | [M+H]+ | 162.0761 | Aminoadipic acid |
| 41970 | 10500 | 1.08 | [M+H]+ | 162.0761 | N-METHYLGLUTAMATE |
| 936900 | 280300 | 1.07 | [M+H]+ | 116.0699 | L-Proline |
| 918000 | 278000 | 1.07 | [M+H]+ | 116.0699 | Proline |
| 7721000 | 2366000 | 1.05 | [M+H]+ | 132.0765 | CREATINE |
| 20000 | 6780 | 1.03 | [M+H]+ | 345.0783 | THIAMINE MONOPHOSPHATE |
| 5198 | 822 | 1.02 | [M+H]+ | 175.0518 | SHIKIMATE |
| 2219000 | 639500 | 1.01 | [M+H]+ | 118.0861 | BETAINE |
| 2219000 | 639500 | 1.01 | [M+H]+ | 118.0861 | N-Methyl-a-aminoisobutyric acid |
| 2196000 | 635800 | 1.01 | [M+H]+ | 118.0861 | Betaine |
| 869600 | 299300 | 1 | [M+H]+ | 162.1124 | L-CARNITINE |
| 869600 | 299300 | 1 | [M+H]+ | 162.1124 | L-CARNITINE |
| 853400 | 294200 | 1 | [M+H]+ | 162.1124 | L-Carnitine |
| 40000 | 6475 | 0.99 | [M+H]+ | 104.0705 | 2-Aminoisobutyric Acid |
| 40000 | 6475 | 0.99 | [M+H]+ | 104.0705 | N-Methyl-L-alanine |
| 437900 | 119500 | 0.98 | [M+H]+ | 176.1029 | Citrulline |
| 316300 | 88570 | 0.98 | [M+H]+ | 258.1099 | Glycerophosphocholine |
| 406000 | 116900 | 0.98 | [M+H]+ | 176.1029 | Citrulline |
| 203800 | 56060 | 0.95 | [M+H]+ | 148.0601 | Glutamic acid |
| 240900 | 64730 | 0.94 | [M+H]+ | 148.0601 | GLUTAMATE |
| 240900 | 64730 | 0.94 | [M+H]+ | 148.0601 | L-Glutamic acid |
| 160700 | 47910 | 0.93 | [M+H]+ | 120.0651 | ALLOTHREONINE |
| 637500 | 220000 | 0.93 | [M+H]+ | 147.0762 | D-Glutamine |
| 637500 | 220000 | 0.93 | [M+H]+ | 147.0762 | GLUTAMINE |
| 160700 | 47910 | 0.93 | [M+H]+ | 120.0651 | HOMOSERINE |
| 150000 | 47610 | 0.93 | [M+H]+ | 120.0651 | L-allo-Threonine |
| 637500 | 220000 | 0.93 | [M+H]+ | 147.0762 | L-Glutamine |
| 160700 | 47910 | 0.93 | [M+H]+ | 120.0651 | L-Homoserine |
| 160700 | 47910 | 0.93 | [M+H]+ | 120.0651 | L-Threonine |
| 598800 | 200500 | 0.93 | [M+H]+ | 130.0497 | OXOPROLINE |
| 598800 | 200500 | 0.93 | [M+H]+ | 130.0497 | PYROGLUTAMATE |
| 598800 | 200500 | 0.93 | [M+H]+ | 130.0497 | Pyroglutamic acid |
| 598800 | 200500 | 0.93 | [M+H]+ | 130.0497 | Pyrrolidonecarboxylic acid |
| 160700 | 47910 | 0.93 | [M+H]+ | 120.0651 | THREONINE |
| 160700 | 47910 | 0.93 | [M+H]+ | 120.0651 | α-Methyl-DL-serine |
| 150000 | 47610 | 0.93 | [M+H]+ | 120.0651 | Threonine |
| 53720 | 8687 | 0.83 | [M+H]+ | 191.1022 | DIAMINOPIMELATE |
| 53720 | 8687 | 0.83 | [M+H]+ | 191.1022 | Diaminopimelic acid |
| 154000 | 25940 | 0.78 | [M+H]+ | 146.1648 | SPERMIDINE |

Note: POS, positive; LSW, liushenwan.

**Table S5. Quantification of Liushenwan (NEG mode)**

| **Area** | **Height** | **Retention Time** | **Adduct / Charge** | **Found At Mass** | **Library Hit** |
| --- | --- | --- | --- | --- | --- |
| 219400000 | 13500000 | 15.18 | [M-H]- | 498.29 | FOSA (perfluorooctane sulfonamide) (neg) |
| 39700000 | 5858000 | 11.45 | [M+FA-H]- | 461.2192 | Arenobufagin |
| 33350000 | 5419000 | 10.6 | [M+FA-H]- | 461.2198 | Arenobufagin |
| 24330000 | 5302000 | 12.56 | [M-H]- | 503.2305 | Madecassic acid |
| 22270000 | 1835000 | 13.83 | [2M-H]- | 1029.5813 | Taurocholic acid |
| 19910000 | 3151000 | 14.08 | [M-H]- | 464.3026 | Glycocholic acid |
| 17060000 | 3313000 | 11.17 | [M-H]- | 165.057 | Ethylparaben |
| 14260000 | 2722000 | 9.52 | [M-H]- | 173.0829 | SUBERATE |
| 12260000 | 2586000 | 12.63 | [M-H]- | 512.2706 | N-MeFOSA (N-methyl perfluoro-1-octane sulfonamide) (neg) |
| 9694000 | 1799000 | 14.75 | [M-H]- | 407.2814 | Cholan-24-oic acid |
| 8993000 | 2090000 | 14.08 | [2M-H]- | 929.6143 | GLYCOCHOLATE |
| 8862000 | 1764000 | 14.09 | [M]- | 465.3068 | Cholesterol sulfate |
| 7278000 | 1427000 | 14.61 | [M+FA-H]- | 437.2921 | Blinin +HCOOH |
| 6393000 | 627600 | 15.15 | [2M-H]- | 997.5889 | Taurodeoxycholic acid |
| 6022000 | 1047000 | 14.75 | [M+FA-H]- | 453.2869 | Cholan-24-oic acid |
| 5778000 | 1135000 | 15.48 | [M-H]- | 391.2858 | Deoxycholic acid |
| 4920000 | 1375000 | 17.09 | [M-H]- | 281.2495 | Vaccenic acid |
| 4390000 | 1194000 | 16.65 | [M-H]- | 279.234 | Linoleic acid |
| 4092000 | 927300 | 14.61 | [M-H]- | 391.2863 | URSODEOXYCHOLATE |
| 3049000 | 612800 | 14.59 | [M-H]- | 517.2853 | A5Sibiricose A5 |
| 3026000 | 548800 | 10.61 | [M-H]- | 415.2144 | Arenobufagin |
| 2054000 | 508700 | 15.41 | [M+FA-H]- | 437.2916 | Deoxycholic acid |
| 1908000 | 426300 | 14.47 | [M-H]- | 435.2762 | Polydatin +HCOOH |
| 1646000 | 423100 | 10.68 | [M-H]- | 187.0988 | Azelaic acid |
| 1582000 | 311400 | 15.07 | [M-H]- | 531.3006 | Phytolaccagenin |
| 1519000 | 366500 | 13.19 | [M-H]- | 407.2821 | Cholan-24-oic acid |
| 1420000 | 317300 | 14.28 | [M-H]- | 405.2657 | 3-Oxocholic acid |
| 1401000 | 507800 | 11.8 | [M]- | 506.2489 | ADENOSINE TRIPHOSPHATE |
| 1377000 | 342000 | 0.97 | [2M-H]- | 391.1115 | Gluconic acid |
| 1355000 | 340000 | 13.19 | [M+FA-H]- | 453.2878 | Cholan-24-oic acid |
| 1354000 | 331600 | 10.17 | [M-H]- | 181.0515 | Ethyl 3,4-Dihydroxybenzoate |
| 1346000 | 311700 | 8.13 | [M-H]- | 159.0672 | 6-CARBOXYHEXANOATE |
| 1288000 | 355500 | 6.22 | [2M-H]- | 631.3444 | Isorhamnetin |
| 1258000 | 288100 | 12.21 | [M-H]- | 514.2858 | Taurocholic acid |
| 1234000 | 391600 | 13.72 | [M-H]- | 405.2658 | 3-Oxocholic acid |
| 1183000 | 225700 | 11.44 | [M-H]- | 415.2139 | Arenobufagin |
| 1111000 | 230800 | 14.28 | [M+FA-H]- | 451.2715 | Dipsacoside B +HCOOH |
| 1025000 | 257100 | 12.13 | [M-H]- | 445.2243 | CDP-ETHANOLAMINE |
| 781900 | 271500 | 17.09 | [2M-H]- | 563.5059 | Vaccenic acid |
| 775500 | 127500 | 9.28 | [M-H]- | 329.0314 | Pyrroloquinoline Quinone |
| 759600 | 196600 | 0.9 | [M-H]- | 124.0079 | TAURINE |
| 744700 | 233200 | 14.28 | [2M-H]- | 811.5399 | 3-Oxocholic acid |
| 696400 | 213200 | 13.72 | [M+FA-H]- | 451.2715 | Dipsacoside B +HCOOH |
| 681600 | 164200 | 12.45 | [M-H]- | 464.3033 | Glycocholic acid |
| 668200 | 168300 | 9.51 | [M-H]- | 522.2176 | GUANOSINE TRIPHOSPHATE |
| 561600 | 168100 | 16.94 | [M-H]- | 255.2341 | PALMITATE |
| 549800 | 146300 | 16.62 | [M-H]- | 303.2338 | Arachidonic acid |
| 528600 | 158400 | 16.06 | [M+FA-H]- | 421.2968 | Loganic acid |
| 446500 | 153800 | 16.65 | [2M-H]- | 559.4747 | Linoleic acid |
| 441000 | 159000 | 14.56 | [M-H]- | 389.27 | 7a-Hydroxy-3-oxo-5b-cholanoic acid |
| 434600 | 96870 | 9.86 | [M+FA-H]- | 477.2139 | Genistin |
| 426400 | 73630 | 19.51 | [M-H]- | 320.8875 | 8-Gingerol |
| 387900 | 105300 | 11.64 | [M-H]- | 201.1143 | SEBACATE |
| 372100 | 75440 | 13.2 | [M-H]- | 403.2517 | CORTISOL 21-ACETATE |
| 309100 | 25600 | 19.72 | [M-H]- | 178.9785 | 3-(4-HYDROXYPHENYL)PYRUVATE |
| 295200 | 67120 | 16.33 | [M-H2O-H]- | 277.2181 | Sulfamethazine |
| 279800 | 56820 | 7.35 | [M-H]- | 137.025 | 4-Hydroxybenzoic acid |
| 253600 | 83550 | 1.27 | [M-H]- | 89.0247 | GLYCERALDEHYDE |
| 247800 | 73430 | 1.11 | [M+FA-H]- | 387.116 | TREHALOSE |
| 233900 | 33880 | 6.57 | [M-H]- | 145.0514 | ADIPATE |
| 230400 | 38740 | 16.9 | [M-H2O-H]- | 763.5543 | Ic Momordin Ic |
| 226000 | 41230 | 19.62 | [M]- | 116.9285 | MESOXALATE |
| 214000 | 80950 | 13.71 | [M-H]- | 448.3082 | Glycoursodeoxycholic acid |
| 200600 | 47690 | 15.76 | [M-H]- | 297.2444 | Nonadecanoic acid |
| 193200 | 36420 | 4.91 | [M-H]- | 267.0749 | Inosine |
| 187400 | 56700 | 16.06 | [M-H]- | 375.2917 | LITHOCHOLATE |
| 182500 | 47410 | 19.51 | [M+TFA-H]- | 434.8723 | 8-Gingerol |
| 178400 | 42300 | 17.7 | [M-H]- | 283.2653 | Ethyl hexadecanoate1 |
| 170600 | 39390 | 10.96 | [M-H]- | 431.2087 | Genistin |
| 169500 | 40040 | 10.86 | [M-H]- | 487.1991 | Asiatic acid |
| 168400 | 40800 | 7.52 | [M-H]- | 217.0992 | N-ACETYLSEROTONIN |
| 151400 | 35870 | 9.05 | [M+FA-H]- | 619.304 | Toosendanin +HCOOH |
| 150100 | 38490 | 8.73 | [M-H]- | 241.0994 | Thymidine |
| 138500 | 20450 | 13.1 | [M-H]- | 464.3033 | Glycocholic acid |
| 123900 | 10270 | 8.97 | [M-H]- | 171.0669 | 3-DEHYDROSHIKIMATE |
| 119500 | 29120 | 9.11 | [M-H]- | 165.0563 | L-3-Phenyllactic acid |
| 115200 | 27090 | 7.55 | [M-H]- | 178.0517 | HIPPURATE |
| 112600 | 25650 | 12.93 | [M-H]- | 423.2763 | Ginkgolide B |
| 111600 | 23930 | 8.13 | [M-H]- | 160.0702 | AMINOADIPATE |
| 110400 | 29960 | 9.81 | [M-H]- | 520.2284 | N-MeFHpSAA (N-methyl perfluoro-1-heptane sulfonamido acetic acid) (neg) |
| 109900 | 31090 | 8.24 | [2M-H]- | 687.408 | Maltitol |
| 103600 | 23500 | 7.72 | [M-H]- | 151.0407 | XANTHINE |
| 101600 | 24340 | 19.62 | [M-H]- | 115.9209 | VALINE |
| 99390 | 20830 | 7.35 | [M-H]- | 93.0348 | Chloroacetic acid |
| 94260 | 27170 | 1.11 | [M-H]- | 341.1104 | TREHALOSE |
| 92170 | 35360 | 16.06 | [2M-H]- | 751.5901 | LITHOCHOLATE |
| 90030 | 24850 | 13.14 | [M-H]- | 229.1454 | Dodecanedioic acid |
| 83250 | 26280 | 16.29 | [M-H]- | 227.2025 | Resveratrol |
| 78700 | 11010 | 19.44 | [M-H]- | 515.3067 | A 3,5-Dicaffeoyl quinic acid |
| 77910 | 27580 | 1.24 | [M-H]- | 182.0835 | Normetanephrine |
| 75810 | 21740 | 16.38 | [M-H]- | 373.2752 | 3b-Hydroxy-5-cholenoic acid |
| 75760 | 17060 | 8.96 | [M-H]- | 471.2071 | Hederagenin |
| 74420 | 12090 | 16.78 | [M-H]- | 115.9208 | VALINE |
| 71530 | 17580 | 19.6 | [M-H]- | 110.9761 | Methyl sulfate |
| 70480 | 15310 | 10.94 | [M-H2O-H]- | 619.3051 | Toosendanin +HCOOH |
| 68120 | 14100 | 8.1 | [M-H]- | 192.0679 | Phenylacetylglycine |
| 65930 | 7775 | 9.73 | [M-H]- | 171.0671 | BETA-GLYCEROPHOSPHATE |
| 58700 | 14630 | 8.79 | [M-H]- | 487.2688 | Asiatic acid |
| 55800 | 9476 | 1.47 | [M-H]- | 117.0197 | METHYLMALONATE |
| 55080 | 15360 | 16.88 | [M-H]- | 809.56 | Digitoxin +HCOOH |
| 51750 | 20480 | 0.85 | [2M+K-2H]- | 536.887 | Γ-Glutamate-Cysteine |
| 50080 | 17940 | 1.27 | [M-H]- | 191.0208 | Citric acid |
| 46970 | 3946 | 17.39 | [M-H]- | 116.9279 | MESOXALATE |
| 46930 | 8285 | 5.56 | [M-H]- | 164.0724 | L-Phenylalanine |
| 42840 | 18010 | 0.84 | [2M+Na-2H]- | 520.9124 | Γ-Glutamate-Cysteine |
| 42580 | 9710 | 17.83 | [M-H]- | 309.2808 | Sulfadoxine |
| 35070 | 9000 | 7.97 | [M-H2O-H]- | 174.0569 | 2-Guanidinobenzimidazole |
| 34260 | 3687 | 8.42 | [M+TFA-H]- | 471.2075 | Corosolic acid |
| 29280 | 5300 | 1.38 | [2M]- | 180.0677 | TYROSINE |
| 26600 | 7088 | 16.02 | [M-H]- | 295.2288 | 17a-Ethynylestradiol |
| 26370 | 4566 | 9.12 | [M+Na-2H]- | 653.2938 | Pseudoginsenoside-RT5 |
| 24110 | 4288 | 8.08 | [3M-H]- | 482.2245 | LITHOCHOLYLTAURINE |
| 23900 | 5369 | 5.77 | [M+AcO-H]- | 277.1204 | Pantetheine |
| 23420 | 3280 | 4.6 | [M-H]- | 231.0787 | Nalidixic acid |
| 22990 | 6837 | 16.03 | [M-H]- | 271.229 | ESTRADIOL-17ALPHA |
| 22420 | 1922 | 7.9 | [M-H]- | 165.0202 | Phthalic Acid_ |
| 22210 | 9847 | 0.88 | [M-H]- | 181.967 | L-Homocysteic acid |
| 21810 | 5377 | 19.4 | [M-H]- | 99.9258 | 1-AMINOCYCLOPROPANECARBOXYLATE |
| 20960 | 4874 | 8.58 | [M-H]- | 158.0827 | Isovalerylglycine |
| 20240 | 7107 | 1.24 | [M-H]- | 133.0146 | MALATE |
| 18400 | 3971 | 7.93 | [M-H]- | 121.0297 | 4-HYDROXYBENZALDEHYDE |
| 17530 | 1960 | 17.87 | [M-H]- | 132.9237 | N-Nitrosodiethanolamine |
| 15290 | 6018 | 0.88 | [M-H]- | 173.1053 | L(+)-Arginine |
| 12070 | 1859 | 17.84 | [M-H]- | 99.926 | 1-AMINOCYCLOPROPANECARBOXYLATE |
| 4364 | 745 | 18.15 | [M-H]- | 130.9446 | 4-Aminoindole |

Note: NEG, negative; LSW, liushenwan.

**Table S6. Quantification of Liushenwan (POS mode)**

| **Area** | **Height** | **Retention Time** | **Adduct / Charge** | **Found At Mass** | **Library Hit** |
| --- | --- | --- | --- | --- | --- |
| 60520000 | 12760000 | 13.55 | [M+H]+ | 443.2422 | Cinobufagin |
| 46450000 | 10340000 | 12.74 | [M+H]+ | 403.2474 | Ophiobolin B |
| 44060000 | 8852000 | 13.27 | [M+H]+ | 387.2531 | Bufalin |
| 37160000 | 7740000 | 12.38 | [M+H]+ | 445.258 | Bufotaline |
| 34960000 | 3553000 | 5.37 | [M+H]+ | 219.1491 | N-ACETYLSEROTONIN |
| 30790000 | 5650000 | 14.08 | [M+H]+ | 466.3164 | GLYCOCHOLATE |
| 24120000 | 5946000 | 13.6 | [M+H]+ | 385.2373 | Resibufogenin |
| 23400000 | 1789000 | 13.84 | [M+H]+ | 516.2986 | Taurocholic acid |
| 19780000 | 1278000 | 15.18 | [M+H]+ | 500.3043 | Taurodeoxycholic acid |
| 14850000 | 2895000 | 12.12 | [M+H]+ | 403.2478 | Fusarielin A |
| 14250000 | 3188000 | 14.86 | [M+H]+ | 450.3221 | GLYCOCHENODEOXYCHOLATE |
| 13410000 | 3523000 | 16.64 | [M+H]+ | 239.2376 | Muscone |
| 13330000 | 3560000 | 10.54 | [M+H]+ | 403.2478 | Ophiobolin B |
| 9286000 | 1444000 | 15.48 | [M+H]+ | 415.2825 | Chenodeoxycholic acid +Na |
| 8861000 | 813600 | 13.82 | [2M+H]+ | 1031.592 | Taurocholic acid |
| 8620000 | 1001000 | 13.83 | [M+NH4]+ | 533.3253 | Taurocholic acid |
| 8084000 | 2220000 | 12.67 | [M+H]+ | 401.2324 | Ophiobolin A |
| 7892000 | 1922000 | 14.45 | [M+H]+ | 391.2848 | Nutriacholic acid |
| 7339000 | 1675000 | 15.67 | [M+H]+ | 459.3088 | AM-2233 |
| 6943000 | 344500 | 3.31 | [M+H]+ | 160.0753 | INDOLEACETALDEHYDE |
| 6898000 | 2006000 | 14.85 | [2M+H]+ | 899.6375 | GLYCOCHENODEOXYCHOLATE |
| 6737000 | 1455000 | 11.17 | [M+H]+ | 167.0706 | Ethyl 4-hydroxybenzoate |
| 6716000 | 1922000 | 12.73 | [2M+H]+ | 805.4882 | Dehydrolithocholic acid |
| 5625000 | 1320000 | 12.14 | [M+H]+ | 401.232 | Ophiobolin A |
| 5598000 | 638400 | 15.15 | [M+Na]+ | 522.2863 | Tauroursodeoxycholic acid +Na |
| 5386000 | 1160000 | 11.17 | [M+H]+ | 139.039 | 4-Hydroxybenzoic acid |
| 4977000 | 1308000 | 16.36 | [M+NH4]+ | 385.311 | Vitamin D3 |
| 4933000 | 881600 | 13.27 | [M+Na]+ | 409.2356 | CHOLATE |
| 4865000 | 532500 | 19.02 | [M+H]+ | 537.5356 | BETA-CAROTENE |
| 4793000 | 1243000 | 16.63 | [M+Na]+ | 261.2195 | FRUCTOSE 6-PHOSPHATE |
| 4690000 | 687500 | 14.1 | [M+Na]+ | 488.2983 | Sodium glycocholate |
| 4495000 | 513300 | 13.95 | [M+Na]+ | 538.281 | Sodium Taurocholate |
| 4220000 | 769500 | 12.74 | [M+Na]+ | 425.2297 | Ginkgolide B |
| 3609000 | 971600 | 17.83 | [M+Na]+ | 360.3245 | Etoxazole |
| 3529000 | 1081000 | 14.56 | [M+H]+ | 291.2324 | Androsterone |
| 3346000 | 685900 | 9.52 | [M+H]+ | 197.0782 | mCPP |
| 3312000 | 1062000 | 16.64 | [2M+H]+ | 477.4676 | Muscone |
| 3195000 | 319000 | 6.95 | [M+H]+ | 475.2399 | Amygdalin +NH3 |
| 3119000 | 434800 | 14.08 | [M+NH4]+ | 483.3427 | Glycocholic acid |
| 3096000 | 651600 | 9.52 | [M+H]+ | 157.0857 | Decanal |
| 2974000 | 750500 | 0.98 | [M+H]+ | 258.1106 | Glycerophosphocholine |
| 2960000 | 643900 | 16.64 | [M+H]+ | 357.3006 | OLEOYL-GLYCEROL |
| 2831000 | 635300 | 16.27 | [M+H]+ | 377.2668 | Etofenprox |
| 2744000 | 954300 | 14.67 | [M+Na]+ | 472.3039 | Sodium glycodeoxycholate |
| 2677000 | 641200 | 13.61 | [M+Na]+ | 407.2191 | perfluoropropane sulfonamido propyl dimethyl quaternary amine propanoate (neg,pos) |
| 2636000 | 836100 | 14.56 | [M+Na]+ | 313.2142 | 16-Dehydroprogesterone |
| 2616000 | 679700 | 13.71 | [M+H]+ | 289.2165 | Dehydroepiandrosterone |
| 2559000 | 616800 | 10.79 | [M+H]+ | 415.211 | 7-Hydroxymitragyline |
| 2547000 | 546900 | 12.73 | [M+H]+ | 385.2373 | Resibufogenin |
| 2506000 | 315200 | 5.42 | [M+H]+ | 205.1332 | Bufotenine |
| 2480000 | 315800 | 15.24 | [M+H]+ | 279.2323 | Alpha-Linolenic acid |
| 2455000 | 446300 | 19.5 | [M+H]+ | 84.9597 | CYCLOPENTANONE |
| 2424000 | 222400 | 15.15 | [2M+H]+ | 999.6023 | Taurodeoxycholic acid |
| 2340000 | 570000 | 11.92 | [M+H]+ | 225.11 | METHYL JASMONATE |
| 1921000 | 376600 | 16.69 | [M+H]+ | 303.2305 | GLYCEROL-MYRISTATE |
| 1839000 | 482900 | 14.41 | [M+H]+ | 505.3135 | Dipyridamole |
| 1820000 | 244000 | 4.87 | [M+H]+ | 177.1018 | Serotonin |
| 1752000 | 369800 | 9.52 | [M+H]+ | 175.0963 | Suberic acid |
| 1725000 | 86860 | 3.32 | [M+NH4]+ | 177.1018 | Serotonin |
| 1710000 | 300600 | 12.12 | [M+Na]+ | 425.2292 | Clindamycin |
| 1658000 | 368900 | 17.87 | [M+H]+ | 331.2613 | Sclareol +Na |
| 1656000 | 317200 | 14.62 | [M+H]+ | 415.2824 | Chenodeoxycholic acid +Na |
| 1649000 | 503500 | 14.56 | [M+H]+ | 255.211 | Palitantin |
| 1632000 | 306100 | 15.02 | [M+NH4]+ | 517.3311 | Taurodeoxycholic acid |
| 1597000 | 528700 | 13.6 | [2M+H]+ | 769.4671 | Resibufogenin |
| 1584000 | 479800 | 14.56 | [M+NH4]+ | 308.2587 | Androsterone, MNH4 |
| 1538000 | 282800 | 10.61 | [2M+NH4]+ | 850.4738 | Arenobufagin |
| 1479000 | 376300 | 17.09 | [M+H]+ | 283.2637 | ELAIDATE |
| 1343000 | 229900 | 11.46 | [2M+NH4]+ | 850.473 | Arenobufagin |
| 1321000 | 322300 | 18.48 | [M]+ | 311.2954 | RIBOSE 1,5-BISPHOSPHATE |
| 1298000 | 404800 | 16.5 | [M+H]+ | 331.2844 | Monopalmitin |
| 1294000 | 371800 | 16.5 | [M+H]+ | 282.2795 | PETROSELINATE |
| 1193000 | 259600 | 10.18 | [M+CH3OH+H]+ | 431.2066 | Ruscogenin |
| 1101000 | 363700 | 16.34 | [M+NH4]+ | 256.2641 | Myristic acid(D27) |
| 1092000 | 396100 | 0.89 | [M+H]+ | 175.1189 | ARGININE |
| 1075000 | 250700 | 12.23 | [M+Na]+ | 479.2037 | Isochlortetracyclin |
| 1003000 | 241000 | 0.91 | [M+H]+ | 126.0216 | TAURINE |
| 964800 | 220100 | 9.86 | [M]+ | 433.2218 | Mizolastine |
| 925700 | 295700 | 13.71 | [2M+H]+ | 577.4245 | Dehydroepiandrosterone |
| 912100 | 204000 | 12.19 | [M+H]+ | 386.213 | Buspirone |
| 869000 | 63980 | 19.35 | [M+H]+ | 163.1329 | 5-HYDROXYLYSINE |
| 861000 | 193400 | 16.64 | [M+H]+ | 339.29 | ERUCATE |
| 855800 | 262000 | 12.18 | [2M+H]+ | 829.4162 | Chenodeoxycholic acid +Na |
| 808500 | 216600 | 16.98 | [M+H]+ | 284.2949 | Octadecanamide |
| 795000 | 240600 | 14.95 | [M+NH4]+ | 356.295 | Kirenol +NH3 |
| 785200 | 179300 | 17.89 | [M+H]+ | 423.3238 | Fludrocortisone-acetate |
| 765600 | 223200 | 10.53 | [M+Na]+ | 425.2288 | Ginkgolide B |
| 750100 | 192600 | 13.38 | [M+H]+ | 399.216 | N-TAmP-FBSA (N-trimethylammoniopropyl perfluorobutanesulfonamide) (pos) |
| 713500 | 109400 | 18.15 | [M]+ | 585.2706 | BILIRUBIN |
| 709800 | 255000 | 10.54 | [2M+H]+ | 805.4874 | panepocyclinol H/029-01 |
| 674800 | 168600 | 14.92 | [M+H]+ | 301.1413 | All-trans-retinoic acid |
| 658200 | 168600 | 13.19 | [M+H]+ | 391.2841 | Nutriacholic acid |
| 654900 | 160200 | 9.76 | [M+H]+ | 417.2277 | Arenobufagin |
| 652400 | 185100 | 12.12 | [2M+H]+ | 805.4879 | Dehydrolithocholic acid |
| 643800 | 135500 | 17.09 | [M+H]+ | 327.2272 | Ajmaline |
| 643100 | 157500 | 9.22 | [M+H]+ | 231.1228 | Dodecanedioic acid |
| 639300 | 171100 | 17.61 | [M+H]+ | 385.3468 | Vitamin D3 |
| 611900 | 153700 | 0.79 | [M+H]+ | 102.9695 | Isovaleric acid-1 |
| 596500 | 148400 | 13.17 | [M+H]+ | 494.3845 | Paraherquamide A |
| 578800 | 176400 | 16.19 | [M+H]+ | 387.2872 | Tioconazole |
| 574200 | 188600 | 14.92 | [M+NH4]+ | 410.3263 | Ursodeoxycholic acid, [M+NH4]+ |
| 563000 | 125000 | 7.52 | [M+H]+ | 219.113 | N-Acetylserotonin |
| 562700 | 150100 | 14.35 | [M+Na]+ | 502.3142 | Fexofenadine |
| 554400 | 138800 | 13.45 | [M+Na]+ | 581.3085 | MENAQUINONE |
| 553500 | 158500 | 17.37 | [M+H]+ | 413.2662 | Ziprasidone |
| 549000 | 133900 | 10.75 | [M+H]+ | 431.2064 | Ruscogenin |
| 548200 | 91500 | 19.5 | [M+CH3OH+H]+ | 116.9856 | 4-Methylvaleric Acid |
| 546500 | 141400 | 11.83 | [M]+ | 419.2334 | Sucralose +Na |
| 525400 | 167100 | 0.91 | [2M+H]+ | 251.0368 | TAURINE |
| 520300 | 141000 | 15.8 | [M+H]+ | 283.2632 | ELAIDATE |
| 513600 | 139100 | 0.96 | [M+CH3OH+H]+ | 104.1062 | AMINOISOBUTANOATE |
| 508100 | 133400 | 11.92 | [M+H]+ | 139.075 | 2-n-Pentylfuran |
| 485700 | 96380 | 9.52 | [M+H]+ | 139.0746 | ISOPHORONE |
| 452900 | 114900 | 0.79 | [M+H]+ | 130.9644 | 5-Oxo-2-tetrahydrofurancarboxylic acid |
| 452400 | 90450 | 0.97 | [M+H]+ | 179.0544 | Gluconolactone |
| 405800 | 97910 | 9.22 | [M+H]+ | 271.1152 | HEPTADECANOATE |
| 399200 | 127900 | 1.24 | [M+H]+ | 184.0967 | EPINEPHRINE |
| 390000 | 85920 | 11.26 | [M+H]+ | 425.2287 | Clindamycin |
| 389300 | 146500 | 14.67 | [M+NH4]+ | 467.348 | GLYCOCHENODEOXYCHOLATE |
| 388800 | 117500 | 15.84 | [M+H]+ | 593.345 | Hexobendine |
| 368200 | 100600 | 17.37 | [M+H]+ | 391.2843 | PHOSPHORIBOSYL PYROPHOSPHATE |
| 367700 | 73070 | 17.08 | [M+H]+ | 383.3303 | Campesterol |
| 363000 | 132100 | 0.87 | [M+NH4]+ | 131.1289 | AGMATINE SULFATE |
| 358500 | 100400 | 18.3 | [M+H]+ | 285.2791 | Ethyl hexadecanoate1 |
| 356100 | 19400 | 2.51 | [M+H]+ | 235.1437 | 5-Methoxytryptophan |
| 354500 | 80830 | 16.33 | [M+H]+ | 301.2138 | RETINOATE |
| 311100 | 52700 | 10.3 | [M+H]+ | 163.1325 | 5-HYDROXYLYSINE |
| 301500 | 65130 | 11.01 | [M+H]+ | 441.224 | Sertindole |
| 298000 | 81080 | 17.41 | [M+H]+ | 393.2976 | DEOXYCHOLATE |
| 285300 | 16610 | 6.81 | [M+H]+ | 163.1325 | 5-HYDROXYLYSINE |
| 279800 | 14230 | 2.43 | [M+H]+ | 124.0751 | Isonicotinic acid |
| 276400 | 57700 | 0.97 | [M]+ | 197.0655 | Gluconic acid |
| 270200 | 55440 | 0.96 | [M+CH3OH+H]+ | 133.0489 | 2,3-Pentanedione |
| 257000 | 42100 | 10.24 | [M+H]+ | 287.1465 | Luteolin |
| 244500 | 46920 | 17.51 | [M+H]+ | 417.3365 | Tigogenin |
| 243300 | 60500 | 8.79 | [M+H]+ | 489.2825 | Vardenafil |
| 241100 | 43940 | 4.76 | [M+CH3OH+H]+ | 235.1438 | 5-Methoxytryptophan |
| 239300 | 41430 | 7.52 | [M+H]+ | 160.0754 | INDOLEACETALDEHYDE |
| 225200 | 24640 | 2.02 | [M+H]+ | 132.1013 | LEUCINE |
| 211100 | 28930 | 4.65 | [M+H]+ | 148.0752 | 3-METHYL-2-OXINDOLE |
| 209600 | 23160 | 2.18 | [M+H]+ | 137.0453 | 6-Hydroxypurine |
| 202900 | 49070 | 9.22 | [M+H]+ | 213.112 | Prohexadione |
| 196600 | 35990 | 5.56 | [M+H]+ | 166.0858 | PHENYLALANINE |
| 194800 | 46270 | 8.13 | [M+CH3OH+H]+ | 183.0628 | METHYL VANILLATE |
| 174100 | 42450 | 10 | [M+H]+ | 376.2847 | Tuberostemonine |
| 172200 | 41390 | 8.13 | [M+H]+ | 143.07 | 1-Nonanal |
| 167600 | 13710 | 6.03 | [M+H]+ | 163.1326 | 5-HYDROXYLYSINE |
| 161300 | 37070 | 8.84 | [M+H]+ | 432.2801 | PEG-9mer Ammonium adduct |
| 159700 | 12510 | 8.41 | [M+H]+ | 163.1328 | 5-HYDROXYLYSINE |
| 153600 | 10110 | 18.2 | [M+H]+ | 163.1325 | 5-HYDROXYLYSINE |
| 151600 | 34930 | 9.84 | [M+H]+ | 523.3163 | N-HOEAmP-FPeSE (N-hydroxyethyldimethylammoniopropyl perfluoropentanesulfonamidoethanol) (pos) |
| 149300 | 35030 | 0.78 | [M+H]+ | 86.9917 | DIACETYL |
| 148000 | 58330 | 0.88 | [M+Na]+ | 148.0035 | Isatin |
| 146400 | 34090 | 9.97 | [M+H]+ | 113.0595 | Sorbic acid |
| 143900 | 9197 | 6.73 | [M+H]+ | 219.1489 | N-Acetylserotonin |
| 142000 | 33710 | 9.15 | [M+H]+ | 476.3064 | PEG-10mer Ammonium adduct |
| 140300 | 18400 | 13.12 | [M+H]+ | 163.1328 | 5-HYDROXYLYSINE |
| 139500 | 23350 | 5.57 | [M+H]+ | 174.0909 | 4-QUINOLINECARBOXYLATE |
| 123100 | 26550 | 8.47 | [M+H]+ | 388.2542 | PEG-8mer Ammonium adduct |
| 121300 | 12570 | 6.04 | [M+H]+ | 191.0889 | Crisitine |
| 120600 | 36150 | 16.06 | [M+H]+ | 399.2864 | N-TAmP-FBSA (N-trimethylammoniopropyl perfluorobutanesulfonamide) (pos) |
| 120100 | 14420 | 16.01 | [M+H]+ | 163.1326 | 5-HYDROXYLYSINE |
| 117400 | 26430 | 15.62 | [M+H]+ | 701.3731 | Gingerglycolipid B +Na |
| 113500 | 22410 | 6.85 | [M+H]+ | 160.0753 | INDOLEACETALDEHYDE |
| 108800 | 24890 | 8.13 | [M+H]+ | 161.0804 | 8-Hydroxyoctanoic acid |
| 106000 | 24580 | 8.13 | [M+H]+ | 125.0593 | GUAIACOL |
| 105700 | 16390 | 8.44 | [M+H]+ | 372.2532 | Kristallviolett |
| 104200 | 22780 | 10.13 | [M+H]+ | 169.0858 | Griffonilide |
| 95310 | 32920 | 1.08 | [M+H]+ | 116.0699 | Proline |
| 95250 | 20440 | 8.11 | [M+H]+ | 194.081 | Phenylacetylglycine |
| 94820 | 23100 | 15.62 | [M+H]+ | 473.324 | N-HOEAmP-FBSE (N-hydroxyethyldimethylammoniopropyl perfluorobutanesulfonamidoethanol) (pos) |
| 90840 | 4502 | 1.6 | [M+H]+ | 284.0987 | guanosine |
| 90230 | 6881 | 19.77 | [M]+ | 131.9499 | 2-Butanol |
| 85460 | 13510 | 9.83 | [M+H]+ | 524.3119 | Penfluridol |
| 85440 | 21260 | 9.35 | [M+H]+ | 427.2953 | LANOSTEROL |
| 84860 | 16250 | 5.59 | [M+H]+ | 285.09 | Psilocybin |
| 84690 | 5933 | 1.88 | [M+H]+ | 269.0877 | INOSINE |
| 84500 | 8133 | 2.27 | [M+H]+ | 130.0491 | OXOPROLINE |
| 77310 | 18990 | 8.92 | [M+H]+ | 337.1543 | S-Nitrosoglutathione |
| 76330 | 18750 | 10.06 | [M+H]+ | 374.2686 | Prochlorperazine |
| 74280 | 14360 | 1.37 | [M+H]+ | 182.0804 | TYROSINE |
| 73950 | 9022 | 1.8 | [M+H]+ | 150.0908 | 4-Dimethylaminobenzaldehyde |
| 73560 | 29750 | 0.85 | [M+H]+ | 430.9129 | Bicalutamide |
| 72700 | 17310 | 9.76 | [M+Na]+ | 439.2091 | panepocyclinol D/F23-25-P4 |
| 71390 | 22550 | 1.11 | [M+H]+ | 146.117 | DEOXYCARNITINE |
| 70880 | 21230 | 1.17 | [M]+ | 145.1077 | CAPRYLATE |
| 70220 | 16170 | 9.34 | [M+H]+ | 438.2628 | Fluphenazine |
| 67790 | 15070 | 8.9 | [M+H]+ | 306.1239 | CYTIDINE 2',3'-CYCLIC PHOSPHATE |
| 65270 | 14240 | 9.34 | [M+NH4]+ | 433.2649 | Mizolastine |
| 64570 | 15130 | 10.09 | [M+Na]+ | 441.2246 | Aminopterin |
| 64380 | 14750 | 9.66 | [M+H]+ | 321.0868 | Ethaboxam |
| 62890 | 10100 | 1.43 | [M+H]+ | 121.0642 | MERCAPTOPYRUVATE |
| 61450 | 3258 | 1.6 | [M+K]+ | 152.0559 | GUANINE |
| 60960 | 4314 | 7.99 | [M+H]+ | 100.0751 | 1,2-Dichloroethane |
| 60320 | 14720 | 10.27 | [M+H]+ | 399.1793 | N-TAmP-FBSA (N-trimethylammoniopropyl perfluorobutanesulfonamide) (pos) |
| 57410 | 26460 | 0.87 | [M+H]+ | 169.9852 | 1-METHYL-L-HISTIDINE |
| 56280 | 11770 | 9.65 | [M+NH4]+ | 521.3002 | N-TAmP-FPeSAP (N-trimethylammoniopropyl perfluoropentanesulfonamido propanoic acid) (pos) |
| 54780 | 19900 | 1.11 | [M+H]+ | 127.072 | Melamine |
| 53870 | 12150 | 8.15 | [M+H]+ | 399.2635 | N-TAmP-FBSA (N-trimethylammoniopropyl perfluorobutanesulfonamide) (pos) |
| 50140 | 3469 | 19.87 | [M+H]+ | 109.0756 | 1,3-Phenylenediamine |
| 49400 | 10700 | 8.43 | [M+H]+ | 252.102 | 7-Aminonitrazepam |
| 47940 | 16300 | 1.11 | [M+H]+ | 203.15 | N,N-DIMETHYL-ARGININE |
| 43660 | 3907 | 2.75 | [M+H]+ | 158.0915 | N-ACETYLPROLINE |
| 42680 | 3051 | 4.53 | [M+H]+ | 100.075 | ALLYL ISOTHIOCYANATE |
| 37210 | 4810 | 1.64 | [M+H]+ | 146.0917 | 4-GUANIDINOBUTANOATE |
| 34350 | 5950 | 5.6 | [M+H]+ | 100.075 | ALLYL ISOTHIOCYANATE |
| 32100 | 10850 | 1.21 | [M+H]+ | 131.1173 | N-ACETYLPUTRESCINE |
| 25040 | 3405 | 1.42 | [M+NH4]+ | 138.0907 | TYRAMINE |
| 23620 | 3893 | 10.07 | [M+H]+ | 84.9593 | CYCLOPENTANONE |
| 14300 | 2659 | 1.46 | [M+H]+ | 101.0224 | Tiglic acid |
| 11390 | 1894 | 2.54 | [M+H]+ | 100.0751 | 1,2-Dichloroethane |
| 9822 | 1201 | 4.47 | [M+H]+ | 279.1585 | Dibutyl phthalate |
| 8571 | 861 | 2.22 | [M+H]+ | 91.038 | DL-Glyceraldehyde |
| 5309 | 683 | 2.9 | [M+H]+ | 205.0851 | Patchouli alcohol (loss H20) |
| 4982 | 1032 | 2.93 | [M+H]+ | 149.0224 | TRANS-CINNAMATE |
| 3650 | 549 | 2.71 | [M+H]+ | 279.1588 | Dibutyl phthalate |
| 3009 | 431 | 2.17 | [M+CH3OH+H]+ | 107.0693 | o-Xylene |
| 1968 | 832 | 19.87 | [M+H]+ | 61.0282 | Urea |

Note: POS, positive; LSW, liushenwan.
